# SPTAN1/NUMB axis senses cell density to restrain cell growth and oncogenesis through Hippo signaling

**DOI:** 10.1172/JCI168888

**Published:** 2023-10-16

**Authors:** Dongxue Su, Yuxi Li, Weiji Zhang, Huan Gao, Yao Cheng, Yongqiang Hou, Junhong Li, Yi Ye, Zhangjian Lai, Zhe Li, Haitao Huang, Jiaxin Li, Jinhuan Li, Mengyu Cheng, Cheng Nian, Na Wu, Zhien Zhou, Yunzhi Xing, Yu Zhao, He Liu, Jiayu Tang, Qinghua Chen, Lixin Hong, Wengang Li, Zhihai Peng, Bin Zhao, Randy L. Johnson, Pingguo Liu, Wanjin Hong, Lanfen Chen, Dawang Zhou

**Affiliations:** 1 State Key Laboratory of Cellular Stress Biology, Innovation Center for Cell Signaling Network, School of Life Sciences, Xiamen University and; 2 Department of Hepatobiliary and Pancreatic and Organ Transplantation Surgery, Xiang’an Hospital of Xiamen University, School of Medicine, Xiamen University, Xiamen, Fujian, China.; 3 The MOE Key Laboratory of Biosystems Homeostasis and Protection, Zhejiang Provincial Key Laboratory for Cancer Molecular Cell Biology, and Innovation Center for Cell Signaling Network, Life Sciences Institute, Zhejiang University, Hangzhou, Zhejiang, China.; 4 Department of Cancer Biology, University of Texas, M.D. Anderson Cancer Center, Houston, Texas, USA.; 5 Fujian Provincial Key Laboratory of Chronic Liver Disease and Hepatocellular Carcinoma, Department of Hepatobiliary Surgery, Zhongshan Hospital, School of Medicine, Xiamen University, Xiamen, Fujian, China.; 6 Institute of Molecular and Cell Biology, Agency for Science, Technology, and Research (ASTAR), Singapore, Singapore.

**Keywords:** Cell Biology, Oncology, Cancer, Liver cancer, Signal transduction

## Abstract

The loss of contact inhibition is a key step during carcinogenesis. The Hippo–Yes-associated protein (Hippo/YAP) pathway is an important regulator of cell growth in a cell density–dependent manner. However, how Hippo signaling senses cell density in this context remains elusive. Here, we report that high cell density induced the phosphorylation of spectrin α chain, nonerythrocytic 1 (SPTAN1), a plasma membrane–stabilizing protein, to recruit NUMB endocytic adaptor protein isoforms 1 and 2 (NUMB1/2), which further sequestered microtubule affinity–regulating kinases (MARKs) in the plasma membrane and rendered them inaccessible for phosphorylation and inhibition of the Hippo kinases sterile 20–like kinases MST1 and MST2 (MST1/2). WW45 interaction with MST1/2 was thereby enhanced, resulting in the activation of Hippo signaling to block YAP activity for cell contact inhibition. Importantly, low cell density led to SPTAN1 dephosphorylation and NUMB cytoplasmic location, along with MST1/2 inhibition and, consequently, YAP activation. Moreover, double KO of NUMB and WW45 in the liver led to appreciable organ enlargement and rapid tumorigenesis. Interestingly, NUMB isoforms 3 and 4, which have a truncated phosphotyrosine-binding (PTB) domain and are thus unable to interact with phosphorylated SPTAN1 and activate MST1/2, were selectively upregulated in liver cancer, which correlated with YAP activation. We have thus revealed a SPTAN1/NUMB1/2 axis that acts as a cell density sensor to restrain cell growth and oncogenesis by coupling external cell-cell contact signals to intracellular Hippo signaling.

## Introduction

Cells within multicellular organisms are able to sense cell-cell contact and their density to control the proper tissue morphogenesis and organ size (1, 2). When cell density increases, contact inhibition can force proliferating cells into growth arrest. The loss of proliferation control when contact inhibition is abnormally regulated is a key step in the initiation of various types of cancers (3). Although cell junction complexes have been shown to play an important role in contact inhibition, the underlying regulatory mechanisms of cell proliferation and oncogenesis remain poorly understood.

The Hippo pathway has been shown to play a vital role in the regulation of contact inhibition of cell growth by inactivating YAP/TAZ signaling (4–8). This pathway is composed of a core kinase cascade including sterile 20–like kinases MST1 and MST2 (MST1/2) in mammals, the homologs of *Drosophila* Hippo, their downstream large tumor suppressor kinases LATS1 and LATS2 (LATS1/2), and regulator Salvador (Sav1, also known as WW45) and Mps one binder 1 (MOB1). MST1/2 phosphorylate and activate the MOB1-LATS1/2 complex, which then phosphorylates downstream effectors YAP and its paralog TAZ, followed by their degradation or sequestration in the cytoplasm by 14-3-3 proteins. At low cell density (LCD) with limited cell-cell contact, Hippo signaling activation is low, and YAP and TAZ are predominantly located in the nucleus, where they interact with TEAD transcription factors to regulate proproliferative genes, thereby enabling cell proliferation. In contrast, at high cell density (HCD) with extensive cell-cell contacts, the Hippo pathway is activated and YAP/TAZ retained cytosolically and degraded, leading to cell growth arrest. Several molecules including spectrins have been shown to be recruited to adherens or tight junctions and serve as regulators of the Hippo pathway (9–15). Spectrins are cytoskeletal and scaffolding proteins, which have been shown to attach to the inner surface of the plasma membrane by interacting with the integral membrane proteins ankyrin, band 2.1, and band 4.1 or with membrane phosphatidylinositol lipids to maintain cell shape (16–22). Spectrins are proposed to regulate Hippo/YAP signaling in response to cell density and cortical tension in *Drosophila* (9, 10, 23). However, the mechanisms underlying spectrin modulation of YAP activity in mammals remain elusive.

NUMB is an evolutionarily conserved cell-fate determinant in *Drosophila* and mammals that is asymmetrically segregated in dividing cells and determines cell fates mainly through downregulation of NOTCH signaling (24–27). Mammalian NUMB genes are expressed in most tissues and alternatively spliced to produce 4 major isoforms that act in cell fate determination decisions (24, 27–37). NUMB endocytic adaptor protein isoforms 1 and 2 (NUMB1/2) have long phosphotyrosine-binding (PTB) domains and bind to the plasma membrane through unknown mechanisms, while NUMB3 and NUMB4 (NUMB3/4), which have truncated, spliced, short PTB domains, are mostly found in the cytoplasm. In addition, NUMB deficiency results in differentiation failure of precursor cells, leading to numerous diseases, including developmental defects and cancer (24, 38).

In this study, we demonstrate that NUMB1/2 acted to relay a cell density signal from the plasma membrane to the intracellular Hippo kinases MST1/2 for growth inhibition. We found that NUMB1/2 acted downstream of the plasma membrane–stabilizing protein spectrin α chain, nonerythrocytic 1 (SPTAN1) to regulate YAP through the Hippo kinases MST1/2 in response to cell density cues. Increased cell density gradually enhanced SPTAN1 phosphorylation at tyrosine 1176 (Tyr1176) to recruit NUMB1/2 through their PTB domain, and NUMB1/2 further sequestered the MST1/2 inhibitor microtubule affinity–regulating kinase (MARK) at the cell membrane. At LCD, SPTAN1 was dephosphorylated and NUMB1/2 were released from SPTAN1 into the cytoplasm, resulting in competition between MARK and WW45, a positive regulator of MST1/2, to phosphorylate and inhibit MST1/2. Importantly, LCD or double-KO of WW45 and NUMB resulted in strong inhibition of MST1/2 by MARK, which, in the liver, led to appreciable organ enlargement and rapid tumorigenesis. Moreover, NUMB3/4, with their truncated PTB domains that render them unable to interact with SPTAN1 and activate MST1/2, were preferentially upregulated in liver cancer, along with a reduction of phosphorylated SPTAN1 (p-SPTAN1), which correlated with YAP activation. Thus, selective upregulation of NUMB3/4 expression along with a reduction of p-SPTAN1 might be an important mechanism of suppression of MST1/2 activity, leading to loss of cell-cell contact inhibition of cell growth during the initiation and progression of cancer. Our findings demonstrate a critical role of the SPTAN1/NUMB axis in cell density sensing, shedding light on the essential role of NUMB isoform splicing in the loss of cell contact inhibition and the initiation of cancer.

## Results

### The subcellular location of NUMB1/2 is altered to modulate YAP activity in response to cell density cues.

**Figure 1:**
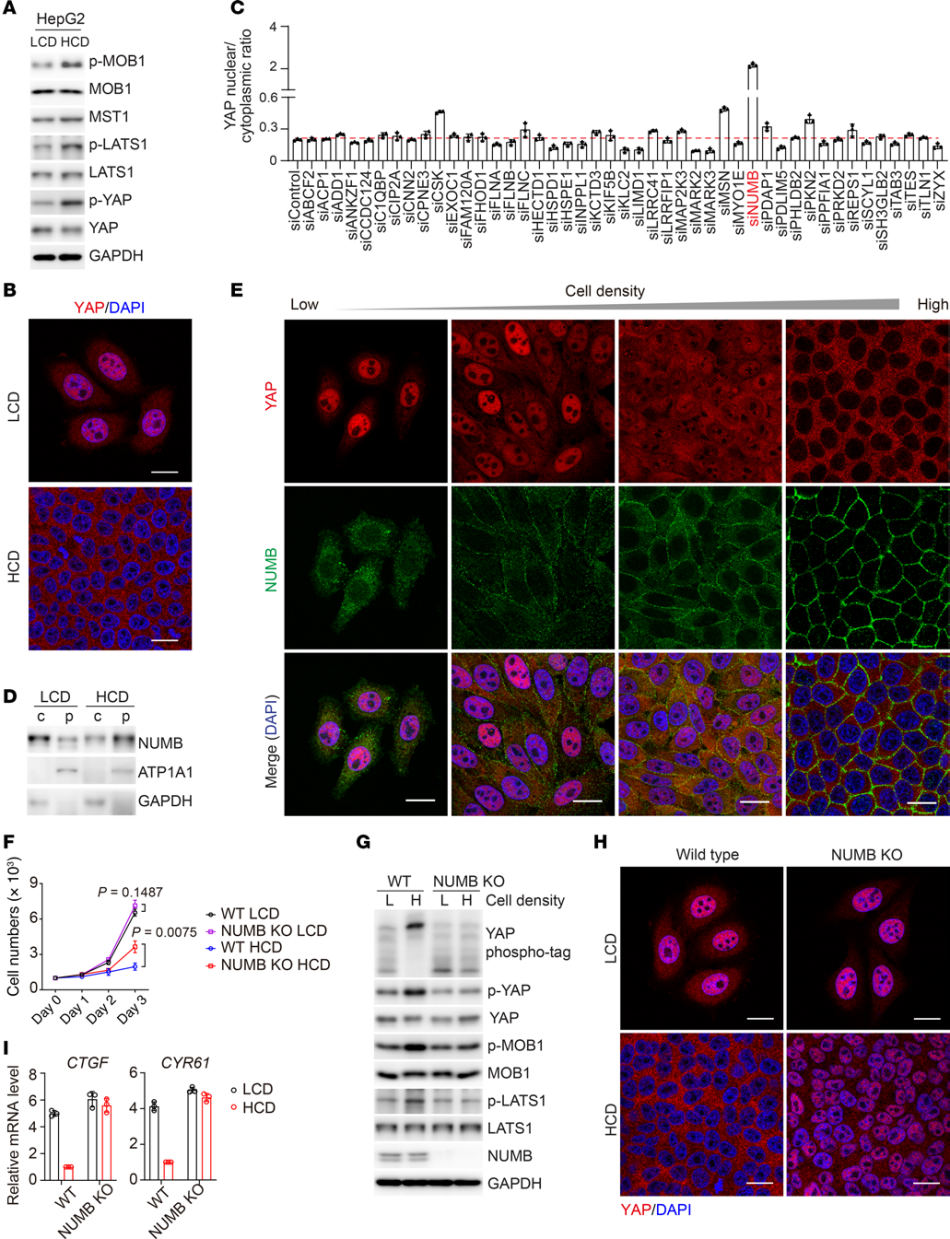
NUMB proteins sense cell density to modulate YAP activity. (**A** and **B**) Immunoblot analysis of the indicated proteins (**A**) and immunofluorescence staining for YAP (**B**) in HepG2 cells cultured at LCD or HCD. (**C**) Fluorescence intensities (quantified by ImageJ) of nucleus-to-cytoplasm ratios of YAP in HepG2 cells transfected with the indicated siRNAs followed by immunofluorescence staining. (**D**) Immunoblot analysis of the indicated proteins in the cytoplasmic fraction (c) and the plasma membrane fraction (p) of HepG2 cells cultured at LCD or HCD. (**E**) Immunofluorescence staining for YAP (red) and NUMB (green) in HepG2 cells cultured at different cell densities. (**F**) Growth curve of WT and NUMB-KO HepG2 cells cultured at LCD or HCD. Data are presented the mean ± SD from biological triplicate experiments. *P* values were assessed by 2-tailed, unpaired Student’s *t* test. (**G**–**I**) Phospho-tag and SDS-PAGE analysis (**G**), immunofluorescence staining (**H**), quantitative PCR (qPCR) analysis (**I**) of the indicated proteins in WT and NUMB-KO HepG2 cells cultured at LCD or HCD. Each bar represents the mean ± SD from biological triplicate experiments (**C** and **I**). Scale bars: 20 μm (**B**, **E**, and **H**).

**Figure 2:**
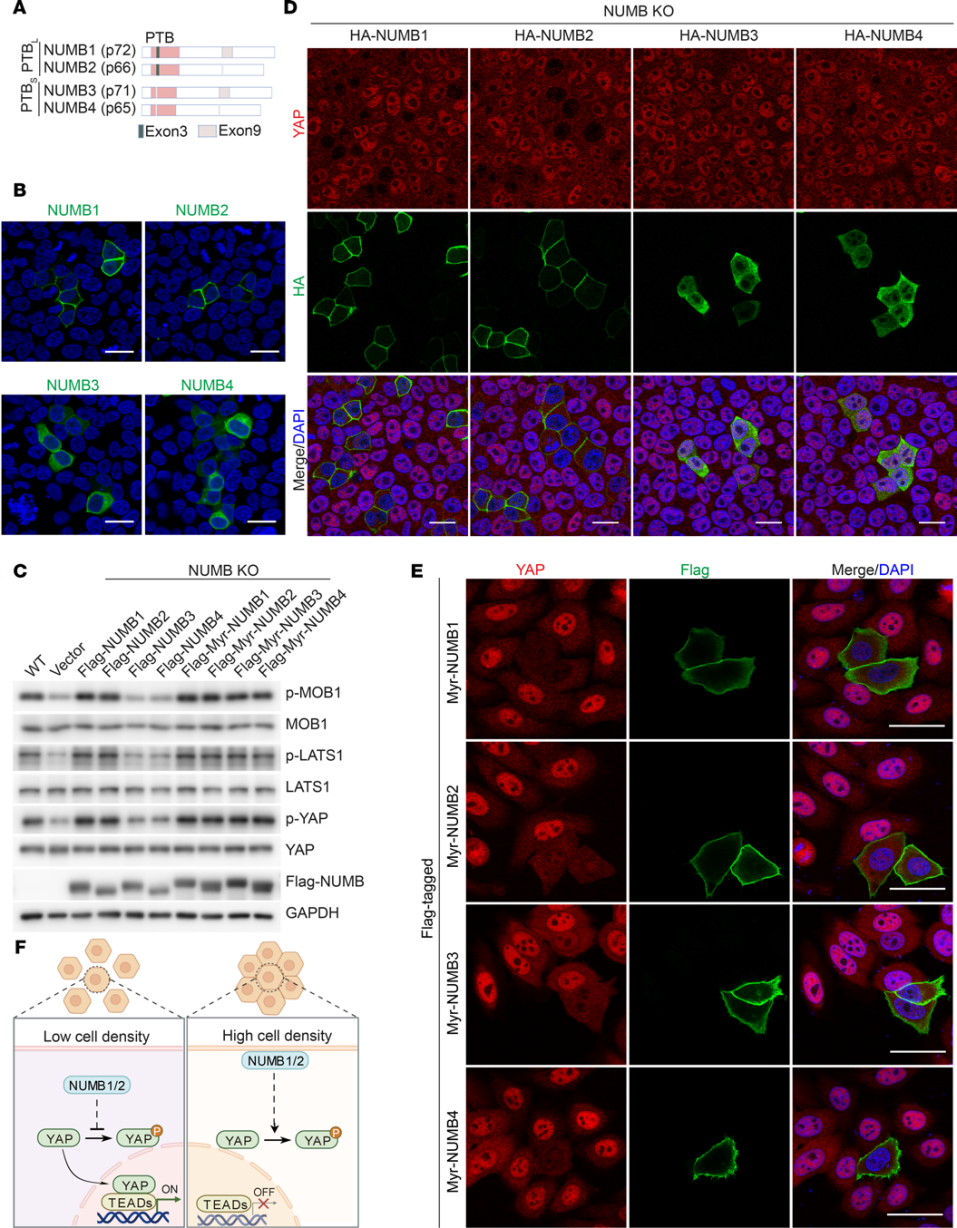
NUMB1/2 alter their subcellular location to modulate YAP activity in response to cell density cues. (**A** and **B**) Diagram of the structure of different NUMB isoforms (**A**) and immunofluorescence staining in HepG2 cells transfected with the indicated constructs (**B**). (**C**) Immunoblot analysis of the indicated proteins in WT and NUMB-KO HepG2 cells expressing the indicated constructs and cultured at HCD. (**D**) Immunofluorescence staining for YAP (red) and HA-tagged NUMB (green) in NUMB-KO HepG2 cells transfected with the indicated constructs and cultured at HCD. (**E**) Immunofluorescence staining for YAP (red) and Flag-tagged Myr-NUMB (green) in HepG2 cells transfected with the indicated constructs and cultured at LCD. (**F**) A proposed working model for how NUMB regulates Hippo/YAP signals at different cell densities. Scale bars: 20 μm (**B**, **D** and **E**).

Contact inhibition of growth is a critical mechanism for proper tissue differentiation and growth as well as tumor suppression. The Hippo/MST1/2 pathway plays a key role in this process (4, 39–41). The HepG2 line of human hepatoma cells are nontumorigenic cells and exhibit an epithelium-like morphology, growing as monolayers (42). Consistently, we found that HepG2 cells were much more sensitive to cell density–dependent growth inhibition when compared with liver tumorigenic SK-HEP-1 cells (Supplemental Figure 1A; supplemental material available online with this article; https://doi.org/10.1172/JCI168888DS1). Higher phosphorylation levels of MOB1 (p-MOB1), LATS1 (p-LATS1), and YAP (p-YAP), and lower levels of nuclear YAP in HepG2 cells grown at HCD were observed as compared with cells at LCD, suggesting that the Hippo/YAP signaling cascade was regulated by cell density in HepG2 cells (Figure 1, A and B). To determine potential cell density sensors that shuttle between the plasma membrane and the cytoplasm to regulate Hippo signaling, we performed data-independent acquisition–based (DIA-based) quantitative mass spectrometry (DIA-MS) analysis of plasma membrane/cytoskeleton and cytoplasm fractions of HepG2 cells cultured under LCD or HCD conditions and identified MST2-interacting proteins from HepG2 cell lysates (Supplemental Figure 1B). The DIA-MS results revealed that 310 proteins in MST2 coprecipitates were increased or decreased in the pellet fraction containing plasma membranes at HCD and, accordingly, were decreased or increased in the supernatant fraction containing cytoplasm at LCD (Supplemental Figure 1B). We then narrowed down the candidates to 45 proteins that were previously reported localize both in the cytoplasm and at the plasma membrane. Among these candidates, some, such as MARK2 and MARK3, are well-known regulators of Hippo signaling (43–45), whereas most of the others have not, to our knowledge, been explored in the context of cell density–dependent growth inhibition. The Hippo pathway senses cell density signals to control tissue growth and tumorigenesis by regulating localization of the transcriptional regulator YAP. YAP is predominantly found in the nucleus at LCD but is more cytosolic at HCD (46). To determine which proteins might affect the subcellular localization of YAP at HCD, we performed a targeted siRNA screening assay by knocking them down individually (Supplemental Figure 2A). The results showed that, among these candidates, knockdown of NUMB had the strongest effect on promoting YAP nuclear translocation and accumulation in cells grown at HCD (Figures 1C and Supplemental Figure 2B). Interestingly, immunoblot analysis showed that NUMB protein levels were increased in plasma membrane fractions (p), and reduced in cytoplasm fractions (c) when cell culture conditions were changed from LCD to HCD (Figure 1D). Immunofluorescence analysis confirmed that NUMB was predominantly located in the cytoplasm and that YAP was located in the nucleus in cells grown at LCD, whereas NUMB was largely localized to the plasma membrane and YAP to the cytoplasm at HCD (Figure 1E). NUMB depletion enhanced cell growth at HCD (Figure 1F). Importantly, at HCD, we observed enhanced Hippo signaling, as evidenced by increased phosphorylation levels of MOB1, LATS1, and YAP, greater cytoplasmic location of YAP, and decreased mRNA levels of the YAP target genes *CTGF* and *CTR61* in WT cells but not in NUMB-deficient cells, whereas, at LCD, the activity of Hippo signaling was comparable between WT cells and NUMB-deficient cells, indicating that NUMB modulated Hippo signaling in response to cell density cues (Figure 1, G–I). Previous studies reported that *NUMB* has 4 major alternatively spliced transcripts (30, 37): two NUMB-PTB_L_ isoforms containing long PTB domains, i.e., NUMB1 (p72) and NUMB2 (p66), and 2 NUMB-PTB_S_ isoforms with short PTB domains, i.e., NUMB3 (p71) and NUMB4 (p65). We observed that NUMB1/2 were mainly located at the cell plasma membrane at HCD, whereas NUMB3/4 were constitutively located in the cytoplasm (Figure 2, A and B). Interestingly, overexpression of NUMB-PTB_L_ (i.e., NUMB1/2), but not NUMB-PTBs isoforms (i.e., NUMB3/4), dramatically enhanced Hippo/YAP signaling, promoted YAP nuclear exit, and inhibited cell proliferation in NUMB-deficient cells (Figure 2, C and D, and Supplemental Figure 3, A–C). Furthermore, reintroduction of myristoylated NUMB3 or NUMB4 (Myr-NUMB3 or 4), which promotes plasma membrane localization of NUMB3 or 4, had the same effect as expressing NUMB1 or 2 to increase the phosphorylation levels of MOB1, LATS1, and YAP in NUMB-deficient HepG2 cells at HCD (Figure 2C). In addition, expression of Myr-NUMB1, 2, 3, or 4 was able to promote YAP exit from the nucleus to the cytoplasm in HepG2 cells grown at LCD (Figure 2E). These results suggested that the subcellular location of NUMB1/2 was altered to regulate Hippo/YAP signaling in response to cell density cues for growth inhibition (Figure 2F).

### HCD induces SPTAN1 phosphorylation for NUMB1/2 membrane retention.

**Figure 3:**
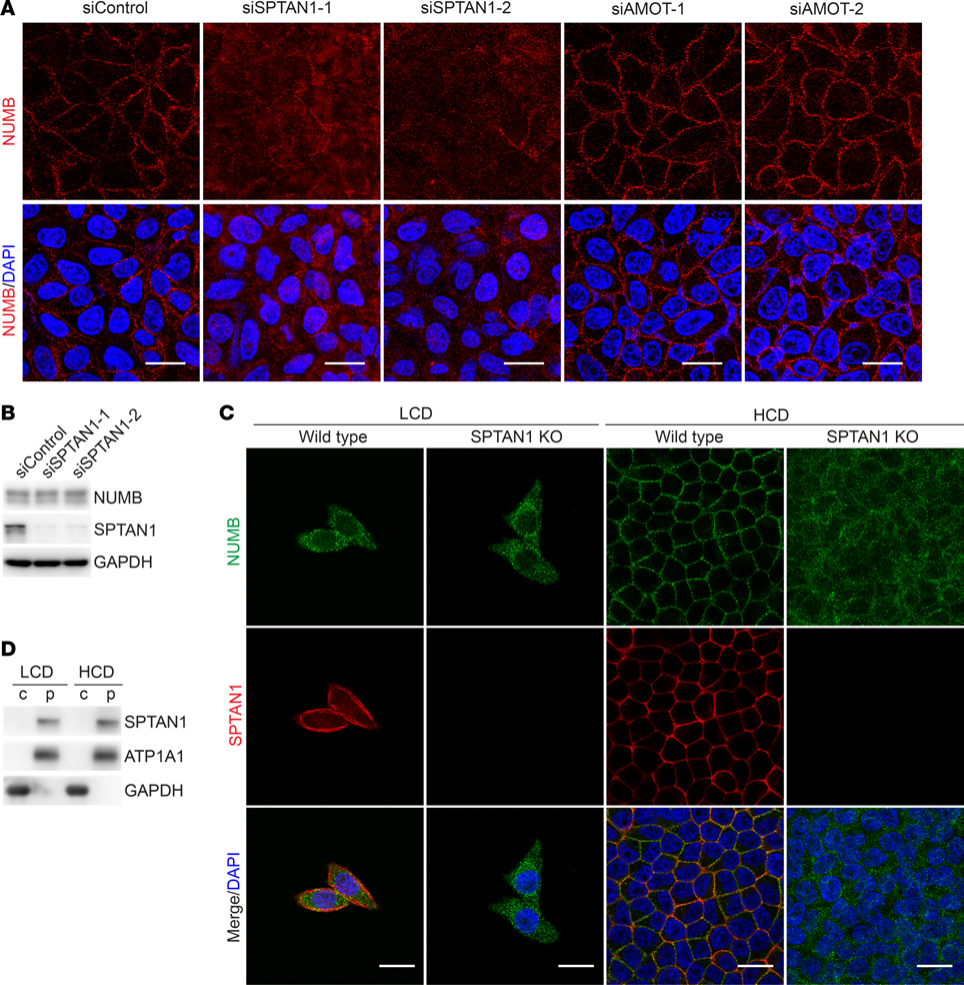
SPTAN1 is required for NUMB1/2 membrane retention at HCD. (**A**) Immunofluorescence staining for NUMB (red) and DAPI (blue) in HepG2 cells transfected with the indicated siRNAs and cultured at HCD. Scale bars: 20 μm. (**B**) Immunoblot analysis of NUMB, SPTAN1, and GAPDH in HepG2 cells transfected with the indicated siRNAs. (**C**) Immunofluorescence staining for NUMB (green) and SPTAN1 (red) in WT and SPTAN1-KO HepG2 cells cultured at LCD or HCD. Scale bars: 20 μm. (**D**) Immunoblot analysis of SPTAN1 in the cytoplasmic fraction and the plasma membrane fraction, isolated from HepG2 cells cultured at LCD or HCD.

**Figure 4:**
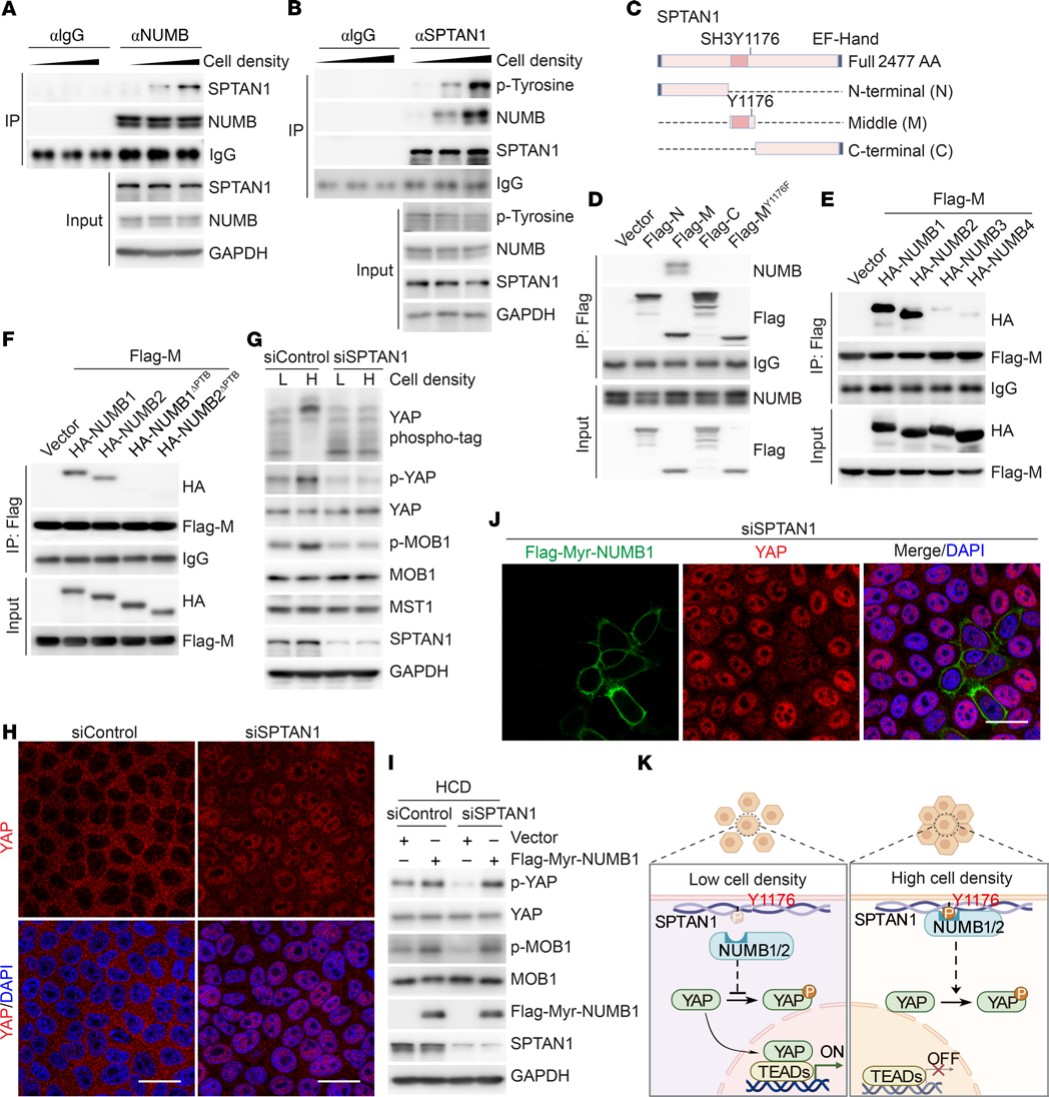
Induced SPTAN1 phosphorylation at HCD for NUMB1/2 membrane retention. (**A** and **B**) Whole-cell lysates from HepG2 cells cultured at different cell densities were collected for co-IP analysis. (**C**) Diagram of the structures of SPTAN1 and Flag-tagged truncated mutants of SPTAN1. SH3, Src homology 3 domain; EF-hand, calcium-binding motif. (**D**–**F**) Immunoblot analysis of lysates from HEK293T cells cotransfected with the indicated constructs, immunoprecipitated with anti-Flag, and analyzed by immunoblotting with the indicated antibodies. (**G**) Phospho-tag and SDS-PAGE analysis of the indicated proteins in HepG2 cells transfected with siControl or siSPTAN1 (the mixture siRNA of siSPTAN1-1 and siSPTAN1-2) and cultured at LCD (L) or HCD (H). (**H**) Immunofluorescence staining for YAP (red) in HepG2 cells transfected with siControl or siSPTAN1 and cultured at HCD. Scale bars: 20 μm. (**I**) Immunoblot analysis of lysates of HepG2 cells expressing Flag-tagged Myr-NUMB1 or control vector transfected with siControl or siSPTAN1 and cultured at HCD. (**J**) Immunofluorescence staining for Flag (green) and YAP (red) in HepG2 cells transfected with siSPTAN1 or Flag-tagged Myr-NUMB1 and cultured at HCD. Scale bar: 20 μm. (**K**) Proposed working model for how the SPTAN1/NUMB axis regulates Hippo/MST/YAP signaling at different cell densities.

The cellular localization of NUMB1/2 regulates Hippo activity. To determine which factors regulate the translocation of NUMB1/2 from the cytoplasm to the plasma membrane during the transition from LCD to HCD, we performed mass spectrometric analysis, which revealed that several proteins involved in cell contact inhibition or cell geometry regulation, including α-catenin, β-catenin, AJUBA, ZO-1, ZO-2, NF2, FRMD6, WWC1, AMOT, and SPTAN1, were present in NUMB coprecipitates (Supplemental Figure 4A). To find out which proteins could alter the subcellular localization of NUMB at HCD, we knocked down these candidates individually using a siRNA (Supplemental Figure 4B). Interestingly, the depletion of SPTAN1, a cell geometry regulator, led to NUMB cytoplasmic retention at HCD without affecting the expression of NUMB (Figure 3, A and B, and Supplemental Figure 4C). Similarly, NUMB was largely located in the cytoplasm of WT cells at LCD, and colocalized with SPTAN1 on the plasma membrane at HCD, while NUMB was mainly found in the cytoplasm in SPTAN1-KO cells regardless of the LCD or HCD condition (Figure 3C). SPTAN1 has been shown to regulate the Hippo pathway (9, 10, 23), although the mechanisms of this regulation remain elusive. Of note, SPTAN1 was exclusively located on the cell membrane regardless of cell density, and its association with NUMB was enhanced as the cell density increased (Figure 3, C and D, and Figure 4A). Since NUMB contains a PTB domain within its N-terminus, we wondered whether the interaction of NUMB and SPTAN1 depends on tyrosine phosphorylation (p-Tyr) of SPTAN1. Indeed, we found that SPTAN1 p-Tyr levels were increased and correlated with enhanced interaction of SPTAN1 and NUMB at HCD (Figure 4B). Phosphorylation of SPTAN1 at tyrosine 1176 (Tyr1176) has been reported to protect SPTAN1 from calpain degradation, which may prevent focal adhesion disruption and mediate signal transmission (47–49). After mapping the interaction regions between SPTAN1 and NUMB, we found that NUMB could bind to the middle region of SPTAN1 containing Y1176 (SPTAN1-M), but not the N- or C-terminus of SPTAN1, and this interaction was abolished when tyrosine 1176 was mutated to phenylalanine (SPTAN1-M Y1176F), mimicking unphosphorylated SPTAN1-M (Figure 4, C and D). Meanwhile, the PTB long isoforms NUMB-PTB_L_ (i.e., NUMB1 and NUMB2), but not the truncated PTB splicing short isoforms NUMB-PTB_S_ (i.e., NUMB3 and NUMB4), were able to associate with the middle region of SPTAN1, and this interaction was abolished when the PTB domain of NUMB1/2 was deleted (Figure 4, E and F). Moreover, we generated the point mutation of Y1176 to F1176 in SPTAN1 in HepG2 cell lines using the CRISPR/Cas9-knockin system and found that the Y1176F mutation of SPTAN1 abolished the interaction between NUMB and SPTAN1 and the retention of NUMB on the cell’s plasma membrane under HCD (Supplemental Figure 5, A and B), suggesting that NUMB translocated from the cytoplasm to the plasma membrane depending on the phosphorylation of SPTAN1 at Y1176. It has been reported that the tyrosine kinase SRC can phosphorylate SPTAN1 at Tyr1176 in vitro (47–49), and SRC kinase activity has been shown to be increased in cells cultured at HCD but not at LCD (50). We also consistently observed that the kinase activity of SRC was increased, as shown by the enhanced phosphorylation levels of Tyr416 on SRC, in HepG2 cells when the cell density increased (Supplemental Figure 6A), and, interestingly, SRC was mostly located on the plasma membrane at HCD and translocated to the cytoplasm (close to the nucleus) at LCD (Supplemental Figure 6B). Thus, we speculated that SRC might be responsible for SPTAN1 phosphorylation at Tyr1176 at HCD. Indeed, either knockdown of SRC or treatment with bosutinib, a SRC family kinase inhibitor, remarkably decreased the tyrosine phosphorylation level of SPTAN1 and reduced the interaction between SPTAN1 and NUMB, as well as the retention of NUMB on the plasma membrane in HepG2 cells cultured at HCD (Supplemental Figure 6, C–F). Moreover, reduced phosphorylation levels of MOB1, LATS1, and YAP and increased nuclear accumulation of YAP were found in SPTAN1 Y1176F-mutant cells at HCD as compared with WT cells (Supplemental Figure 7, A and B). Furthermore, SPTAN1 knockdown dramatically suppressed Hippo signaling, as shown by decreased phosphorylation levels of MOB1, LATS1, and YAP and enhanced nuclear accumulation of YAP at HCD (Figure 4, G and H), which is consistent with previous reports (9, 10). Importantly, these effects could be reversed by the reintroduction of Myr-NUMB1, which was constitutively located at the plasma membrane (Figure 4, I and J). Collectively, these results suggested that SPTAN1-mediated plasma membrane retention of NUMB1/2 was essential for the activation of Hippo signaling (Figure 4K).

### NUMB1/2 sequester MARK in the plasma membrane, resulting in MST1/2 activation.

**Figure 5:**
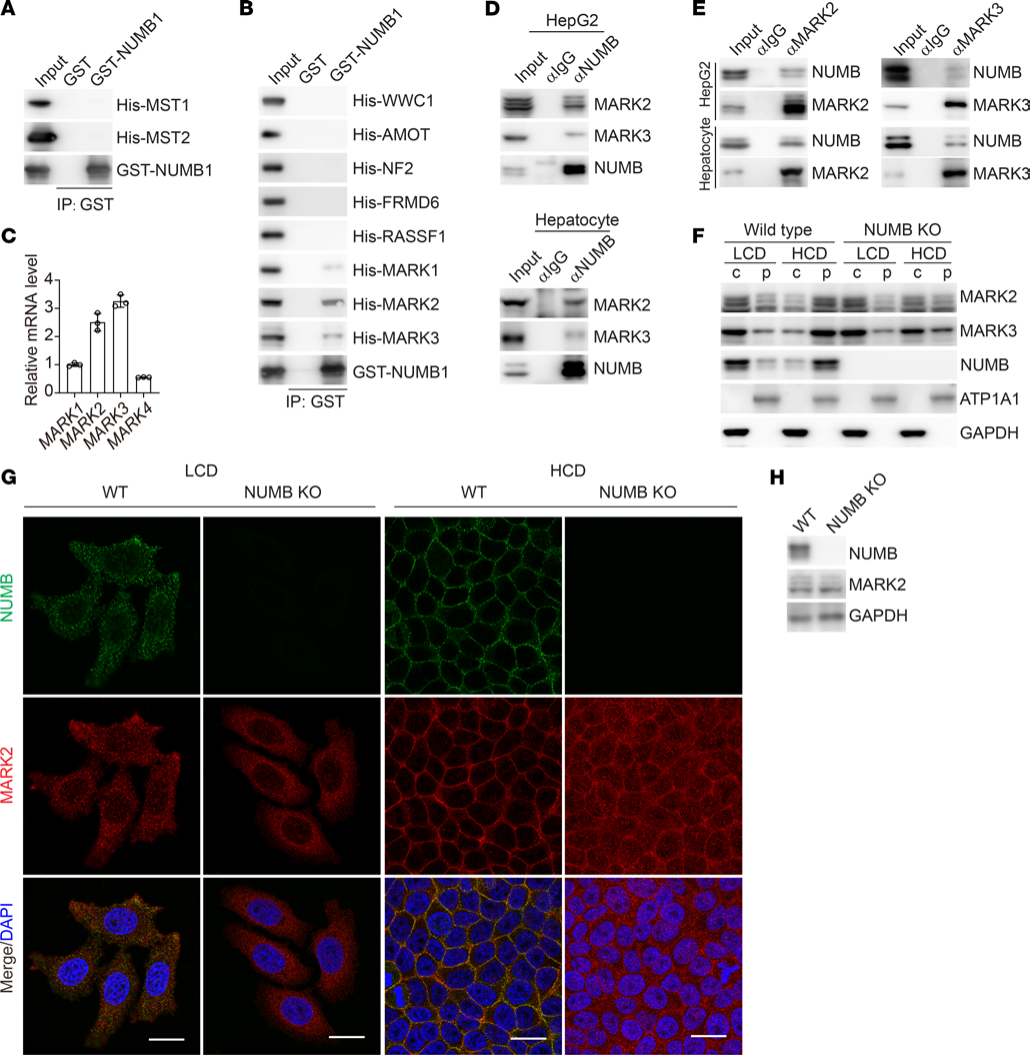
NUMB1/2 sequester MARK on the plasma membrane. (**A** and **B**) Immunoblot analysis of the indicated proteins in GST affinity bead precipitates of GST-tagged NUMB with His-tagged proteins. (**C**) qPCR analysis of the relative mRNA levels of *MARK1*–*4* in HepG2 cells. Each bar represents the mean ± SD from biological triplicate experiments. (**D** and **E**) Whole-cell lysates from HepG2 cells or primary hepatocytes extracted from WT mice were collected for co-IP analysis. (**F**) Immunoblot analysis of the indicated proteins in the cytoplasmic fraction and the plasma membrane fraction isolated from WT and NUMB-KO HepG2 cells cultured at LCD or HCD. (**G**) Immunofluorescence staining for NUMB (green) and MARK2 (red) in WT and NUMB-KO HepG2 cells cultured at LCD or HCD. Scale bars: 20 μm. (**H**) Immunoblot analysis of the indicated proteins in WT and NUMB-KO HepG2 cells.

**Figure 6:**
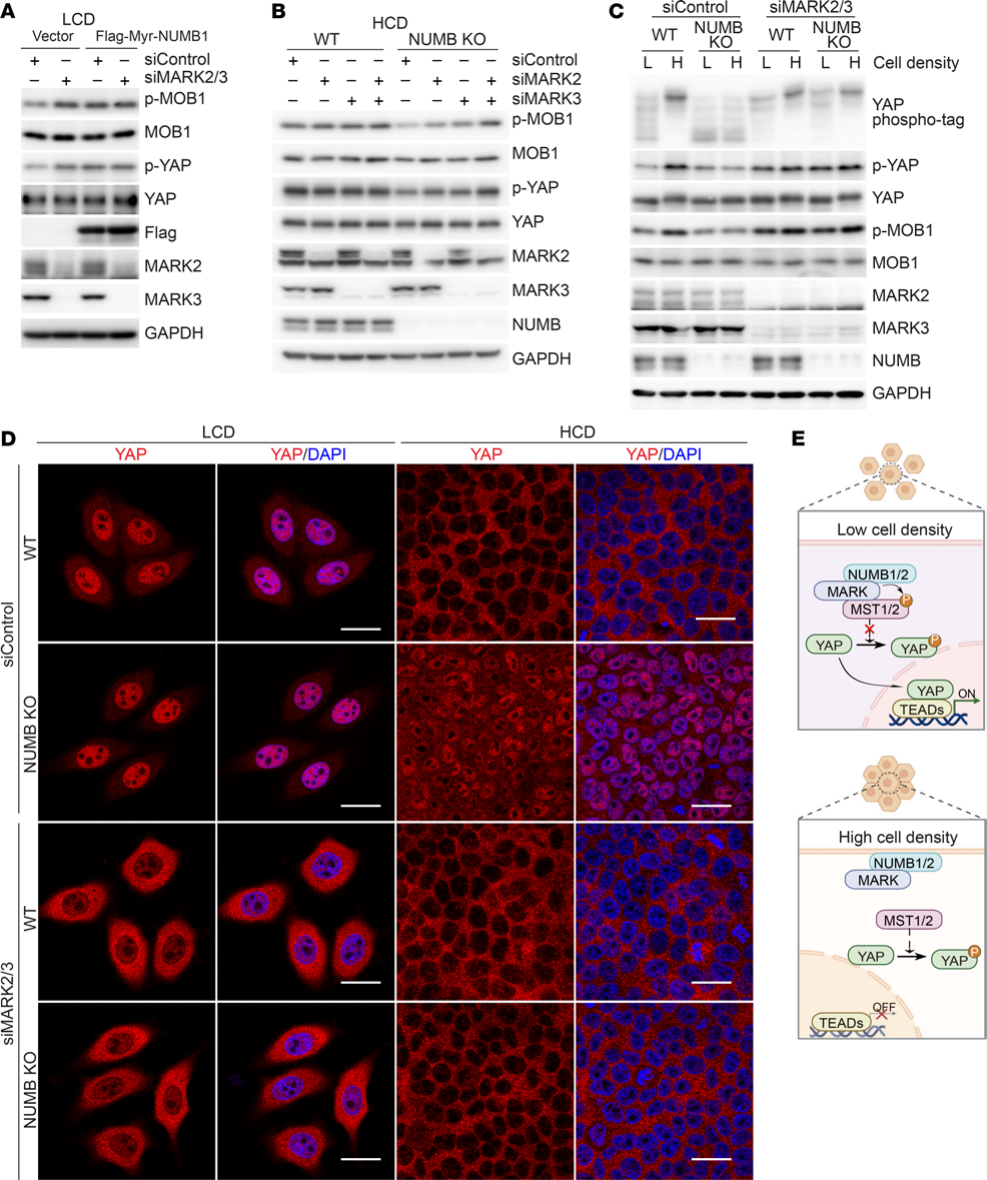
MARK on the plasma membrane leads to MST1/2 activation. (**A**) Immunoblot analysis of the indicated proteins in HepG2 cells expressing Flag-tagged Myr-NUMB1 or control vector cotransfected with siControl or the mixture of siMARK2 and siMARK3 (siMARK2/3) and cultured at LCD. (**B**) Immunoblot analysis of the indicated proteins in WT and NUMB-KO HepG2 cells cotransfected with siControl, siMARK2 (the mixture siRNA of siMARK2-1 and siMARK2-2), siMARK3 (the mixture siRNA of siMARK3-1 and siMARK3-2), or siMARK2/3 and cultured at HCD. (**C** and **D**) Phospho-tag and SDS-PAGE analysis (**C**) or immunofluorescence staining (**D**) of WT and NUMB-KO HepG2 cells cotransfected with siControl or siMARK2/3 and cultured at LCD or HCD. Scale bars: 20 μm. (**E**) Proposed working model of how the NUMB-MARK complex regulates Hippo/MST/YAP signaling at different cell densities.

We next sought to determine how the SPTAN1-NUMB1/2 complex regulates Hippo kinase MST1/2 activity. Although NUMB could be found in coprecipitates of MST2, glutathione S-transferase–pulldown (GST-pulldown) assays showed that NUMB1 did not directly bind to MST1 or MST2 (Figure 5A and Supplemental Figure 8A). Mass spectrometric analysis of NUMB1 coprecipitates revealed several proteins that were previously reported to act as upstream regulators of MST1/2, including WWC1, AMOT, NF2, FRMD6, MARK1, MARK2, MARK3, and RASSF1 (11, 43–46, 51–56). Among these proteins, only MARK1, MARK2, and MARK3, negative regulators of MST1/2 kinases, could directly interact with NUMB1 (Figure 5B and Supplemental Figure 8, B and C). MARK1-4 are mammalian orthologs of the *Drosophila* Par-1 kinase, which has been shown to phosphorylate and repress Hpo kinase activity for organ size control (43–45). Quantitative PCR analyses revealed that, among MARK1-4, MARK2 and MARK3 were highly expressed in HepG2 cells (Figure 5C), and co-IP analysis confirmed the interaction of NUMB with MARK2 or MARK3 in HepG2 cells and mouse hepatocytes (Figure 5, D and E). Similar to NUMB, the protein levels of MARK2 or MARK3 were increased in plasma membrane fractions and reduced in cytoplasm fractions when cell culture conditions were changed from LCD to HCD (Figure 5F). In addition, NUMB deficiency had no effect on the cellular distribution of MARK2/3 at LCD, but dramatically increased cytoplasmic distribution of MARK2/3 and reduced membrane retention when cells were cultured at HCD (Figure 5F), suggesting that recruitment of MARK2/3 to the plasma membrane was largely dependent on NUMB at HCD. With available antibodies, we found that, in line with NUMB1/2, MARK2 was translocated from the cytoplasm to the plasma membrane when cell cultures were changed from LCD to HCD (Figure 5G). In addition, MARK2 was no longer exclusively located at the plasma membrane in NUMB-deficient cells at HCD, indicating that retention of MARK2 at the plasma membrane relied on NUMB (Figure 5, G and H). It has been previously shown that the MARK2 kinase inhibits MST1/2 activity through phosphorylation of MST1/2 at Thr440/Ser444 (56). We found that knockdown of MARK2/3 in HepG2 cells resulted in increased MST1/2 activity, as shown by enhanced phosphorylation levels of MOB1 and YAP and increased YAP nuclear exit at LCD, but this knockdown had no obvious effects on Hippo signaling at HCD (Figure 6, A–D). In contrast, reintroduction of Myr-NUMB1 in MARK2/3-depleted HepG2 cells had no effect on Hippo signaling at LCD (Figure 6A), while knockdown of MARK2/3 restored Hippo activity in NUMB deficient cells grown at HCD (Figure 6, B–D), indicating that the plasma membrane retention of MARK by NUMB was critical to abrogate MARK-mediated inhibition of Hippo signaling. Taken together, these results suggested that cell density modulated Hippo signaling activity by altering the subcellular location of the NUMB-MARK complex (Figure 6E).

### NUMB and WW45 restrain liver dedifferentiation and tumorigenesis via suppression of MARK activity.

**Figure 7:**
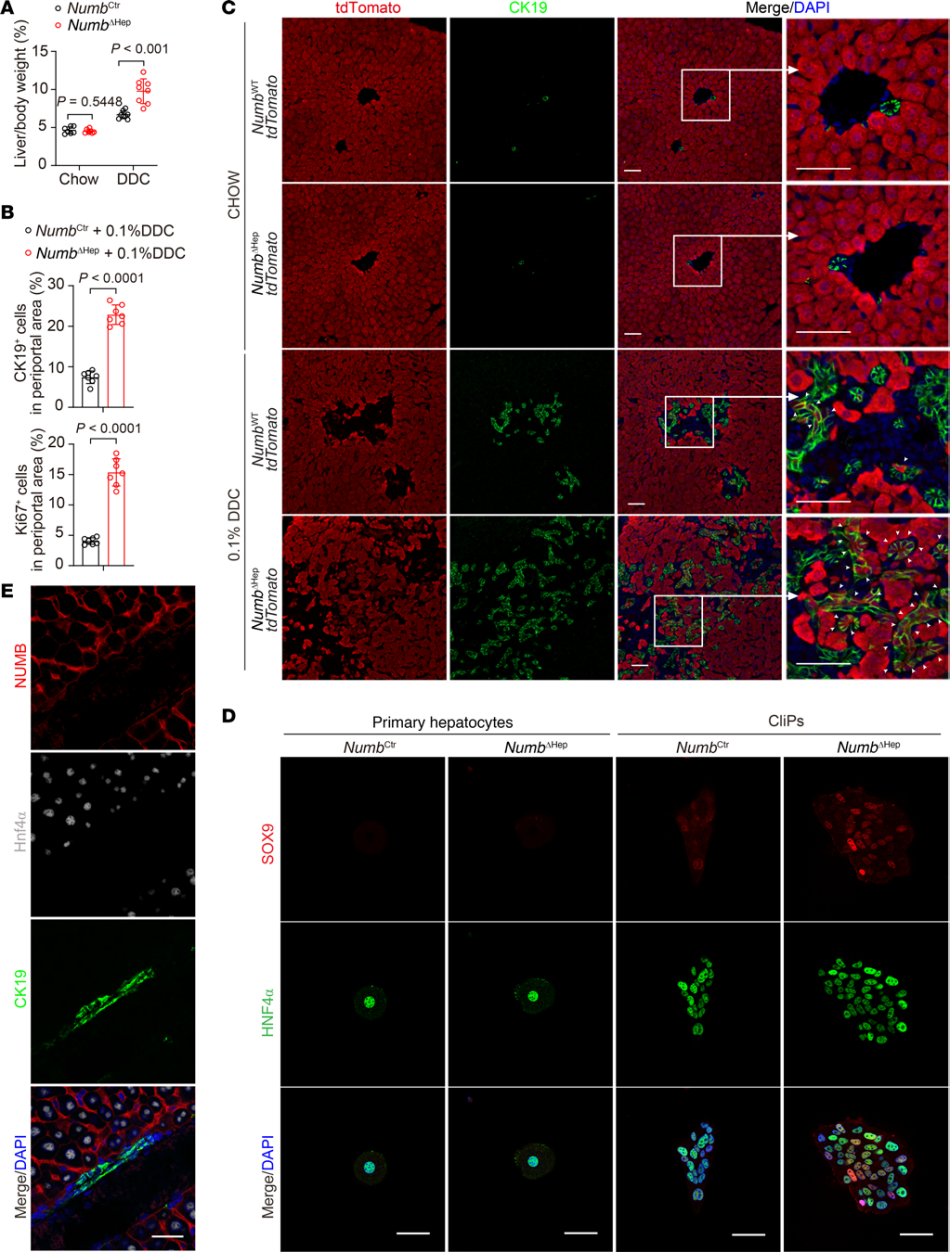
NUMB deficiency promotes hepatocytes dedifferentiation in vivo and in vitro. (**A**) Liver/BW ratios (*n* = 7, 6, 8, 8) of *Numb*^Ctr^ and *Numb*^ΔHep^ mice treated with chow only or chow with DDC. (**B**) Percentage of CK19^+^ or Ki67^+^ cells in the liver periportal areas of *Numb*^Ctr^ and *Numb*^ΔHep^ mice treated with 0.1% DDC. (**C** and **D**) Immunofluorescence staining for the indicated proteins in liver sections from *Numb*^Ctr^ and *Numb*^ΔHep^ (with tdTomato labeled hepatocytes) mice treated with chow or DDC (**C**) or in CLiPs derived from primary hepatocytes of *Numb*^Ctr^ and *Numb*^ΔHep^ mice (**D**). Scale bars (including insets): 50 μm. (**E**) Immunofluorescence staining for the indicated proteins in a WT mouse liver section. Scale bar: 50 μm. Data are presented as the mean ± SD. *P* values were assessed by 2-tailed, unpaired Student’s *t* test (**A** and **B**).

The liver is composed of tightly packed hepatocytes and cholangiocytes, so we wondered whether depletion of NUMB in liver cells would inactivate Hippo signaling and result in loss of growth inhibition to promote liver overgrowth. To this end, we injected *Numb^fl/fl^* mice with adeno-associated virus serotype 8 (AAV8) expressing Cre recombinase under the TBG promoter (AAV-TBG-Cre) or with control viruses to generate hepatocyte-specific NUMB KO (*Numb*^ΔHep^) or WT control (*Numb*^Ctr^) mice, respectively. Surprisingly, we found that KO of NUMB in hepatocytes did not alter the liver size or the liver/BW ratio, nor did it alter the percentages of Ki67^+^ cells and CK19^+^ biliary/progenitor cells in periportal areas of the liver (Supplemental Figure 9, A–C). However, when mice were fed 0.1% 3,5-diethoxycarbonyl-1,4-dihydrocollidine (DDC) to mimic the ductular reaction (DR) observed in human chronic liver diseases (57, 58), *Numb*^ΔHep^ mice exhibited dramatically increased liver/BW ratios, a higher percentage of Ki67^+^ proliferating cells and CK19^+^ cells, decreased phosphorylation levels of MOB1 and YAP, and increased nuclear YAP in the liver compared with *Numb*^Ctr^ control mice (Figure 7, A and B, and Supplemental Figure 9, D–F). It has been reported that CK19^+^ cells during liver injury are not only generated from proliferating biliary cells but are also derived from hepatocyte dedifferentiation (59). To determine the origin of CK19^+^ cells, we set up a hepatocyte lineage–tracing system by injecting AAV8-TBG-Cre into *R26R*^LSL-tdTomato^ or *Numb^fl/fl^* *R26R*^LSL-tdTomato^ mice to induce tdTomato expression in WT (*Numb*^WT^ *tdTomato*) or NUMB-deficient (*Numb*^ΔHep^ *tdTomato*) hepatocytes, respectively, and then treated these mice with the DDC diet. The results showed that *Numb*^ΔHep^ *tdTomato* livers had substantially more tdTomato-expressing CK19^+^ biliary/progenitor cells than did *Numb*^WT^ *tdTomato* livers, indicating that a greater number of NUMB-deficient hepatocytes transdifferentiated into biliary/progenitor cells (Figure 7C). Similarly, hepatocyte dedifferentiation assays confirmed that NUMB-deficient hepatocytes transdifferentiated into more SOX9^+^ biliary/progenitor cells and had enhanced YAP activities compared with WT hepatocytes (Figure 7D and Supplemental Figure 9, G and H). Importantly, NUMB was highly expressed in HNF4α^+^ hepatocytes but was barely detectable in CK19^+^ biliary/progenitor cells (Figure 7E). These results suggested that NUMB restrained hepatocyte dedifferentiation during liver injury.

We next sought to determine whether MARKs are involved in NUMB-mediated Hippo signaling and hepatic cell dedifferentiation during liver injury. Loss of NUMB resulted in increased MARK2 cytoplasmic retention in hepatocytes with or without DDC treatment (Supplemental Figure 10A), however, the association of MARK2 and MST1 was enhanced only in hepatocytes from DDC-treated mice, indicating that some factors regulating the assembly of MARK2-MST1 complexes were modulated in hepatocytes upon DDC treatment (Supplemental Figure 10B). To this end, we analyzed and compared the expression levels of proteins that regulate MST1/2 activity in liver tissues with or without DDC treatment. To our surprise, WW45 was dramatically reduced in DDC-induced dedifferentiating livers compared with nontreated control livers (Supplemental Figure 10C). Consistently, the expression level of WW45 was much lower in chemically induced liver progenitors (CLiPs) than in primary mouse hepatocytes and was also lower in HepG2 cells than in primary human hepatocytes (PHHs) (Supplemental Figure 10, D and E). WW45 has been shown to function as an essential scaffold adaptor and positive regulator of MST1/2 (60–62). Interestingly, although both MST1 and WW45 were predominantly located in the cytoplasm of HepG2 cells regardless of cell density, the interaction of MST1 and WW45 was enhanced at HCD but not at LCD (Supplemental Figure 11, A and B). In contrast, since MARK2 was mainly located in the cytoplasm at LCD and translocated to the plasma membrane at HCD, the association of MARK2 and MST1 was much stronger at LCD than at HCD (Supplemental Figure 11, A and B). Thus, we speculated that MARK2 might compete with WW45 to bind with MST1 at LCD. Indeed, we found that MST1 bound to MARK2 through its SARAH domain, which is also a critical domain for the interaction of MST1 with WW45, and WW45 could compete with MARK2 to interact with and modulate MST1 activity (Supplemental Figure 11, C–G). Consistently, overexpression of WW45 induced comparable phosphorylation levels of MOB1 and YAP in WT and NUMB-deficient HepG2 cells grown at HCD (Supplemental Figure 11H). These results suggested that WW45 could compete with MARK to modulate Hippo kinases MST1/2 activity.

**Figure 8:**
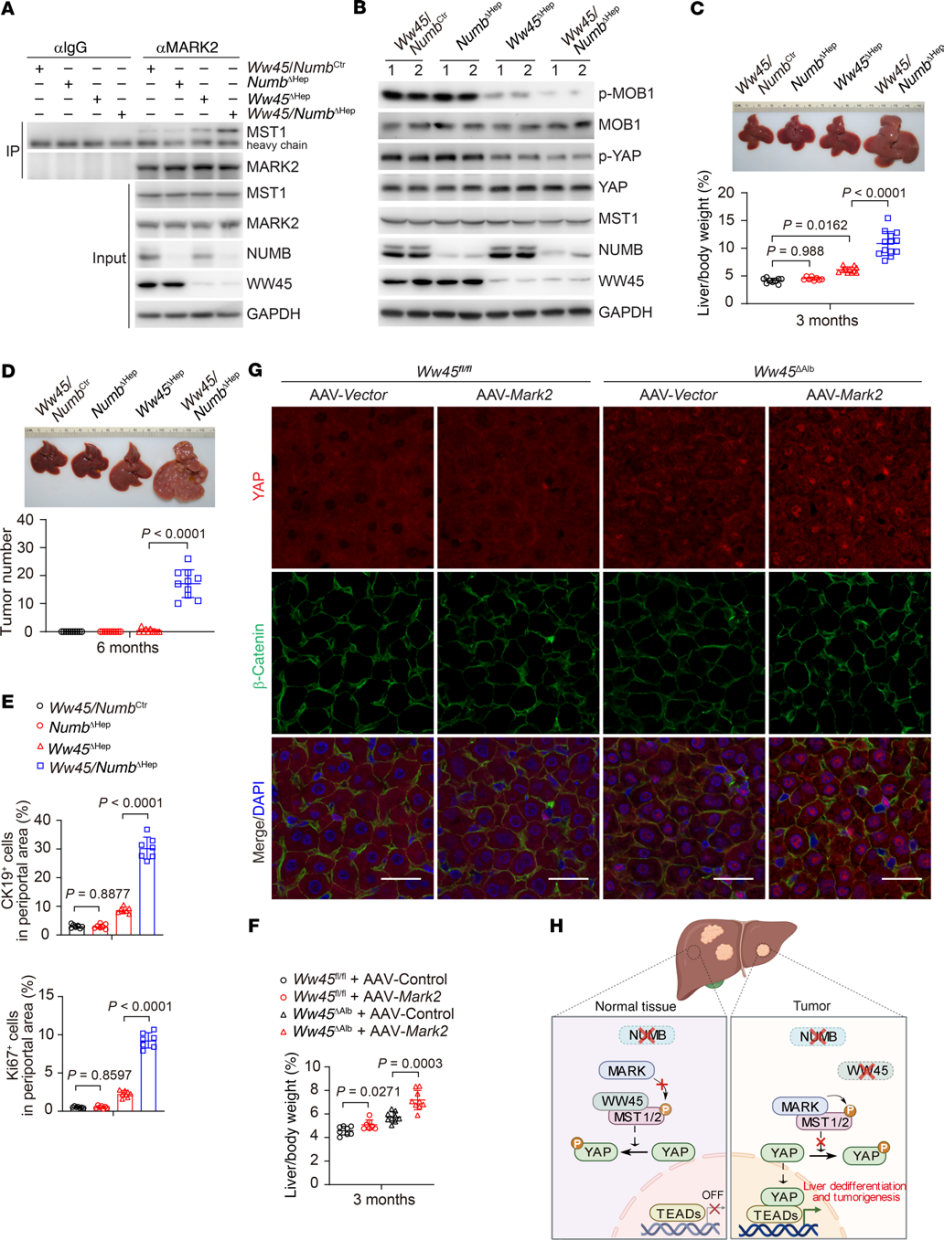
NUMB and WW45 restrain liver dedifferentiation and tumorigenesis via suppression of MARK activity. (**A**) Whole-cell lysates of hepatocytes isolated from mice were collected for co-IP analysis. (**B**–**D**) Immunoblot analysis of the indicated proteins in liver lysates (**B**), representative liver images and liver/BW ratios (*n* = 9, 8, 11, 13) (**C**), and liver tumor numbers (*n* = 10, 10, 10, 10) (**D**) for *Ww45* *Numb*^Ctr^, *Numb*^ΔHep^, *Ww45*^ΔHep^, and *Ww45* *Numb*^ΔHep^ mice. (**E**) Percentage of CK19^+^ or Ki67^+^ cells in the periportal areas of livers from the indicated mice at 3 months of age. (**F** and **G**) Liver/BW ratios (*n* = 8, 7, 9, 9) (**F**) and immunofluorescence staining of liver sections (**G**) from *Ww45^fl/fl^* and *Ww45*^ΔHep^ mice transfected with AAV-Vector or AAV-Mark2. Scale bars: 25 μm. (**H**) Proposed working model of how NUMB-MARK2 tangoing with WW45 mediates MST1/2 activation. Data are presented as the mean ± SD. *P* values were assessed by 1-way ANOVA followed by Tukey’s multiple-comparison test (**C**) and 2-tailed, unpaired Student’s *t* test (**D**–**F**).

We next sought to determine whether WW45 synergizes with NUMB to block MARK function and positively regulate MST1/2 activity in vivo. To this end, we generated *Ww45^fl/fl^* and *Ww45^fl/fl^* *Numb^fl/fl^* mice and injected these mice with AAV-Cre or AAV vector to generate hepatocyte-specific WW45/NUMB double-KO (*Ww45 Numb*^ΔHep^), WW45-KO (*Ww45*^ΔHep^), and control *Ww45* *Numb*^Ctr^ mice, respectively. Compared with hepatocytes from *Ww45 Numb*^Ctr^ and *Numb*^ΔHep^ mice, we observed enhanced interaction of MARK2 and MST1 (Figure 8A), diminished YAP and MOB1 phosphorylation (Figure 8B), more condensed nuclear YAP, and higher expression of *Ctgf* and *Cyr61* (Supplemental Figure 12, A and B) in hepatocytes from *Ww45*^ΔHep^ mice, and these effects were potentiated in *Ww45 Numb*^ΔHep^ hepatocytes, which more closely resemble MST1/2-deficient hepatocytes (63–68). In addition, *Ww45 Numb*^ΔHep^ mice exhibited remarkably increased liver/BW ratios at 3 months of age and greatly accelerated liver tumor formation at 6 months of age (Figure 8, C and D) when compared with *Ww45*^ΔHep^ or *Numb*^ΔHep^ mice. Consistently, *Ww45 Numb*^ΔHep^ livers had much higher percentages of Ki67^+^ and CK19^+^ cells than did their *Ww45*^ΔHep^ and *Numb*^ΔHep^ counterparts (Figure 8E and Supplemental Figure 12C). Similar to *Numb* gene deletion, CRISPR/Cas9-mediated *Sptan1* gene deletion in WT mice did not alter liver size, liver/BW ratios, or liver tumor formation, whereas deletion of *Sptan1* in *Ww45*^ΔHep^ mice remarkably promoted liver enlargement, tumor formation, and YAP activation (Supplemental Figure 13, A–D). Inactivation of 1 allele of *Yap* was sufficient to rescue hepatomegaly and liver cancer development in *Sptan1 Ww45* double-KO mice (Supplemental Figure 13, E and F). Furthermore, overexpression of MARK2 in early postnatal *Ww45*^ΔAlb^ mice with AAV-MARK2 resulted in greatly increased liver/BW ratios and YAP nuclear retention (Figure 8, F and G). These results suggested that NUMB and WW45 synergistically blocked MARK activity to activate MST1/2 and restrain liver overgrowth and tumorigenesis (Figure 8H).

### NUMB and WW45 restrain liver dedifferentiation and tumorigenesis in a YAP- but not NICD/RBP-J–dependent manner.

**Figure 9:**
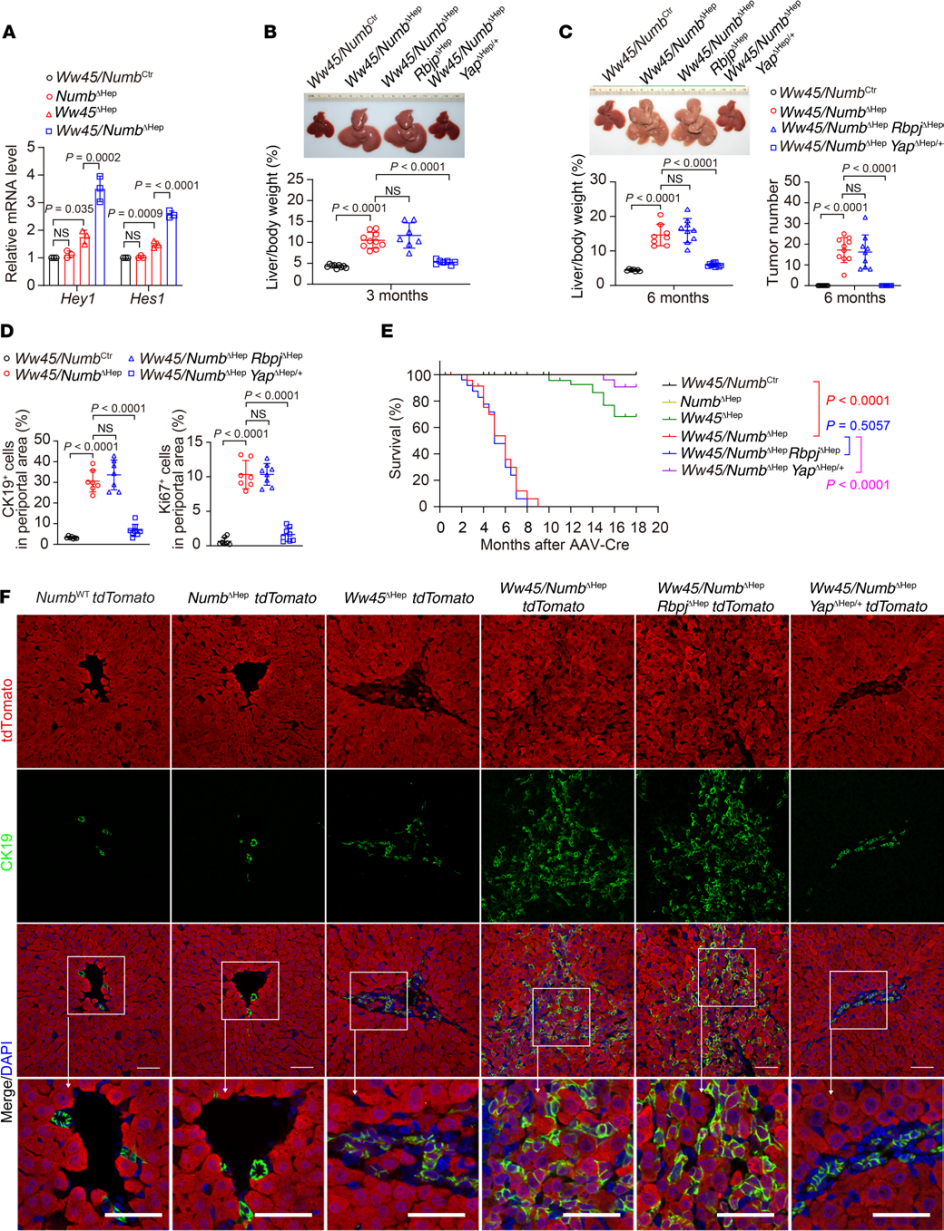
NUMB and WW45 restrain liver dedifferentiation and tumorigenesis in a YAP-dependent manner but not a RBP-J–dependent manner. (**A**) qPCR analysis of *Hey1* and *Hes1* in the livers of *Ww45* *Numb*^Ctr^, *Numb*^ΔHep^, *Ww45*^ΔHep^, and *Ww45* *Numb*^ΔHep^ mice. Each bar represents the mean ± SD from experimental triplicate experiments. (**B**–**D**) Representative liver images and liver/BW ratios of 3-month-old mice (*n* = 9, 10, 7, 7) (**B**) and 6-month-old mice (*n* = 7, 8, 9, 10) (**C**), liver tumor numbers of 6-month-old mice (*n* = 8, 10, 9, 9) (**C**), and the percentage of CK19^+^ or Ki67^+^ cells in liver periportal areas of 3-month-old mice (**D**) of the genotype *Ww45* *Numb*^Ctr^, *Ww45 Numb*^ΔHep^, *Ww45 Numb*^ΔHep^ *Rbpj*^ΔHep^, or *Ww45 Numb*^ΔHep^ *Yap*^ΔHep/+^ as indicated. (**E**) Survival curves (*n* = 18, 17, 25, 25, 29, 23) for *Ww45* *Numb*^Ctr^, *Numb*^ΔHep^, *Ww45*^ΔHep^, and *Ww45* *Numb*^ΔHep^ mice. The indicated *P* values for mortality were determined by Mantel-Cox test. (**F**) Immunofluorescence staining for tdTomato or CK19 in liver sections from *Ww45* *Numb*^Ctr^, *Numb*^ΔHep^, *Ww45*^ΔHep^, and *Ww45 Numb*^ΔHep^ mice with tdTomato-labeled hepatocytes. Scale bars (including insets): 50 μm. Data are presented as the mean ± SD. *P* values were assessed by 1-way ANOVA followed by Tukey’s multiple-comparison test (**A**–**D**).

NUMB is known to be a negative regulator of the Notch/RBPJ signaling pathway and modulates multiple biological processes such as cell fate decision and tumorigenesis (24, 27, 29, 69). We observed that mRNA levels of the YAP target genes *Ctgf* and *Cyr61* and the Notch target genes *Hes1* and *Hey1* were dramatically increased in *Ww45 Numb*^ΔHep^ livers (Figure 9A and Supplemental Figure 12B), indicating that loss of WW45 and NUMB activated both YAP and Notch signaling. To determine the effects of YAP and Notch signals on *Ww45 Numb*^ΔHep^ livers, we further knocked out the *Yap* gene, which encodes the downstream effector of Hippo signaling, or the *Rbpj* gene, which encodes the downstream effector of Notch signaling, in WW45/NUMB-deficient livers by infecting *Ww45^fl/fl^* *Numb^fl/fl^ Yap^fl/+^* or *Ww45^fl/fl^ Numb^fl/fl^ Rbpj^fl/fl^* mice with AAV-Cre to generate *Ww45 Numb*^ΔHep^ *Yap*^ΔHep/+^ or *Ww45 Numb*^ΔHep^ *Rbpj*^ΔHep^ mice (Supplemental Figure 14A). Interestingly, liver overgrowth (Figure 9, B and C), liver tumor formation (Figure 9C), increased Ki67^+^ and CK19^+^ cell percentages (Figure 9D and Supplemental Figure 14B), poor overall survival (Figure 9E), and robust dedifferentiation of hepatocytes (Figure 9F) that resulted from combined WW45 and NUMB deficiency in hepatocytes were all restored to normal levels by deleting 1 allele of *Yap* but not by even full deletion of *Rbpj*. Consistently, more efficient transdifferentiation of NUMB-deficient hepatocytes into CLiPs or SOX9^+^ or CK19^+^ biliary/progenitor cells in NUMB-deficient mice treated with a DDC diet was blocked by 1 allele deletion of *Yap*, but not by even a complete deletion of the *Rbpj* gene (Supplemental Figure 14, C and D). It has been reported that sequential proteolytic cleavages of Notch receptors produce an active Notch intracellular domain (NICD), which is released into the nucleus to participate in target gene transcription (70, 71). To examine whether the liver overgrowth phenotypes of *Numb Ww45*^Δhep^ mice were dependent on NICD, we treated *Numb Ww45*^Δhep^ mice with *N*-[*N*-(3,5-difluorophenacetyl)-l-alanyl]-s-phenylglycine t-butyl ester (DAPT), an inhibitor of the γ-secretase complex that is essential for the generation of NICD (72). The results showed that DAPT treatment did not reduce the liver/BW ratio, the percentage of Ki67^+^ cells,or tumor numbers in *Numb Ww45*^Δhep^ livers, in which protein levels of NICD and mRNA levels of the Notch target genes *Hes1* and *Hey1* were dramatically decreased, while mRNA levels of the YAP target genes *Ctgf* and *Cyr61* were unchanged after DAPT treatment (Supplemental Figure 15, A–F). Collectively, these findings indicated that WW45 and NUMB synergistically restrained liver size and tumorigenesis in a Notch/RBPJ- or Notch/NICD-independent and YAP-dependent manner.

### NUMB3/4 isoforms that fail to activate MST1/2 are dominantly expressed in cancer cells.

**Figure 10:**
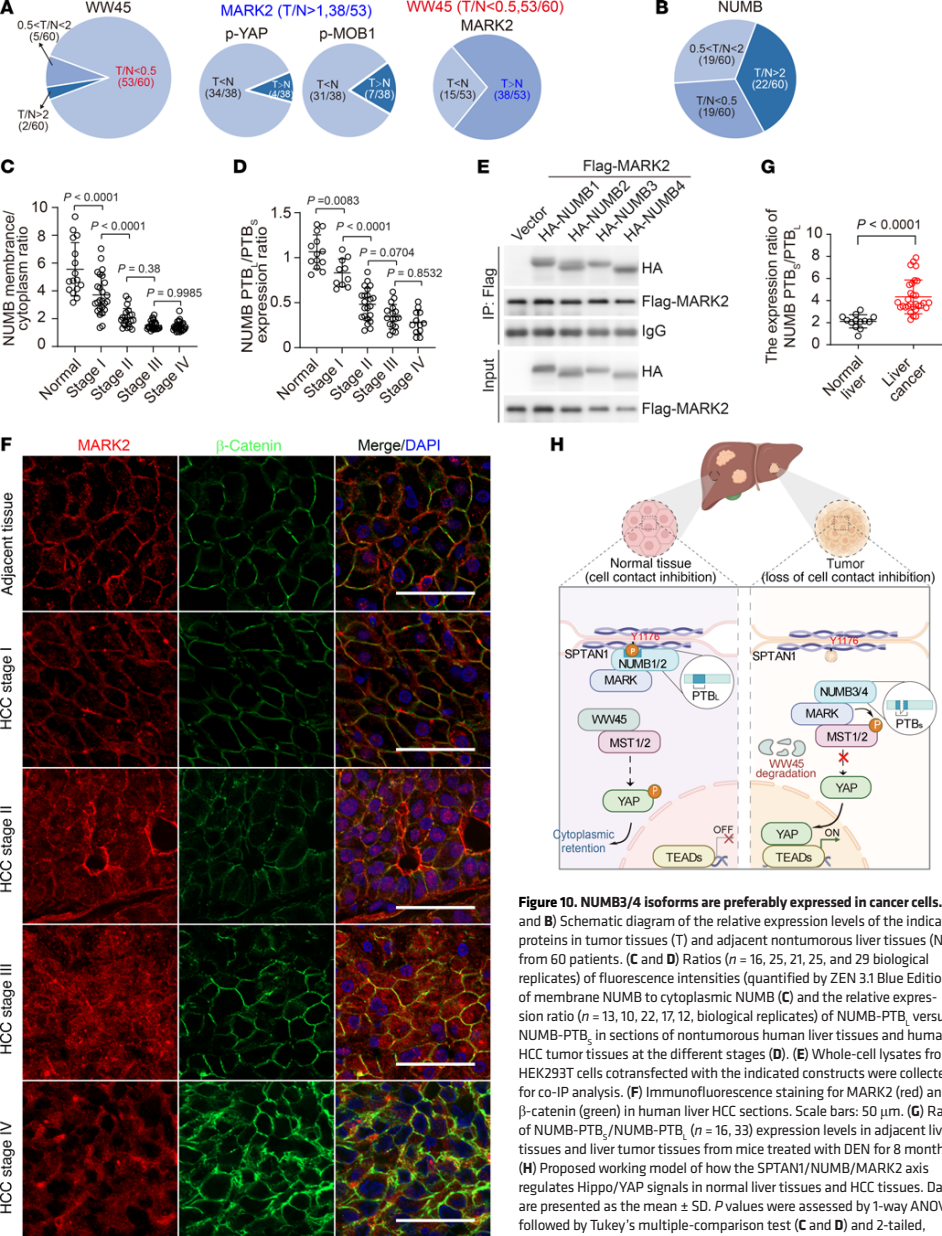
NUMB3/4 isoforms are preferably expressed in cancer cells. (**A** and **B**) Schematic diagram of the relative expression levels of the indicated proteins in tumor tissues (T) and adjacent nontumorous liver tissues (N) from 60 patients. (**C** and **D**) Ratios (*n* = 16, 25, 21, 25, and 29 biological replicates) of fluorescence intensities (quantified by ZEN 3.1 Blue Edition) of membrane NUMB to cytoplasmic NUMB (**C**) and the relative expression ratio (*n* = 13, 10, 22, 17, 12, biological replicates) of NUMB-PTB_L_ versus NUMB-PTB_S_ in sections of nontumorous human liver tissues and human HCC tumor tissues at the different stages (**D**). (**E**) Whole-cell lysates from HEK293T cells cotransfected with the indicated constructs were collected for co-IP analysis. (**F**) Immunofluorescence staining for MARK2 (red) and β-catenin (green) in human liver HCC sections. Scale bars: 50 μm. (**G**) Ratio of NUMB-PTB_S_/NUMB-PTB_L_ (*n* = 16, 33) expression levels in adjacent liver tissues and liver tumor tissues from mice treated with DEN for 8 months. (**H**) Proposed working model of how the SPTAN1/NUMB/MARK2 axis regulates Hippo/YAP signals in normal liver tissues and HCC tissues. Data are presented as the mean ± SD. *P* values were assessed by 1-way ANOVA followed by Tukey’s multiple-comparison test (**C** and **D**) and 2-tailed, unpaired Student’s *t* test (**G**).

We demonstrated that NUMB and WW45 synergistically blocked MARK activity to activate MST1/2 and restrain liver overgrowth and tumorigenesis. To establish the pathological relevance of SPTAN1, NUMB, MARK, WW45, and Hippo/MST1/2/YAP signals in patients with hepatocellular carcinoma (HCC), we examined 60 pairs of liver-derived tumorous and adjacent nontumorous tissues (Supplemental Figure 16A). The relative intensities of immunoblotting bands of SPTAN1, p-Tyr of SPTAN1, NUMB, MARK2, WW45, p-MOB1, and p-YAP in paired samples of adjacent nontumorous tissue (N) and tumorous tissue (T) from the same patient were quantified by ImageJ software, and the ratios of the relative expression levels of these proteins in the T and N samples from each patient were plotted with a heatmap (Supplemental Figure 16B). We found that, although the protein levels of SPTAN1 were comparable in the T and N samples, p-Tyr SPTAN1 was remarkably reduced in a high fraction of tumor samples (Supplemental Figure 16, A and B). Notably, we observed robust reduction of WW45 (i.e., ratios of T/N <0.5; 53 of 60) and dramatically increased MARK2 (i.e., ratios of T/N >2), especially among WW45-low tumor tissues (38 of 53) (Figures 10A and Supplemental Figure 16B). Among the 38 tumor tissues with both upregulation of MARK2 and downregulation of WW45, a high proportion of them had reduced p-YAP (34 of 38) and p-MOB1 (31 of 38) (Figure 10A). However, the variation in NUMB protein levels in tumor tissues, whether higher or lower, was irregular compared with levels in the paired, adjacent nontumorous tissues, as the results showed that 22 of 60 patients had higher expression levels NUMB (ratios of T/N >2) and that 19 of 60 patients had lower expression levels of NUMB (ratios of T/N <0.5) in tumor tissues than levels detected in adjacent nontumorous tissues (Figure 10B). Interestingly, regardless of the high or low expression levels of NUMB, we detected increased cytoplasmic localization of NUMB along with YAP nuclear retention during HCC progression as compared with adjacent normal tissues, in which YAP was distributed in the cytoplasm and NUMB was exclusively located on the plasma membrane (Figures 10C and Supplemental Figure 17A). Considering that NUMB-PTB_L_ isoforms (i.e., NUMB1 and NUMB2) were located on the plasma membrane of cells grown at HCD, while the NUMB-PTB_S_ isoforms (i.e., NUMB3 and NUMB4) were persistently distributed in the cytoplasm (Figure 2B), we sought to determine the relative expression levels of NUMB-PTB_L_ and NUMB-PTB_S_ in healthy human liver versus HCC tissues. Interestingly, quantitative PCR analyses revealed that, compared with NUMB-PTB_L_ isoforms, NUMB-PTB_S_ isoforms that failed to activate Hippo signaling were greatly upregulated along with the progression of HCC (Figure 10D). Since MARK2 could bind to all NUMB isoforms (Figure 10E), we observed that, consistent with the location of NUMB in human HCC tissues (Supplemental Figure 17A), MARK2 was located on the plasma membrane in nontumorous liver tissue and was increasingly present in the cytoplasm during the progression of HCC (Figure 10F). These phenomena were also observed in diethylnitrosamine-induced (DEN-induced) mouse liver cancers, i.e., predominant expression of NUMB-PTB_S_, enhanced cytoplasmic distribution of NUMB and MARK2, and predominant nuclear localization of YAP in DEN-induced mouse liver tumor tissues compared with adjacent normal tissues (Figure 10G and Supplemental Figure 17, B and C). On the basis of these observations, we speculated that high expression of NUMB-PTB_S_ isoforms might disrupt NUMB-mediated activation of Hippo signaling at HCD. To that end, we examined the relative expression levels of NUMB-PTB_S_ and NUMB-PTB_L_ in multiple liver cancer cell lines (Supplemental Figure 18A). Indeed, our results showed that Hippo/MST/YAP signaling could be modulated in HepG2 cells that had relatively higher expression of NUMB-PTB_L_ isoforms, which were able to translocate from the cytoplasm to the plasma membrane and eventually led to nuclear exit of YAP at HCD (Supplemental Figure 18, B and C). In contrast, SNU-423 cells that predominantly expressed NUMB-PTB_S_ isoforms exhibited cytoplasmic distribution of NUMB and high levels of nuclear YAP at HCD (Supplemental Figure 18, B and C). Consistently, reintroduction of membrane-bound Myr-NUMB3 in SNU-423 cells reduced cellular proliferation and colony-forming ability, whereas KO of NUMB in HepG2 cells enhanced cell growth (Supplemental Figure 3B and Supplemental Figure 18, D–G). In addition, overexpression of NUMB1/2 in SNU-423 cells suppressed tumor growth, whereas overexpression of NUMB3/4 increased tumor growth in nude mice (Supplemental Figure 18, H–J). Since NUMB3/4 are distinguished from NUMB1/2 by omitting NUMB exon 6 and ligating exon 5 and exon 7 from NUMB genes, we designed 2 shRNA-targeting sequences that contained the ligation site with flanking regions complementary to both exon 5 and exon 7 to effectively reduce the expression of NUMB3/4 but not NUMB1/2. Our results showed that knockdown of NUMB3/4 substantially suppressed tumor growth of SNU-423 cells in nude mice (Supplemental Figure 18, K–N). Taken together, we concluded that a reduction of p-Tyr in SPTAN1 and NUMB or the favorable expression of NUMB-PTB_S_ isoforms (NUMB3 or NUMB4) in liver might be an important mechanism contributing to the inactivation of Hippo signaling and lead to loss of contact inhibition and liver tumorigenesis (Figure 10H).

## Discussion

Cell density–dependent growth inhibition is crucial to restrain organ growth and tumorigenesis. The Hippo pathway senses cell density information to control tissue growth by regulating localization of the transcriptional regulator YAP/TAZ (4, 41, 73–75). Several upstream signaling complexes, including tight junction complexes, adherens junction complexes, cell polarity complexes, and cytoskeleton complex components such as AMOT, CRB3, LIMD1, F-actin, and hyaluronan, have been shown to regulate YAP activity through multiple mechanisms, directly or indirectly modulating the activation of MST1/2 or LATS1/2 kinases in response to cell density cues (56, 76–78). In this study, we found that the SPTAN1/NUMB1/2/MARK axis acted as an alternative cell density sensor to restrain liver cell growth by coupling cell density–dependent external signals to intracellular Hippo signaling.

Cell junction complexes such as E-cadherin, p120 catenin, and ZO-2 have previously been implicated in cell contact inhibition (11-13, 15, 79–82). In this study, we found that NUMB proteins shuttled between the membrane and the cytoplasm to regulate YAP activity in response to cell density cues. However, knockdown of the components mentioned above in cell junction complexes did not impair NUMB membrane localization. Instead, we found that SPTAN1, the spectrin α chain, was essential for NUMB membrane retention. The spectrin-based membrane cytoskeleton is a major component of the cell cortex, which links the actin cytoskeleton to the plasma membrane (83, 84). In *Drosophila*, spectrins were proposed to regulate Hippo/YAP signaling by binding to Crb, Expanded, Merlin, and Kibra or by modulating cortical actomyosin activity in response to cell density, cell shape, or cortical tension (9, 10, 23). NUMB was shown to be required for sarcomere assembly and maintenance by regulating α-actin filament formation (85). We found that HCD induced SPTAN1 phosphorylation at tyrosine 1176 to recruit NUMB1/2 through their PTB domains and sequestered the MST1/2-inhibitory kinase MARK at the cell membrane. In contrast, at LCD, dephosphorylated SPTAN1 resulted in cytoplasm retention of NUMB1/2 and MARK, which competes with WW45, a positive regulator of MST1/2, to interact with and inhibit MST1/2. Our data suggested that SRC kinase contributed to SPTAN1 phosphorylation at tyrosine 1176 at HCD, but we cannot rule out the possibility that alternative tyrosine kinases may be involved in the process in different tissues. It has been previously reported that SPTAN1 deficiency or mutations in humans cause defects in neural development (86, 87), whereas our studies revealed that in liver, SPTAN1 synergized with WW45 to restrain liver overgrowth and tumorigenesis in a YAP-dependent manner.

Although NUMB family proteins are generally believed to antagonize Notch/RBP-J signaling to influence cell fate (24, 27, 29, 88), we revealed that NUMB1/2 synergized with WW45 to restrain liver differentiation and tumorigenesis in a Notch/RBP-J– or Notch/NICD-independent and YAP-dependent manner. A previous study showed that NUMB was downregulated during human biliary disease and biliary regeneration (89), and we demonstrated that loss of NUMB resulted in enhanced hepatocyte dedifferentiation and promoted the expansion of hepatic progenitors in vivo and in vitro, indicating that NUMB acted as a cell fate determinant in the liver. Interestingly, we found that NUMB deficiency induced hepatocytes dedifferentiation in a YAP-dependent, but not a Notch/RBP-J–dependent, manner and that ablation of NUMB accelerated liver tumorigenesis in WW45-deficient mice through YAP, but not through RBP-J/NICD, suggesting that NUMB family proteins determine liver cell fate through a noncanonical pathway. In addition, mammals express 4 major isoforms of NUMB that differ in the lengths of their PTB domain and proline-rich region (PRR), while *Drosophila* only has 1 isoform. Mammalian NUMB isoforms with different PRR domains have been shown to have distinct roles in cell differentiation and proliferation (30, 90–94), however, the role of NUMB PTB domains was much less appreciated. We revealed that NUMB isoforms with alternatively spliced PTB domains differentially modulated cell fate in responses to cell density cues. Among the 4 major isoforms, NUMB1/2, with long PTB domains, rather than NUMB3/4, with truncated PTB domains, were able to activate Hippo signaling. Importantly, NUMB3/4, which do not activate MST1/2, were selectively upregulated in liver cancer cells, possibly acting as an important oncogenic mechanism contributing to malignant proliferation due to defective contact inhibition. These data might explain the differential activation of MST1/2 upon cell density–dependent growth inhibition in various cancer cell lines. Since NUMB was also shown to function as a tumor suppressor through Gli, p53, and PTEN (33, 34, 95), it is important to determine whether NUMB isoforms with alternatively spliced PTB domains make different contributions to these targets. Thus, the regulatory mechanisms of selective NUMB isoform expression and function in cancer cells remain to be further addressed.

In *Drosophila*, several juxtamenbrane components including adherens or tight junction complexes have been shown to regulate Yorkie (the homolog of mammalian YAP) mainly through Hpo (the homolog of mammalian MST1/2) (41). MST1/2 were reported to be activated in immortalized or cancer cell lines including HEK293 and MCF10A under confluent conditions compared with cells under sparse conditions (76, 96). In this study, we found that different cancer cell lines exhibited different activation levels of MST1/2 at HCD with extensive cell-cell contacts, suggesting a more complicated regulatory mechanism of MST1/2 from the upstream signals induced by HCD-related cell contact in mammals. There are 5 mammalian STE20-like protein kinases (MST) related to the Hippo kinase in *Drosophila*, which includes MST1 MST2, MST3, MST4, and YSK1 (97, 98), so it is of interest to determine whether and how NUMB family proteins regulate other MST kinases. In sum, our study identified the SPTAN1/NUMB1/2/MARK axis as a crucial cell density–sensing regulator for the Hippo pathway kinases MST1/2. SPTAN1 regulates NUMB1/2 cellular location in response to cell density cues. High density causes NUMB1/2 to sequester MARK kinase at the membrane, resulting in activation of the Hippo kinases MST1/2 for cell growth inhibition. Importantly, NUMB3/4 isoforms that fail to interact with SPTAN1 and activate MST1/2 are predominately upregulated in cancer cells. Thus, selective upregulation of NUMB3/4 expression could be an important mechanism to reduce tumor suppressor MST1/2 activity for loss of contact inhibition of growth during the initiation and progression of cancer.

## Methods

For a detailed description of all methods, see the Supplemental Methods.

### Animals.

The *Yap^fl/fl^* and *Ww45^fl/fl^* conditional-KO mice were previously described (65). B6.Cg-Gt(ROSA)26Sor^tm14^ (CAG-tdTomato)Hze/J (stock no. 007914), WT C57BL/6, and Tg (Alb-Cre) 21Mgn/J (stock no. 003574) mice were originally purchased from The Jackson Laboratory. *Rbpi*-floxed mice (catalog NONM-CKO-2101541) were purchased from the Shanghai Model Organisms Center. *Numb^fl/fl^* mice were generated by crossing *Numb^tm1Zili^ Numbl^tm1Zili^/*J mice (Numb/Numblike-floxed, stock no. 005384, The Jackson Laboratory) with WT C57BL/6 mice. Athymic mice (BALB/c) were purchased from Charles River Laboratories.

### Mouse models.

For AAV infection, 4-week-old male or female mice received a tail vein injection of 1 × 10^11^ genome copies of AAV. Specially, further treatments were administered at least 2 weeks after AAV-TBG-Cre injection. To induce chronic liver injury, mice were fed a diet supplemented with 0.1% DDC (MilliporeSigma, 137030) for 4 weeks or another indicated duration. For DAPT treatment, mice were administered DAPT (125 mg/kg in mixed solvent of 90% corn oil/10% DMSO, Aladdin, D126677) or an equivalent volume of vehicle by intraperitoneal injection every other day for 6 or 12 weeks. To induce liver cancer, 2-week-old male C57BL/6 mice were treated with DEN (25 mg/kg in 0.9% saline, MilliporeSigma) by intraperitoneal injection and were sacrificed at 8–9 months of age for liver analysis.

### Statistics.

All statistical analyses were performed using GraphPad Prism 8 (GraphPad Software). The data are presented as the mean ± SD as indicated, and a 2-tailed, unpaired Student’s *t* test was used for comparisons between 2 groups. Survival data were analyzed using the Kaplan-Meier statistical method. The exact number of biological or experimental replicate numbers can be found in the figure legends, and the exact *P* values are indicated in the graphed data. A *P* value of less than 0.05 was considered statistically significant. Most experiments were carried out at least 3 times, and the findings of all key experiments were reliably reproduced.

### Study approval.

All mice were maintained under specific pathogen–free (SPF) conditions at the Xiamen University Laboratory Animal Center. All animal experiments were approved by the IACUC of Xiamen University (Xiamen, Fujian, China) and were in strict accordance with good animal practices as defined by the Xiamen University Laboratory Animal Center. The use of human HCC samples and the relevant database were approved by the research ethics committee of Xiang’an hospital of Xiamen University and Zhongshan Hospital of Xiamen University and complied with all relevant ethics regulations. All tissue samples were collected in compliance with the informed consent policy.

### Data availability.

All data generated or analyzed during this study are included in this manuscript (and its supplemental information files). Values for all data points found in graphs can be found in the Supplemental Supporting Data Values file.

## Author contributions

DZ and LC conceived the project with the input from WH, BZ, DS, YL and WZ. DS, YL, WZ, HG, YC, YH, Junhong LiL, YY, Z Lai, Z Li, HH, Jiaxin Li, Jinhuan Li, MC, CN, NW, ZZ, YX, YZ, HL, JT, QC, and LH performed experimental biological research. PL, WL, and ZP provided human HCC samples. RLJ provided mutant mice. DZ, LC, and DS co-wrote the manuscript, and all authors reviewed and revised the manuscript. The authorship order was assigned on the basis of relative contributions to the study, with the persons most involved in the research listed first and the most experienced contributors last. The authorship order of the co–first authors is based on the relative contributions of their input to the final version of the manuscript.

## Supplementary Material

Supplemental data

Supporting data values

## Version 1 10/16/2023

Electronic publication
