# Supplementary material for: SPTAN1/NUMB axis senses cell density to restrain cell growth and oncogenesis through Hippo signaling
Source: J Clin Invest. 2023 Oct 16;133(20):e168888. doi: 10.1172/JCI168888 (PMC10575737; doi:10.1172/JCI168888)
Supplement: Supplemental data [file jci-133-168888-s047.pdf]

SUPPLEMENTARY METHODS

Tumor Xenografts

The 6- to 8-week-old male athymic mice were injected subcutaneously with 1×10^7 HepG2 cells or 2×10^6 SNU-423 cells in 100 µl of PBS and Matrigel matrix. Six animals per group were used in each experiment. The mice were euthanized 4-weeks after transplantation.

Cell culture

The HEK293T, HepG2, Hep3B, SNU-423, SNU-475, SK-HEP-1 and Sf9 cell lines were purchased from the American Type Culture Collection (Manassas, VA), and MHCC97H cells were obtained from the Chinese Academy of Sciences Cell Bank (Shanghai, China). Primary human hepatocytes (PHH) cells were isolated from fragments of donor liver surplus to surgical requirement. Cells were cultured under the manufacturer's instructions. All of the cells were authenticated by examination of morphology and growth characteristics and confirmed to be mycoplasma free.

Generation of *NUMB* or *SPTAN1* knockout cell lines

NUMB or *SPTAN1* knockout HepG2 cells were generated by transfection of plasmid pX459-sgNUMB or sgSPTAN1, which expresses Cas9 and a small guide RNA against NUMB (TTGATTCAATCAACTTCTACA) or against SPTAN1（GCAGAGCCTGAACACTTGCC）. Forty-eight hours after transfection, cells were selected by puromycin (1 µg/ml) for 2 days, and then were sorted onto 96-well plates for screening one single clone.

Generation of SPTAN1 Y1176F mutation cells

Two 20 bp sgRNAs (Y1176F gRNA-1: ctctccttgtaggaagtgTA; Y1176F gRNA-2:

catgccATAcacttcctaca) were chosen and the cutting sites were about 3 and 8 bp before the mutation site, respectively. The guiding RNA oligonucleotides were inserted into the gRNA cloning vector pSpCas9(BB)-2A-GFP (PX458) (Addgene: #48138). The 1600 bp ssDoner DNA with point mutation were synthesized and amplified through PCR. Cells were transfected with plasmid pX458-Y1176F gRNA and linearized ssDoner DNA. GFP-positive cells were harvested and subjected to the flow cytometry for single-cell sorting. The single cell was cultured in a 96-well plate for about 10 days. Part of colonies were used to identify the genotype. The cells used for genotyping were lysed in the lysis buffer (50 mM KCl, 1.5 mM $MgCl_2$, 10 mM Tris, pH 8.0, 0.5% Nonidet P-40, 0.5% Tween 20, 100 μg/ml protease K) at 65°C for 30 min, then 98°C for 3 min. 1 μl lysate was used as the template to amplify the target sequences using the related primers. Colonies with desired mutation were used for expansion.

Lentivirus production and infection

The pLenti vectors encoding NUMB1, 2, 3, or 4, Myr-NUMB1, 2, 3 or 4 or WW45, as well as three assistant vectors: pMDLg/pRRE, pRSV-REV, and pVSVG, were transiently transfected into HEK293T cells. Viral supernatants were collected 48 hours later, clarified by filtration, and concentrated by ultracentrifugation. Cells were infected with lentivirus carrying vector or indicated constructs. After 48 h, the cells were positively selected by puromycin (1ug/ml), and were then subjected for sorting.

Isolation of Hepatocytes

Mouse primary hepatocytes were isolated as previously described (1). Briefly, the mouse was anesthetized with intraperitoneal injection of pentobarbital sodium (10 mg/kg body weight). The

abdomen was then cut open, and the portal vein was catheterized. The liver was first perfused in situ with D-Hank's buffered solution (containing 0.5 mM EGTA, pre-warmed to 37°C) for 8-10 min with the inferior vena cava cut for drainage and then perfused for 5 min with 0.12 PZ-U/ml collagenase perfusate (containing 2 mM Ca^{2+}). The liver was dissociated, and the cell suspension was collected and filtered through 40 μm filter, resuspended in Percoll/DMEM/PBS (1:1:0.3) mixture and centrifuged at 50 g for 15 min. Cell viability was examined by the Trypan blue exclusion test.

Generation of chemically induced liver progenitors (CLiPs)

Primary mouse hepatocytes were used for induction of CLiPs using protocol as described previously (2). Briefly, freshly isolated hepatocytes were seeded at approximately 1 x 10^4 cells/cm^2 in YAC medium (DMEM/F12 containing 2.4 g/L $NaHCO_3$ and glutamine, which was supplemented with 5 mM HEPES, 30 mg/L l-proline, 0.05% BSA,10 ng/ml EGF, insulin-transferrin-serine (ITS)-X, 10^{-7} M dexamethasone,10 mM nicotinamide,1 mM ascorbic acid-2 phosphate, and an antibiotic/antimycotic solution, 10 μM Y-27632, 1 μM, 0.5 μM A-83-01, and 3 μM CHIR99021) on plates or glass slides coated with collagen type I. The medium was changed one day after seeding and every other day.

Plasmids

Full-length cDNA used in this study were obtained either by amplifying specific cDNA from Hela cells, or by purchasing from Youbio (Hunan, China). His-tagged MARK2/MARK3 in pFastBac-His-C vector and Flag-tagged SPTAN1 in pLenti-puro vector were purchased from Youbio (Hunan, China). Expression plasmids for various epitope-tagged proteins were constructed in the pCMV

vector or pLenti vector for transfection (ectopically expressed in mammalian cells), in the pAAV or pscAAV vector for Adeno-associated viruses packaging, or in the pFastBac-His-C and pGEX-4T-1 vector for protein purification. All plasmids were purified using the CsCl density gradient ultracentrifugation.

Plasma membrane/cytoskeleton and cytosol fractions separation

HepG2 cells grown at low or high cell density were cross-linked with 0.1% formaldehyde for 10 min at 37 °C. According to the instructions, cytosol and plasma membrane/cytoskeleton fractions were extracted with Minute™ Plasma Membrane Protein Isolation and Cell Fractionation Kit (Invent Biotechnologies, SM-005), followed by immunoblotting or DIA-MS analysis.

Data-independent acquisition (DIA) mass spectrometry

Protein samples dissolved in 8M Urea were subjected to trypsin digestion and dried. Samples were analyzed on a nanoElute (Bruker) coupled to a timsTOF Pro (Bruker) equipped with a CaptiveSpray source. Peptides were dissolved in 10 µl 0.1% formic acid and were auto-sampled directly onto a homemade C18 column (35cm X 75µm i.d., 1.9µm 100Å). Samples were then eluted for 60 mins with linear gradients of 3–35% acetonitrile in 0.1% formic acid at a flow rate of 300 nl/min. Mass spectra data were acquired with a timsTOF Pro mass spectrometer (Bruker) operated in DIA-PASEF mode. The raw files were analyzed by DIA-NN software against uniprot database. For the analysis of DIA-MS, the protein component of cytoplasmic extract was normalized with the intensity of GAPDH, a cytoplasmic localized protein, and membrane/cytoskeleton and related-bound protein extract was normalized with the intensity of ATP1A1, a plasma membrane-localized protein. Two biological replicate experiments were performed, and detailed results are shown in

Supplementary Table 1.

SDS-PAGE and immunoblot analysis

As described in our previous study (3), the proteins were separated by SDS-PAGE electrophoresis, transferred to a PVDF membrane and then analyzed by immunoblotting with the appropriate antibodies. The primary antibodies, anti-phosphorylated (p-) MOB1 at Thr35 (1:2,000; 8699), anti-MOB1 (1:2,000; 13730), anti-GAPDH (1:5,000; 5174), anti-NUMB (1:3,000; 2756), anti-LATS1 (1:2,000; 3477), anti-p-LATS1 at Thr1079 (1:2,000; 8654), anti-YAP (1:2000; 14074), anti-p-YAP at Ser 127 (1:3,000; 4911), anti-Flag (1:3,000; 14793), anti-HA (1:3,000, 3724) and anti-RBPJ (1:2,000, 5313), anti-WW45 (1:2,000, 13301), anti-PP2A A (1:3000, 2041), anti-PP2A B (1:3000, 2290), anti-PP2A C (1:3000, 2259), anti-aPKC (PKCζ, 1:2000, 9372), anti-p-MST1 (Thr183)/MST2 (Thr180) (1:2,000, 3681), anti-p-Src (Tyr416) (1:2,000, 6943) and anti-MARK3 (1:2,000, 9311) were from Cell Signaling Technology. Anti-MST1 (1:2,000; 22245-1-AP) and anti-GST (1:4,000, 66001-2-Ig) were from Proteintech. Anti-Hey1 (1:2,000, PA5-40553) was from Invitrogen. Anti-Hes1 (1:1,000, sc-166410), anti-p-Tyrosine (1:500, sc-5267), anti-RASSF1 (1:1,000, sc-58470), anti-SRC (1:1,000, sc-32789) and anti-MYC (1:1,000, sc-40) were from Santa Cruz. Anti-MARK2 (1:1,000, H00002011-M01) was from Novus Biologicals. anti-His-tag (1:5,000, ab18184) was purchased from Abcam. Anti-SPTAN1 (1:2,000, PA5-87363) and anti-Hey1 (1:2,000, PA5-40553) were purchased from Thermo Fisher. Anti-FRMD6 (1:3000, A9995) was purchased from ABclonal. Horseradish peroxidase–conjugated antibody (1:3,000) was purchased from Jackson ImmunoResearch Laboratories. The protein bands were detected and visualized with NcmECL Ultra according to the product instruction (New Cell & Molecular

Biotech).

Immunoprecipitation

Cell lysates were prepared by incubating the cells in NETN buffer (50 mM Tris-HCl, pH 8.0, 150 mM NaCl, 0.2% Nonidet P-40, 2 mM EDTA) in the presence of protease inhibitor Cocktails (Roche) and/or phosphatase inhibitor Cocktails (Roche) for 20 min at 4 ºC. This was followed by centrifugation at 14,000 g for 15 min at 4 ºC. For immunoprecipitation, about 500 μg of protein was incubated with rabbit control IgG (ABclonal, AC005), mouse control IgG (Abclonal, AC011), anti-MARK2 (Biorbyt: orb256676), anti-MARK3 (CST: #9311), anti-NUMB (CST: #2756), anti-SPTAN1 (Sigma: MAB1622), anti-Flag (CST: #14793), or anti-HA (CST: #3724) antibodies (1-2 μg) for overnight at 4 ºC with constant rotation; 50 μl of 50% protein G magnetic beads (Invitrogen) was then added and the incubation was continued for an additional 2 hours. Beads were then washed five times using the lysis buffer. Between washes, the beads were collected by magnetic stand (Invitrogen) at 4 ºC. The precipitated proteins were eluted from the beads by re-suspending the beads in 2 × SDS-PAGE loading buffer and boiling for 5 min. The boiled immune complexes were subjected to SDS-PAGE followed by immunoblotting with appropriate antibodies.

Immunopurification and silver staining

Lysates from HepG2 cells expressing Flag-tagged MST2 or NUMB were prepared by incubating the cells in lysis buffer containing protease inhibitor cocktail (Roche). Anti-Flag immunoaffinity columns were prepared using anti-Flag M2 affinity gel (Sigma) following the manufacturer's suggestions. Cell lysates were obtained from about 5×10^7 cells and applied to an equilibrated Flag column of 1 ml bed volume to allow for adsorption of the protein complex to the column resin.

After binding, the column was washed with cold PBS plus 0.2% Nonidet P-40. Flag peptide (Sigma) was applied to the column to elute the Flag protein complex as described by the vendor. The elutes were collected and visualized on NuPAGE 4-12% Bis-Tris gel (Invitrogen) followed by silver staining with silver staining kit (Pierce). The distinct protein bands were retrieved and analyzed by LC-MS/MS.

Mass spectrometry

After staining of gels with silver staining, excised gel segments were subjected to in-gel trypsin digestion and dried. Samples were analyzed on a nanoElute (Bruker) coupled to a timsTOF Pro (Bruker) equipped with a CaptiveSpray source. Peptides were dissolved in 10 μl 0.1% formic acid and were auto-sampled directly onto a homemade C18 column (35cm X 75μm i.d., 1.9μm 100Å). Samples were then eluted for 60 mins with linear gradients of 3–35% acetonitrile in 0.1% formic acid at a flow rate of 300 nl/min. Mass spectra data were acquired with a timsTOF Pro mass spectrometer (Bruker) operated in PASEF mode. The raw files were analyzed by Peaks Studio X software against uniprot database. Mass spectrometry analysis of MST2- and NUMB- containing protein complex in HepG2 cells are shown in Supplementary Table 2-3.

RNA interference

All siRNA transfections were performed using Lipofectamine RNAi MAX (Invitrogen) following the manufacturer's recommendations. The final concentration of the siRNA molecules is 10 nM and cells were harvested 48 or 72 hours later according to the purposes of the experiments. The indicated siRNA was the mixture of the individual siRNAs against corresponding gene. The siRNA sequences are available in Supplementary Table 4.

GST Pull-down assays

Recombinant baculovirus carrying His-fusion proteins were generated with the Bac-to-Bac System (Invitrogen). Infected Sf9 cells were grown in spinner culture for 48 to 96 hours at 27 ºC and His-tagged protein purified using Ni^{2+}-NTA agarose (Invitrogen) according to standard procedures. GST-fusion proteins were purified from *Escherichia coli* by glutathione-Sepharose 4B beads (GE Health Care) and then washed with high salt buffer (20 mM Tris-HCl (pH 7.4), 0.1 mM EDTA, and 300 mM NaCl). For GST pull-down assay, GST-fusion proteins were incubated with His-tagged proteins at 4°C overnight. The beads were washed 3 times, then boiled in SDS loading buffer and subjected to SDS-PAGE followed by immunoblotting.

Immunohistochemistry

As described in our previous study (3), the tissue specimens were fixed for 24 h in 4% PFA and then embedded in paraffin for sectioning. Standard staining with hematoxylin and eosin was performed on 5 μm sections. For immunohistochemistry, liver sections were rehydrated and incubated in 3% H_2O_2 for 30 min, boiled with Tris-EDTA buffer (pH 9.0) for antigen retrieval and then blocked with 5% horse serum followed by incubating overnight at 4°C with the primary antibodies including anti-Ki67 (1:500, invitrogen, 14-5698-82) and anti-CK19 (1:10, DHSB, Troma-III). After washing with PBS, tissue sections were incubated with biotinylated anti-rat IgG (1:200, Vector Laboratories) at RT for 30 min and then followed with streptavidin–horseradish peroxidase conjugates (Vector Laboratories) incubation for 30 min. Subsequently, the slides were performed with 3, 3'-diaminobenzidine staining (Vector Laboratories) and then counterstained with hematoxylin.

Immunofluorescent staining

Immunofluorescent staining was described as our previous study (4). For tissues, the liver sections were deparaffinized and antigen retrieved by Tris-EDTA buffer (pH 9.0) at 95°C for 40 min, and then blocked with 5% normal goat serum for 30 min at room temperature. The sections were then incubated with the primary antibodies including anti-RFP (1:400, Invitrogen, MA5-15257), anti-CK19 (1:10, DHSB, Troma-III), anti-Yap (1:500, CST, 14074)， anti-Numb (1:200, CST, 2756), Hnf4a (1:600, Abcam, ab181604) and anti-β-Catenin (1:200; BD Biosciences, 610154) overnight at 4°C, followed by washing with PBS and incubation with secondary antibodies (Alexa Fluor 488-conjugated anti–mouse IgG (A21202), Alexa Fluor 555–conjugated anti–rabbit IgG (A21428), Alexa Fluor 647-conjugated anti-rabbit IgG (A21246) Alexa Fluor 488-conjugated anti-rat IgG (A48262) or Alexa Fluor 555–conjugated anti–mouse IgG (A31570), all from Invitrogen) for 60 min at room temperature. After washing with PBS, the sections were mounted with Vectashield mounting medium containing DAPI (Vector Laboratories). For multiple staining with antibodies from the same species, we use the Tyramide SuperBoost™ Kits from Invitrogen. For the staining of MARK2 in tissues, the isolated liver tissues were immediately embedded in OCT and frozen for preparation of cryosections and then staining operation.

For cells, 4% PFA fixed cells were rinsed with PBS, permeabilized with 0.2% Triton X-100 in PBS for 10 min at 4°C, blocked with PBS containing 0.02% Triton X-100 and 5% BSA at RT for 30 min, and then incubated with primarily antibodies anti-SPTAN1 (1:200, Invitrogen, PA5-87363), anti-SPTAN1 (1:100, Santa Cruz, sc-48382), anti-SRC (1:100, Santa Cruz, sc-32789), anti-MARK2 (1:200, Sigma, HPA074905), anti-MST1 (1:250, Proteintech, 22245-1-AP), anti-YAP (1:200,

SCBT, sc-101199), anti-YAP (1:200, CST, 14074), anti-NUMB (1:200, CST, 2756), anti-β-Catenin (1:200; BD Biosciences, 610154), anti-HNF4α (1:200, abcam, ab181604), anti-SOX9 (1:200, Invitrogen, 14-9765-82), anti-FLAG (1:200, Novus, NBP1-06712SS), anti-HA (1:200, CST, 3724) or anti-MYC (1:200, SCBT, sc-40) at 4℃ for overnight, followed by PBS rinsing and incubating with secondary antibodies for another 1 h. Finally, after three times PBS washing, the cells were mounted with Vectashield mounting medium containing DAPI. All images were collected with a confocal microscope (Leica TCS SP8). The YAP nuclear/cytoplasmic staining intensity from the image was quantified using ImageJ software.

Quantitative real-time PCR

Total RNA of liver tissues or cells were isolated using TRIzol reagent (Invitrogen). 1 μg total RNA was used to synthesize cDNA using the PrimeScript RT reagent Kit with gDNA Eraser (Takara). The Real-time quantitative PCR was performed by using SYBR Premix Ex TaqII (Takara) and the Bio-Rad iCycler iQ system (Bio-Rad, Hercules, CA, USA). Gene expression data were normalized to 18S rRNA. The primer sequences for specific genes are in the Supplementary Table 5.

Colony formation assay

SNU-423 cells stably expressing vector or Flag-Myr-NUMB3 were seeded in six-well plates (1000–2000 cells per well) and cultured for 2 weeks. Colonies were fixed with methanol and stained with 0.1% crystal violet, and the number of colonies was counted with ImageJ.

EdU assays

Cell proliferation was assayed using a Cell-Light EdU Apollo488 In Vitro Kit. Briefly, cells were seeded on glass coverslips in 12-well plates at densities of 3-4 × 10^5 cells per well and then

incubated with the cell culture medium containing EdU for 30 min. The cells were then fixed and stained with Apollo488 fluorescent dye and DAPI followed by microscopic analysis.

Generation and delivery of recombinant adeno-associated virus (AAV)

Generation and delivery of recombinant AAV was as described in our previous study (3). Briefly, HEK293T cells were co-transfected with AAV control plasmid, *Cre* or *MARK2*, adenovirus helper plasmid PXX6 and AAV8 helper plasmid p5E18-VD28. Cells were harvested at 60 hours post transfection. After 3 times of freezing and thaw, the crude viruses were released from HEK293T cells, and then further purified with chloroform treatment and PEG8000-$(NH_4)_2SO_4$ partition. The fraction containing viruses was dialyzed in PBS and concentrated by Amicon Ultra centrifugal filter. The AAV titer was determined by RT-PCR using primers against the CMV promoter on the AAV vector. For animal infection, one 4-week-old male or female mouse received a tail vein injection of 1×10^{11} genome copies of AAV.

Human Liver and HCC Samples

Human samples in this study were obtained with informed consent from the human tissue banks of the Xiang'an hospital of Xiamen University and Zhongshan Hospital of Xiamen University. HCCs are staged according to the tumor/node/metastasis (TNM) staging system (as referred to https://www.cancer.gov/about-cancer/diagnosis-staging/staging). The Snap-frozen biopsies from specimens of normal liver tissue (distant from the tumor) and HCC were collected. The diagnosis of HCC or normal liver was confirmed based on histological findings by independent pathologists. Regarding correlation analysis, we took binary logarithm ratio of the protein's expression in the cancer tissue and the matched paracancerous tissue. The intensities of the immunoblot bands of

paired samples of adjacent tissue (N) and tumor tissue (T) were quantified using ImageJ software. The relative ratios of expression levels of NUMB, MARK2, p-MOB1, p-SPTAN1, p-YAP and WW45 in paired samples were calculated. A heatmap was used to represent the ratio of the relative expression of the proteins NUMB, MARK2, p-MOB1, p-SPTAN1, p-YAP and WW45 in the T and N sample from each patient. Clustering was performed by using centroid linkage with Euclidean distance. We used "Pearson correlation coefficient" to evaluate the correlation of the log-ratios of paired proteins among NUMB, MARK2, p-MOB1, p-YAP and WW45. Then the significance of correlation coefficients was tested against the null hypothesis of zero-correlation, using two-sided t test. We adjusted the *P* value using "Holm–Bonferroni" method and set 0.05 to the significance level. The tools for correlation analysis are available from R version 3.5.0, which can be downloaded from https://www.r-project.org. All experiments were performed with the approval of the Xiamen University Review Board, and informed consent was obtained from all patients.

Reference

1. Ji S, Liu Q, Zhang S, Chen Q, Wang C, Zhang W, et al. FGF15 Activates Hippo Signaling to Suppress Bile Acid Metabolism and Liver Tumorigenesis. *Dev Cell.* 2019;48(4):460-74 e9.
2. Katsuda T, Hosaka K, and Ochiya T. Generation of Chemically Induced Liver Progenitors (CLiPs) from Rat Adult Hepatocytes. *Bio Protoc.* 2018;8(2):e2689.
3. Liu Q, Li J, Zhang W, Xiao C, Zhang S, Nian C, et al. Glycogen accumulation and phase separation drives liver tumor initiation. *Cell.* 2021;184(22):5559-76 e19.
4. Zhang S, Chen Q, Liu Q, Li Y, Sun X, Hong L, et al. Hippo Signaling Suppresses Cell Ploidy and Tumorigenesis through Skp2. *Cancer Cell.* 2017;31(5):669-84 e7.

Figure S1 (Related to Figure 1). NUMB sense cell density to modulate YAP activity. (A) Growth curve assays of HepG2 or SK-HEP-1 cells cultured at low cell density (LCD) or high cell density (HCD). Data represent the mean ± SD from experimental triplicate experiments and the *P* values of two-tailed unpaired Student's t test. (B) Schematic diagram of cell density sensing proteins screening for Hippo/MST-YAP signaling regulation. The fold change of decreased or increased in C-CP of LCD than of HCD, and increased or decreased in M-CP of HCD than of LCD were > 1.5.

Figure S2 (Related to Figure 1 and S1). NUMB sense cell density to modulate YAP activity. (A) Knockdown efficiency of indicated siRNAs was analyzed by qPCR. Each bar represents the mean ± SD from experimental triplicate experiments. (B) Immunofluorescence staining of YAP (red) and DAPI (blue) in HepG2 cells transfected with indicated siRNAs and cultured at HCD. Scale bars, 10 μm.

Figure S3 (Related to Figure 2). NUMB1/2 repress the cell proliferation of NUMB KO HepG2 cells. (A) Immunoblot analysis of NUMB KO HepG2 cells stably expressing vector or Flag-tagged NUMB1-4. (B-C) Immunofluorescence staining of EdU (green) and DAPI (blue) (B) and the percentage of EdU positive cells (C) of WT and NUMB KO HepG2 cells expressing vector, Flag-tagged NUMB1-4 cultured at HCD. Scale bar, 50 μm. Data are presented as the mean ± SD from biological triplicate experiments and the *P* value was assessed using one-way ANOVA followed by Tukey's multiple comparisons test.

Figure S4 (Related to Figure 3). SPTAN1 is required for NUMB membrane retention at HCD. (A) Silver stain of SDS-PAGE of purified NUMB-containing protein complexes. (B) Knockdown efficiency of indicated siRNAs was analyzed by qPCR. Each bar represents the mean ± SD from experimental triplicate experiments. (C) Immunofluorescence staining of NUMB (red) and DAPI (blue) in HepG2 cells transfected with indicated siRNAs and cultured at HCD. Scale bars, 20 μm.

Figure S5 (Related to Figure 4). High cell density induces SPTAN1 phosphorylation for NUMB1/2 membrane retention. (A) Immunofluorescence staining of NUMB (green), SPTAN1 (red) and DAPI (blue) in WT or SPTAN1 Y1176F mutant HepG2 cells cultured at LCD or HCD. Scale bars, 20 μm. (B) Whole-cell lysates from WT and SPTAN1 Y1176F HepG2 cells cultured at different cell densities were collected for co-IP analysis.

Figure S6 (Related to Figure 4). SRC promotes SPTAN1 phosphorylation for NUMB1/2 membrane retention. (A) Immunoblot analysis of p-SRC, SRC and GAPDH in HepG2 cells cultured at different cell densities. (B) Immunofluorescence staining of SRC (green) and DAPI (blue) in HepG2 cells cultured at LCD or HCD. Scale bars, 20 μm. (C) Whole-cell lysates from HepG2 cells co-transfected with indicated siRNAs and cultured at HCD were collected for co-IP analysis. (D) Immunofluorescence staining of NUMB (green), SPTAN1 (red) and DAPI (blue) in HepG2 cells transfected with indicated siRNAs and cultured at HCD. Scale bars, 20 μm. (E)

Whole-cell lysates from HepG2 cells treated with vehicle or Bosutinib for 4h and cultured at HCD were collected for co-IP analysis. (F) Immunofluorescence staining of NUMB (green), SPTAN1 (red) and DAPI (blue) in HepG2 cells treated with vehicle or 20 μM Bosutinib for 4h and cultured at HCD. Scale bars, 20 μm.

Figure S7 (Related to Figure 4). SPTAN1 phosphorylation is required for Yap inactivation. (A-B) Immunoblot analysis of indicated proteins (A), and immunofluorescence staining of YAP (red) and DAPI (blue) (B) in WT and SPTAN1 Y1176F mutant HepG2 cells cultured at different cell densities. Scale bars, 20 μm.

Figure S8 (Related to Figure 5). NUMB1/2 sequestrate MARK on the plasma membrane at high cell density. (A-C) Commassie Blue staining of indicated recombinant proteins (marked by asterisks).

Figure S9 (Related to Figure 7). NUMB deficiency promotes Yap activation in the liver with DDC treatment. (A-C) The liver-to-body weight ratios (n=10, 10) (A), H&E staining and IHC staining of CK19 and Ki67 (B), and the percentage of CK19 or Ki67 positive cells in the liver periportal areas (C) of $Numb^{Ctr}$ and $Numb^{\Delta Hep}$ mice at 3 months old. Scale bars, 50 μm. (D) Immunoblot analysis of primary hepatocytes lysates of $Numb^{Ctr}$ and $Numb^{\Delta Hep}$ mice treated with chow or DDC. (E) H&E staining and IHC staining of CK19 and Ki67 of $Numb^{Ctr}$ and $Numb^{\Delta Hep}$ mice treated with 0.1% DDC. Scale bars, 50 μm. (F) Immunofluorescence staining of YAP (red)

and β-Catenin (green) in the liver sections of *Numb*^{Ctr} and *Numb*$^{\Delta Hep}$ mice treated with chow or DDC. Scale bars, 50 μm. (G and H) The percentage of SOX9 and HNF4α double positive cells (G), and qPCR analysis (H) in CLiPs (Chemically Induced Liver Progenitors) derived from primary hepatocytes of *Numb*^{Ctr} and *Numb*$^{\Delta Hep}$ mice. Data are presented as mean ± SD and the *P* value of two-tailed unpaired Student's t test in A, C, G and H.

Figure S10 (Related to Figure 8). WW45 competes with MARK to modulate Hippo kinase MST1/2 activity. (A) Immunofluorescence staining MARK2 (red) and β-Catenin (green) in the liver sections of *Numb*^{Ctr} and *Numb*$^{\Delta Hep}$ mice treated with chow or DDC. Scale bars, 25 μm. (B) Whole-cell lysates of hepatocytes isolated from mice were collected for co-IP analysis. (C) Immunoblot analysis of indicated proteins in the primary hepatocytes of *Numb*^{Ctr} and *Numb*$^{\Delta Hep}$ mice treated with with chow or DDC. (D and E) Immunoblot analysis of WW45 and GAPDH in primary or CLiPs treated mouse hepatocytes (D) or in PHH or HepG2 cells (E).

Figure S11 (Related to Figure 8 and S10). WW45 competes with MARK to modulate Hippo kinase MST1/2 activity. (A) Immunofluorescence staining of MST1 (green), WW45 (red) or MARK2 (red) and DAPI (blue) as indicated in HepG2 cells cultured at LCD and HCD. Scale bars, 20 μm. (B) Whole-cell lysates from HepG2 cells cultured at LCD and HCD were collected for co-IP analysis. (C) Diagram of the structures of full length or truncated MST1. AI, auto-inhibitory domain; SARAH, Salvador-RASSF-Hippo domain. (D-G) Whole-cell lysates from HEK293T cells co-transfected with indicated constructs were collected for co-IP analysis as indicated. (H)

Immunoblot analysis of indicated proteins in WT and NUMB KO HepG2 cells expressing vector or Flag-tagged WW45.

Figure S12 (Related to Figure 8). NUMB interfaces with WW45 to regulate YAP activity. (A-C) Immunofluorescence staining of YAP (red), β-Catenin (green) and DAPI (blue) (A), qPCR analysis of *Ctgf* and *Cyr61* (B), and H&E and IHC staining (C) in liver of *Ww45*/*Numb*^{Ctr}, *Numb*$^{\Delta Hep}$, *Ww45*$^{\Delta Hep}$ and *Ww45*/*Numb*$^{\Delta Hep}$ mice at 3 months old. Data are presented as mean ± SD and the *P* value was assessed using one-way ANOVA followed by Tukey's multiple comparisons test, Scale bars, 50 μm.

Figure S13 (Related to Figure 8). SPTAN1 interfaces with WW45 to restrain liver size and tumorigenesis. (A and B) Representative liver images and the liver-to-body weight ratios (3 months old, n=8, 8, 8, 8) (A), or the tumor numbers (6 months old, n=8, 8, 8, 8) (B) of *Ww45*^{Ctr} and *Ww45*$^{\Delta Hep}$ mice treated with *Sptan1* gRNA or control vector. (C and D) Immunofluorescence staining of YAP (red), β-Catenin (green) and DAPI (blue) (C), and immunoblot analysis of indicated proteins (D) in the liver of mice *Ww45*^{Ctr} and *Ww45*$^{\Delta Hep}$ mice treated with *Sptan1* gRNA or control vector at 3 months old. Scale bars, 50 μm. (E) Representative liver images, the liver-to-body weight ratios (3 months old, n=8, 8, 8, 8) and tumor numbers (6 months old, n=8, 8, 8) of *Ww45*^{Ctr} and *Ww45*$^{\Delta Hep}$ mice treated with *Sptan1* gRNA or control vector. (F) Immunoblot analysis of indicated proteins in the livers of *Ww45*^{Ctr}, *Ww45*$^{\Delta Hep}$ or *Ww45*$^{\Delta Hep}$*Yap*$^{\Delta hep/+}$ mice treated with *Sptan1* gRNA or control vector mice at 3 months old. Data are presented as mean ±

SD, the *P* value was assessed using two-tailed unpaired Student's t test in A, B and one-way ANOVA followed by Tukey's multiple comparisons test in E.

Figure S14 (Related to Figure 9). NUMB interfaces with WW45 to restrain liver dedifferentiation and tumorigenesis in a YAP- but not RBP-J-dependent manner. (A and B) Immunoblot analysis of indicated proteins (A), and the H&E and IHC staining of CK19 and Ki67 (B) in the liver sections of *Ww45*/*Numb*^{Ctr}, *Ww45*/*Numb*$^{\Delta\mathrm{Hep}}$, *Ww45*/*Numb*$^{\Delta\mathrm{Hep}}$*Rbpj*$^{\Delta\mathrm{Hep}}$ and *Ww45*/*Numb*$^{\Delta\mathrm{Hep}}$*Yap*$^{\Delta\mathrm{Hep}/+}$ mice 3 months old. Scale bars, 50 μm. (C) Immunofluorescence staining of tdTomato (red), CK19 (green) and DAPI (blue) in the liver sections of *Ww45*/*Numb*^{Ctr}, *Ww45*/*Numb*$^{\Delta\mathrm{Hep}}$, *Ww45*/*Numb*$^{\Delta\mathrm{Hep}}$*Rbpj*$^{\Delta\mathrm{Hep}}$ and *Ww45*/*Numb*$^{\Delta\mathrm{Hep}}$*Yap*$^{\Delta\mathrm{Hep}/+}$ mice (with tdTomato labeled hepatocytes) treated with AAV-Cre and DDC. Scale bars, 50 μm. (D) Immunofluorescence staining of SOX9 (red), HNF4α (green) and DAPI (blue) in primary and CLiPs treated mouse hepatocytes from indicated mice. Scale bars, 50 μm.

Figure S15 (Related to Figure 9). NUMB interfaces with WW45 to restrain liver size and tumorigenesis in a NICD-independent manner. (A-E) Immunoblot analysis of indicated proteins (A), qPCR analysis of indicated genes (B), representative liver images and the liver-to-body weight ratios (n=6, 6, 6, 6) (C), Ki67 staining (D) and the percentage of Ki67 positive cells (E) in the livers of *Ww45*/*Numb*^{Ctr} and *Ww45*/*Numb*$^{\Delta\mathrm{Hep}}$ mice treated with vehicle or DAPT for 1.5 months. Scale bars, 50 μm. (F) Representative liver images and tumor numbers (n=6, 6, 6, 6) of indicated mice treated with vehicle or DAPT for 3.5 months. Data are presented as mean ± SD

and the *P* value was assessed using one-way ANOVA followed by Tukey's multiple comparisons test in B, C, E and F.

Figure S16 (Related to Figure 10). The analysis of SPTAN1/NUMB-MARK/Hippo signaling in human liver cancer. (A and B) Immunoblot analysis (A) and a heatmap representation (B) of the ratios of the relative expression of the indicated proteins in the T and N samples from one patient. For the detection of p-Tyr of SPTAN1, the loading of immunoprecipitates was normalized according to the levels of total SPTAN1. The intensities of the immunoblot bands were quantified using ImageJ software. Clustering was performed by using Pearson correlation metric and centroid linkage.

Figure S17 (Related to Figure 10). NUMB3/4 isoforms that fail to activate MST1/2 are preferably expressed in human liver cancer. (A) Immunofluorescence staining of NUMB (red), YAP (green), β-Catenin (gray) and DAPI (blue) in sections of non-tumorous tissues and HCC tumor tissues of the different stages. Scale bars, 50 μm. (B and C) Immunofluorescence staining of NUMB (red), YAP (green), β-Catenin (gray) and DAPI (blue) in liver sections from WT mouse treated with DEN for 8 months. Scale bars, 25 μm.

Figure S18 (Related to Figure 10). NUMB3/4 isoforms promote HCC progression. (A) The ratio of relative expression levels of NUMB-PTB_S versus NUMB-PTB_L in indicated liver cancer cell lines. Each bar represents the mean ± SD from experimental triplicate experiments. (B and C)

Immunofluorescence staining of NUMB in HepG2 cells and SNU-423 cells cultured at LCD or HCD. Scale bars, 20 μm. (D-F) Immunoblot analysis of Flag-tagged Myr-NUMB3 and GAPDH (D), representative images of EdU assays (E) and colonies formation assays (F) of SNU-423 cells stably expressing vector or Myr-NUMB3. Scale bar, 50 μm. (G) The percentage of EdU positive cells or colonies number in SNU-423 cells stably expressing vector or Myr-NUMB3. Data are presented as mean ± SD and the *P* value of two-tailed unpaired Student's t test. (H-J) SNU-423 cells stably expressing vector or Flag-NUMB1-4 were transplanted into athymic mice (n = 6), 4 weeks after, image of tumor xenografts (H), tumor weight (I) and expression of indicated proteins (J) are shown. (K-N) SNU-423 cells stably expressing shControl or shRNA targeting NUMB3/4 were subcutaneously inoculated into athymic mice (n = 6), 4 weeks after, image of tumor xenografts (K), tumor weight (L), immunoblot (M) and qPCR (N) analysis of indicated proteins/genes are shown. Data are presented as mean ± SD and the *P* value was assessed using one-way ANOVA followed by Tukey's multiple comparisons test in I, L and N.

Supplementary tables

1. Supplementary Table 1. DIA Mass Spectrometry Analysis of Plasma Membrane/Cytoskeleton and Cytosol Fractions in HepG2 Cells Cultured at Low or High Cell Density.
2. Supplementary Table 2. Mass Spectrometry Analysis of MST2-containing Protein Complex in HepG2 Cells.
3. Supplementary Table 3. Mass Spectrometry Analysis of NUMB-containing Protein Complex in HepG2 Cells.
4. Supplementary Table 4. siRNA sequences.
5. Supplementary Table 5. qRT-PCR primers

Supplementary Table 1. DIA Mass Spectrometry Analysis of Plasma Membrane/Cytoskeleton and Cytosol Fractions in HepG2 Cells Cultured at Low or High Cell Density.

Replicate 1

Protein.Names	Genes	Cytosol Fractions of HCD	Membrance Fractions of HCD	Cytosol Fractions of LCD	Membrance Fractions of LCD
5NT3B_HUMAN	NT5C3B	14001.4	13350.1	21054	3462.24
AAK1_HUMAN	AAK1	18028.7	23817	25533	13242.9
ABCF1_HUMAN	ABCF1	84035.7	208477	179622	84702.2
ABCF2_HUMAN	ABCF2	165143	253353	263742	116713
ABL2_HUMAN	ABL2	4449.47	10342.9	6305.06	6552.3
ACACA_HUMAN	ACACA	30343.1	94851.5	92116.9	16146.7
ACTZ_HUMAN	ACTR1A	58011.4	73355.2	81383.7	32706.3
ADDA_HUMAN	ADD1	10331.6	18492	13327.7	10603.9
AGAP3_HUMAN	AGAP3	4076.31	7085.61	6005.88	3039.75
AIFM1_HUMAN	AIFM1	9057.74	44814.5	2671.83	80328.2
AIMP1_HUMAN	AIMP1	28630.1	102205	91576.2	52880.1
AIMP2_HUMAN	AIMP2	75868.4	243484	187801	94385.4
ALDH2_HUMAN	ALDH2	22038	58978	1922.05	89024.8
AMRA1_HUMAN	AMBRA1	6165.05	9069.63	10469.2	4968.85
ANFY1_HUMAN	ANKFY1	11068.2	37642.2	37734.5	6136.09
ANKZ1_HUMAN	ANKZF1	10228	17600.5	12294.1	9746.72
ANM3_HUMAN	PRMT3	45655.4	52642.2	62049.3	6965.67
ANS1A_HUMAN	ANKS1A	7209.36	8528.85	9905.35	4233.18
AP2A2_HUMAN	AP2A2	32145.6	78915.5	40906.7	42374.5
AP3B1_HUMAN	AP3B1	16343.4	22650.6	22039.9	7538.79
AP3M1_HUMAN	AP3M1	11476.1	20025	14724.8	7878.48
ARAP1_HUMAN	ARAP1	15303	25575.9	24497.5	9746.44
ARF6_HUMAN	ARF6	10702.7	30676.6	13432	20331.2
ARIP4_HUMAN	RAD54L2	3532.41	4188.05	4514.83	2366.8
ARL6_HUMAN	ARL6	6224.73	7073.19	8163.4	4604.4
ARY1_HUMAN	NAT1	2438.41	1562.85	3332.74	
BCL10_HUMAN	BCL10	13365.3	9067.96	16085.7	2883.41
BCR_HUMAN	BCR	12254.7	14004.5	16897.2	5818.64
BL1S3_HUMAN	BLOC1S3	3926.43	3595.53	4889.72	1953.7
BN3D2_HUMAN	BCDIN3D	3361.93	2917.37	4021.54	1424.47
BRAT1_HUMAN	BRAT1	22877.2	44590.8	49416.1	11249.2
BUB1B_HUMAN	BUB1B	14030	16480.5	19735.9	7424.14
C1QBP_HUMAN	C1QBP	26469.9	182660	3833.76	328874
C2D1A_HUMAN	CC2D1A	11713.1	23034.8	14745.9	14703.5

CAN7_HUMAN	CAPN7	7962.03	11204.8	12260.2	3754.71
CC124_HUMAN	CCDC124	79557.7	154621	182698	78890.5
CCNH_HUMAN	CCNH	15612.3	34008	23876.1	22018.7
CEP85_HUMAN	CEP85	7610.96	15989.6	15339.7	7986.72
CEP97_HUMAN	CEP97	5599.08	10812.3	12497.2	3177.76
CH10_HUMAN	HSPE1	308446	706691	54765.2	2018820
CH60_HUMAN	HSPD1	299622	948545	46553.9	1781430
CIP2A_HUMAN	CIP2A	10351.1	19136.1	19366.5	5338.49
CLH1_HUMAN	CLTC	209066	813891	757050	187747
CLIP1_HUMAN	CLIP1	62059.4	48282	83390.5	11597.2
CLU_HUMAN	CLUH	96508.5	186510	199738	64544.7
CMTR1_HUMAN	CMTR1	33224.9	59338.1	57893.9	32439.7
CNN2_HUMAN	CNN2	35861	39187.5	49098.9	20729.8
CNO10_HUMAN	CNOT10	10348.9	35996.7	16640.6	20346.9
CNOT9_HUMAN	CNOT9	8345.64	41453.8	15246.4	22042.9
COMD3_HUMAN	COMMD3	14927.1	15412.9	17345.4	9656.16
COPE_HUMAN	COPE	155200	135716	180656	83923.7
COPZ1_HUMAN	COPZ1	66555.7	47509.9	78143.8	24133.1
CPNE3_HUMAN	CPNE3	136408	110854	176816	14348
CSK_HUMAN	CSK	54287.1	57464	84365.4	9997.46
CUL5_HUMAN	CUL5	11199.9	13558.2	13936.8	5920.22
DAAF5_HUMAN	DNAAF5	27341.3	56551.9	59768.9	12887.1
DBR1_HUMAN	DBR1	17393.3	19269.9	23583.9	9805.36
DCAF1_HUMAN	DCAF1	16178.9	19938.7	18822.8	11048.9
DCTN1_HUMAN	DCTN1	66325.8	82469.8	93296.9	29140.6
DCTN2_HUMAN	DCTN2	77243.5	94251.3	109067	50800.2
DDI2_HUMAN	DDI2	46759.4	36678.8	60959.5	8511.45
DDX11_HUMAN	DDX11	6524.32	16661.3	13611.5	6035.07
DDX6_HUMAN	DDX6	40325.3	135437	77299.6	69517.8
DIAP1_HUMAN	DIAPH1	98673.6	92356.1	130963	30919.1
DIAP3_HUMAN	DIAPH3	8007.11	14996.8	10151.5	7682.48
DNA2_HUMAN	DNA2	6538.37	16316.3	14986.7	9927.69
DNJB1_HUMAN	DNAJB1	57853.8	64828.5	71053	40578.4
DOHH_HUMAN	DOHH	35450.3	33012.8	56411.8	4358.48
DPOD1_HUMAN	POLD1	27925	44542.2	38753.8	23081.6
DPOD2_HUMAN	POLD2	17440.5	19065.4	21887.9	10291.3
DPOLA_HUMAN	POLA1	22531	36993.3	37039.9	15936.5
DPOLM_HUMAN	POLM	1602.01	4254.94	2594.32	2756.21
DUS3L_HUMAN	DUS3L	44847.2	60639	80150	16916.4
DYL1_HUMAN	DYNLL1	99711.7	161564	131769	101740
DYR1A_HUMAN	DYRK1A	5180.14	6565.49	6836.63	4157.53
ECHM_HUMAN	ECHS1	53357.5	139307	6018.32	284519
EIF2D_HUMAN	EIF2D	26480.7	32132.9	35037.3	18323.9

EIF3A_HUMAN	EIF3A	131389	178441	174734	107891
EIF3C_HUMAN	EIF3C	170089	249037	230615	137682
EIF3D_HUMAN	EIF3D	89705.4	161174	121176	81128.7
EIF3E_HUMAN	EIF3E	128203	192182	169485	90388.2
ELP6_HUMAN	ELP6	7476.02	5642.46	9326.01	1456.35
EPS15_HUMAN	EPS15	15294.4	16097.2	19943.4	5475.9
ERC6L_HUMAN	ERCC6L	19153.6	14559.2	22908.1	5318.15
EST1A_HUMAN	SMG6	4609.76	10048.3	8256.38	3588.64
ETFB_HUMAN	ETFB	37314.5	63465.8	6990.81	123691
EXOC1_HUMAN	EXOC1	7376.2	20995.3	9898.44	13229.7
EXOC8_HUMAN	EXOC8	10801.9	42960.1	27360.2	23956.9
F120A_HUMAN	FAM120A	11379.3	53984.9	31396.8	27002
F262_HUMAN	PFKFB2	12898.9	12885.8	19590.5	3340.49
F263_HUMAN	PFKFB3	1065.39	4176.5	3601.75	2150.17
FANCI_HUMAN	FANCI	12075.8	42402.7	24677.6	22356.4
FAS_HUMAN	FASN	557757	494414	702321	64988.1
FBX3_HUMAN	FBXO3	15491.9	14078.4	22298.9	2139.12
FHOD1_HUMAN	FHOD1	12872.5	18585.9	15858.9	10707.1
FKBP5_HUMAN	FKBP5	26291.4	26318.7	38003.3	9950.39
FLII_HUMAN	FLII	48013.6	72876.8	68876.9	32409.5
FLNA_HUMAN	FLNA	142464	475199	419325	211861
FLNB_HUMAN	FLNB	115237	181127	178799	77255
FLNC_HUMAN	FLNC		23007.9	2485.14	12398.6
FNBP1_HUMAN	FNBP1	9590.35	8744.75	12675.5	3774.51
FXL12_HUMAN	FBXL12	5505.54	11393.8	6554.96	4799.54
GCP2_HUMAN	TUBGCP2	10574.2	28388.4	15635.9	14431.1
GEMI5_HUMAN	GEMIN5	71140.4	111336	108171	48313.9
GLRX5_HUMAN	GLRX5	7540.66	10036.4	1570.37	26572.4
GMFB_HUMAN	GMFB	45727.9	11142	53117.9	5033.44
HAP28_HUMAN	PDAP1	58049.2	56192.8	84496.9	28512.5
HAUS2_HUMAN	HAUS2	6830.67	11068	8600.83	6743.24
HAUS6_HUMAN	HAUS6	14727.9	35504.2	27358	20105.1
HAUS8_HUMAN	HAUS8	6824.37	27742.3	14670.3	17121.4
HECD1_HUMAN	HECTD1	50294.3	38771.1	70851.8	9340.58
HMCES_HUMAN	HMCES	10582.8	34505.6	24553.2	17785.3
HOOK1_HUMAN	HOOK1	9627.32	7961.94	14218.4	1943.55
HOOK3_HUMAN	HOOK3	7929.08	10346.9	12408.3	6080.08
HPBP1_HUMAN	HSPBP1	49248.2	41324.2	57787.1	15650.9
IDH3B_HUMAN	IDH3B	27109.7	86799.9	3594.42	164082
IF1AX_HUMAN	EIF1AX	97163.3	184312	216571	96172.7
IF2B_HUMAN	EIF2S2	79267	127835	107017	71487.4
IF2P_HUMAN	EIF5B	41299.1	101525	98244.5	48523.9
IF4E_HUMAN	EIF4E	42245	47224.5	55428.9	29990.6

IKKA_HUMAN	CHUK	3757.05	12049	14800.3	2953.48
IKKB_HUMAN	IKBKB	2341.21	7345.31	7863.86	1648.52
IPO5_HUMAN	IPO5	194290	140729	251123	33182.4
IQGA1_HUMAN	IQGAP1	179522	195222	224860	75823
IQGA3_HUMAN	IQGAP3	6086.1	14716.5	9725.67	8194.26
K1C16_HUMAN	KRT16	4727.07	75367.5	32167.8	9040.94
K2C80_HUMAN	KRT80	3357.1	7998.58	5685.61	4862.72
KBRS1_HUMAN	NKIRAS1	8137.16	6372.12	15648.4	3238.52
KCTD3_HUMAN	KCTD3	7891.27	12007.5	10601.1	6686.28
KIF11_HUMAN	KIF11	20035.7	30090	29922.1	15801.6
KIF1C_HUMAN	KIF1C	7477.88	10114.8	11817.7	5666.04
KIF3A_HUMAN	KIF3A	6027.97	6391.96	7081.61	3271.68
KINH_HUMAN	KIF5B	67286.6	63801.9	95049.7	22482.8
KITH_HUMAN	TK1	62154.5	58324	84726.6	12102.6
KLC1_HUMAN	KLC1	27386.3	27448.7	37344.8	10962.7
KLC2_HUMAN	KLC2	25250.8	40795.7	31788.1	24910.7
KPCD2_HUMAN	PRKD2	6219.49	11264.6	9275.31	3708.93
KTNB1_HUMAN	KATNB1	4132.26	5004.64	4954.47	3029.61
L2GL1_HUMAN	LLGL1	10768.4	18367	16825.4	6914.13
LARP1_HUMAN	LARP1	42096.8	97761	82519.5	52742.1
LIMD1_HUMAN	LIMD1	6245.57	8690.87	10982.5	3647.66
LIPA1_HUMAN	PPFIA1	10890.1	26870.8	24028.3	12588.2
LRC41_HUMAN	LRRC41	6358.97	18867.2	9333.43	11285.3
LRC47_HUMAN	LRRC47	89151.7	114738	167521	31547.3
LRRF1_HUMAN	LRRFIP1	16555.2	27674.7	26970.8	13168.8
LZTR1_HUMAN	LZTR1	1431.47	4086.7	3382.53	1383.63
M3K7_HUMAN	MAP3K7	10657.7	14817.7	13384.6	7847.42
MARE2_HUMAN	MAPRE2	24320	25438	36170	15543.1
MARK2_HUMAN	MARK2	7474.95	19758	19024.5	9700.21
MARK3_HUMAN	MARK3	15040.2	21250.1	19209.2	11452
MEMO1_HUMAN	MEMO1	43832	55178.2	70432.2	11627.4
MINK1_HUMAN	MINK1	8959.34	18305.1	15488.3	11526.4
MIO_HUMAN	MIOS	5149.55	9365.21	6985.94	6214.68
MMAC_HUMAN	MMACHC	4329.05	5104.76	8927.05	2270.82
MMS19_HUMAN	MMS19	41724.5	41787.8	54428.5	7928.8
MOCS3_HUMAN	MOCS3	7414.43	13476.8	9959.65	4061.2
MOES_HUMAN	MSN	640277	687208	1061580	339694
MP2K3_HUMAN	MAP2K3	37807.6	20488.7	45846	10375
MYL6_HUMAN	MYL6	440488	522892	689752	313889
MYL6B_HUMAN	MYL6B	6619.55	12133.2	11644.3	6627.46
MYO1E_HUMAN	MYO1E	20973.2	55155	43940	36388.9
NEK7_HUMAN	NEK7	16418.2	21890.8	23352.9	10673.1
NEK9_HUMAN	NEK9	27416.4	28299.8	35119.4	7670.57

NEMF_HUMAN	NEMF	16577.1	20981.6	26606.6	6898.34
NEMO_HUMAN	IKBKG	7824.64	10932.6	14754.2	4307.44
NSUN2_HUMAN	NSUN2	115098	132220	147803	69849.4
NUB1_HUMAN	NUB1	4953.31	4570.47	6159.91	1104.46
NUMB_HUMAN	NUMB	6317.38	16845.9	10568.9	11107
NUMBL_HUMAN	NUMBL	1297.49	2166.06	1954.44	1428.95
NXN_HUMAN	NXN	37828.9	14941.4	47446.9	3331.3
OAT_HUMAN	OAT	50435.8	101673	4856.3	177135
OTU1_HUMAN	YOD1	3620.24	6115.14	7851.74	
OTU6B_HUMAN	OTUD6B	19855.3	22654.7	42839.8	6814.99
OTU7B_HUMAN	OTUD7B	9945.71	10894.8	12087.7	5370.64
OTUD4_HUMAN	OTUD4	6484.8	21673.2	19760.8	12280.9
OTUL_HUMAN	OTULIN	25073.2	21075.3	35230.5	4371.04
P3C2A_HUMAN	PIK3C2A	7315.3	19062.9	11048.4	9319.58
P5CR3_HUMAN	PYCR3	58626.4	39811.6	69981.9	9073.32
PASK_HUMAN	PASK	4752.52	4688.29	5616.2	311.618
PCID2_HUMAN	PCID2	13550	23699.3	18606.3	11332.7
PDLI5_HUMAN	PDLIM5	42356.7	76171.2	69147.8	41448.3
PEF1_HUMAN	PEF1	44791.2	20632.8	71636.7	11069.4
PGP_HUMAN	PGP	42363.1	48925.8	52770.3	2421.78
PHLB1_HUMAN	PHLDB1	5047.95	9353.26	7605.45	6068.9
PHLB2_HUMAN	PHLDB2	2504.15	7814.43	5110.83	4974.03
PIN1_HUMAN	PIN1	22264.3	26437.9	41471	12477.1
PKN2_HUMAN	PKN2	24650.8	37880.5	29558.4	24239.5
PLAP_HUMAN	PLAA	32807.9	44551.9	51898.1	16122.9
PPAC_HUMAN	ACP1	35228.8	35422	58489.5	6974.19
PPID_HUMAN	PPID	81099	66512.3	112474	18879.8
PRDX3_HUMAN	PRDX3	170236	516803	21966.4	964302
PRI1_HUMAN	PRIM1	27196.2	31197.8	32914.2	11459.9
PSF2_HUMAN	GINS2	14516.8	9583.02	19012.6	4781.89
PSF3_HUMAN	GINS3	24511.5	17692.6	29942	8174.46
QRIC1_HUMAN	QRICH1	16228.7	22542.6	24315	7604.38
R113A_HUMAN	RNF113A	8485.37	12678.6	15205.7	6244.05
R3HD1_HUMAN	R3HDM1	742.605	2812.37	1836.61	1729.74
RA54B_HUMAN	RAD54B	2276.45	12745.9	7594.53	6681.31
RECQ4_HUMAN	RECQL4	2966.15	4116.35	3510.14	1956.89
RENT2_HUMAN	UPF2	9819.95	16595.5	14603.7	9104.5
REPS1_HUMAN	REPS1	5050.83	8357.91	10058.5	2742.43
RFWD3_HUMAN	RFWD3	3257.1	6780.01	6395.34	3737.43
RINI_HUMAN	RNH1	54744.5	125451	130248	35092.4
RL11_HUMAN	RPL11	93130.4	229378	207052	142545
RL35_HUMAN	RPL35	151054	605901	471862	372295
RL40_HUMAN	UBA52	1148.65	1953.66	3623.04	

RN214_HUMAN	RNF214	6475.91	13316.2	9462.71	6235.36
RNF31_HUMAN	RNF31	3814.87	11109.3	7969.74	3427.98
RO52_HUMAN	TRIM21	7583.84	9679.64	12262	
ROCK2_HUMAN	ROCK2	41348.4	44719.9	60839.3	16293.9
RP25L_HUMAN	RPP25L	5463.25	13058.5	11071.4	6711.17
RPAP3_HUMAN	RPAP3	17489.8	18434.7	23987.8	10386.8
RPC1_HUMAN	POLR3A	6603.86	16736.5	14511.1	7676.42
RPC2_HUMAN	POLR3B	18826.8	34376.1	35137.6	14952.3
RPC6_HUMAN	POLR3F	15581.4	24186.2	27805.8	15657.8
RRP44_HUMAN	DIS3	22395.6	60443	40248.2	35346.4
RS11_HUMAN	RPS11	228141	577250	539421	357436
RS17_HUMAN	RPS17	110553	366415	257009	230137
RS18_HUMAN	RPS18	440495	1405760	1117010	748308
RS2_HUMAN	RPS2	97229.9	295597	233783	195548
RS20_HUMAN	RPS20	133943	411366	338473	227803
RS25_HUMAN	RPS25	82278.9	347680	188018	180940
RS27L_HUMAN	RPS27L	20237.1	71681.1	36041.2	34453.4
RS5_HUMAN	RPS5	152655	541123	375389	248247
RS6_HUMAN	RPS6	83693.2	253874	188717	152107
RS8_HUMAN	RPS8	95109.5	328898	235418	205585
RS9_HUMAN	RPS9	226063	548903	528256	284752
RTEL1_HUMAN	RTEL1	2413.59	9002.51	5572.47	3756.8
RTF2_HUMAN	RTF2	10222.2	22893.7	25972.2	11200.8
S23IP_HUMAN	SEC23IP	46539.8	31901.1	60151.9	15652.5
SART3_HUMAN	SART3	20489.3	41917.5	39007.4	22208.8
SBDS_HUMAN	SBDS	75545	95155.4	127418	40094.8
SC23B_HUMAN	SEC23B	39535.3	43987.5	55578.7	18074.8
SC24B_HUMAN	SEC24B	10228.1	23817.1	17049.9	12537.9
SC31A_HUMAN	SEC31A	42938.9	85786.1	86498	27775.9
SCYL1_HUMAN	SCYL1	7963.03	18834.5	10565.8	9378.3
SHIP2_HUMAN	INPPL1	6938.7	9873.74	10007.3	4577.22
SHLB2_HUMAN	SH3GLB2	13493.9	17051.3	17550.4	9273.24
SIK2_HUMAN	SIK2	6984.4	9087.84	9004.05	4895.89
SIR1_HUMAN	SIRT1	10748	11115.9	12872.1	4432.58
SLBP_HUMAN	SLBP	22832.4	25916.3	31304	17196.8
SMC4_HUMAN	SMC4	54884.7	56672.1	78177.3	27662.3
SMG5_HUMAN	SMG5	9714.14	23019.8	15469.5	13277.3
SMRCD_HUMAN	SMARCAD1	10895.9	27430	17812.9	16206.4
SRP14_HUMAN	SRP14	54027.4	115848	95955.2	66668.8
SSU72_HUMAN	SSU72	16469.9	16701.8	27907.7	10555.9
STRP1_HUMAN	STRIP1	9180.72	11333.4	10715.6	7107.44
SYDC_HUMAN	DARS1	32276.4	186967	132530	102860
SYEP_HUMAN	EPRS1	113859	239144	216502	82375.7

SYIC_HUMAN	IARS1	46836.1	128268	114195	41256.3
SYK_HUMAN	KARS1	43331	93527.9	84816.4	40178.4
SYLC_HUMAN	LARS1	34767.9	76105.2	66330.8	25836.7
SYMC_HUMAN	MARS1	75729.6	158463	121290	58289.4
SYQ_HUMAN	QARS1	59772.7	130043	116976	47447.4
SYRC_HUMAN	RARS1	118866	193085	191696	69960.5
TAB3_HUMAN	TAB3	2587.51	4477.25	4039.38	2597.08
TACC3_HUMAN	TACC3	28594.1	31283.4	46416.8	11157.7
TARB1_HUMAN	TARBP1	6038.92	16348.2	13822.4	4763.54
TBCE_HUMAN	TBCE	30732.2	31333.1	45175.1	9382.33
TBD2B_HUMAN	TBC1D2B	6430.73	9219.55	9495.14	4079.09
TBK1_HUMAN	TBK1	8889.7	17919.3	14485.1	5741.4
TEDC1_HUMAN	TEDC1	2484.22	2071.72	3623.51	839.792
TELO2_HUMAN	TELO2	18854.7	29751.9	35303.5	8947
TES_HUMAN	TES	35052.7	34050.1	48634.9	8205.11
TEX30_HUMAN	TEX30	11735.6	16274.6	16094.1	8193.28
TF2B_HUMAN	GTF2B	4932.39	15840.9	10767.2	8878.58
TIM_HUMAN	TIMELESS	14955.1	29528.6	23200.9	13881.2
TLN1_HUMAN	TLN1	49498.9	93514.6	101332	23211.5
TNG6_HUMAN	TANGO6	13676.5	19069	17520.3	7083.83
TNIP1_HUMAN	TNIP1	4566.19	5962.38	7365.08	2375.01
TNPO1_HUMAN	TNPO1	135689	150679	191278	39386.6
TNPO3_HUMAN	TNPO3	46217.7	57193.2	61098.1	18949
TOP3B_HUMAN	TOP3B	2102.46	8887.33	4962.76	5272.89
TPC11_HUMAN	TRAPPC11	4474.36	7460.84	5880.1	4495.44
TPPC8_HUMAN	TRAPPC8	8526.22	12966.3	12768.3	5942.59
TRAF2_HUMAN	TRAF2	7208.72	15660.4	15037.1	4317.14
TRIP6_HUMAN	TRIP6	40912	85661.5	71594.5	26918.4
TRM1_HUMAN	TRMT1	19032.7	37517.8	37387.9	11156.2
TRM2A_HUMAN	TRMT2A	12826.9	26384.3	24690.6	13420.1
TS101_HUMAN	TSG101	27922.5	42016.7	33533.7	21409.8
TSR1_HUMAN	TSR1	75453.5	137896	123934	80328.8
TTC27_HUMAN	TTC27	22771.9	24433.2	28380.8	6786.61
TTC4_HUMAN	TTC4	50753.9	45489.7	63790.4	18470.5
TXND9_HUMAN	TXNDC9	30529.3	19373.6	45324.1	5832.06
TYDP2_HUMAN	TDP2	10350.8	11991.8	16366.5	5021.28
UBP16_HUMAN	USP16	12647.6	17085.5	18539.8	7923.95
UBXN1_HUMAN	UBXN1	76324	61431.7	98166.1	31548.7
UGDH_HUMAN	UGDH	114445	91380.1	138101	21564.1
UN45A_HUMAN	UNC45A	50243.8	53515.9	67522.1	16508.6
UPP1_HUMAN	UPP1	5757.04	9357.38	9965.81	2318.67
URFB1_HUMAN	UHRF1BP1	10472.7	19328	12272.2	8000.52
URGCP_HUMAN	URGCP	16576.6	14088.7	21683.7	4575.51

USP9X_HUMAN	USP9X	72009.4	97285.9	136818	12658.5
VCIP1_HUMAN	VCPIP1	15340.8	19016.7	22775	8759.93
VP33A_HUMAN	VPS33A	18922.9	40671	27200.3	24041
VPS16_HUMAN	VPS16	7461	20277	11041.2	11673.9
VPS18_HUMAN	VPS18	8403.67	21707.3	12884.3	13440.8
WDR6_HUMAN	WDR6	17136.9	16139.1	20194.3	5081.94
WDR62_HUMAN	WDR62	4532.64	12124.6	12483.2	2341.24
WDR7_HUMAN	WDR7	4092.53	8821.69	7486.35	5729.88
WDR70_HUMAN	WDR70	17039.6	36169.6	32350.5	23406.9
WRIP1_HUMAN	WRNIP1	19878	35432.2	35530.9	14674.7
XPO1_HUMAN	XPO1	124694	216916	182797	88162.4
XPO2_HUMAN	CSE1L	114836	131519	160291	32291.1
XPO5_HUMAN	XPO5	83880.2	158207	166567	49058.6
XPOT_HUMAN	XPOT	26559.1	41006.9	43156.4	4457.28
XRCC3_HUMAN	XRCC3	4578.02	10952.8	6912.67	5520.37
XRN1_HUMAN	XRN1	6672.35	19484.2	12603.7	7113.66
ZCCHL_HUMAN	ZC3HAV1L	8311.9	8506.45	11018.7	1871.06
ZYX_HUMAN	ZYX	97288.4	123760	147715	57409.4

Supplementary Table 1. DIA Mass Spectrometry Analysis of Plasma Membrane/Cytoskeleton and Cytosol Fractions in HepG2 Cells Cultured at Low or High Cell Density.

Replicate 2

Protein.Names	Genes	Cytosol Fractions of HCD	Membrance Fractions of HCD	Cytosol Fractions of LCD	Membrance Fractions of LCD
5NT3B_HUMAN	NT5C3B	10043.6	3823.265	10762.4	1011.75
AAK1_HUMAN	AAK1	6675.86	4314.66	9606.18	1808.13
ABCF1_HUMAN	ABCF1	190218	110367.5	223054	90456
ABCF2_HUMAN	ABCF2	138293	82580	169013	58046
ABL2_HUMAN	ABL2	4611.47	4069.005	5720.85	2700.23
ACACA_HUMAN	ACACA	52929.2	42040.1	76875.2	8002.6
ACTZ_HUMAN	ACTR1A	37319.2	22359.1	46245.3	5095.1
ADDA_HUMAN	ADD1	7224.34	7534.6	10553	3149.42
AGAP3_HUMAN	AGAP3	1577.26	1855.075	3075.13	1029.895
AIFM1_HUMAN	AIFM1	14070.3	13588.05	4782.74	25613.25
AIMP1_HUMAN	AIMP1	34720.4	39934.6	89764.9	6281.1
AIMP2_HUMAN	AIMP2	85162.2	77700.5	185515	11959.35
ALDH2_HUMAN	ALDH2	55159.8	36777.1	14598.3	68555
AMRA1_HUMAN	AMBRA1	4267.82	2215.38	7462.5	648.13
ANFY1_HUMAN	ANKFY1	15645.8	17532.5	45278.3	4406.84
ANKZ1_HUMAN	ANKZF1	8465.13	4733.14	9364.09	2610.885
ANM3_HUMAN	PRMT3	32731.6	16977.9	40240.9	3796.885
ANS1A_HUMAN	ANKS1A	7394.39	3852.445	9034.8	1110.875
AP2A2_HUMAN	AP2A2	22736.1	29140.65	32379.9	21816.75
AP3B1_HUMAN	AP3B1	63176.2	36510.45	74298.7	12933.7
AP3M1_HUMAN	AP3M1	9897.6	7619.15	13138.4	2905.29
ARAP1_HUMAN	ARAP1	23816.2	17207.25	32653.7	4920.955
ARF6_HUMAN	ARF6	18300.2	19997.7	27352.8	12303.8
ARIP4_HUMAN	RAD54L2	1341.04	1009.78	3006.49	493.934
ARL6_HUMAN	ARL6	6537.23	3109.3	8720.3	884.305
ARY1_HUMAN	NAT1	3815.53	2262.715	7804.92	0
BCL10_HUMAN	BCL10	9195.19	2785.355	12212.6	614.59
BCR_HUMAN	BCR	10991.3	5514.65	15605	2189.03
BL1S3_HUMAN	BLOC1S3	5989.33	1982.515	6167.22	814.945
BN3D2_HUMAN	BCDIN3D	1177.7	345.402	1268.65	0
BRAT1_HUMAN	BRAT1	13441	11076.35	36211.4	3493.01
BUB1B_HUMAN	BUB1B	24147.5	12129.15	30711.2	6241.95
C1QBP_HUMAN	C1QBP	119634	159212	24383.1	298605.5
C2D1A_HUMAN	CC2D1A	8537.47	7186.05	9333.92	3817.2
CAN7_HUMAN	CAPN7	9539.04	5347.85	11407	2579.86
CC124_HUMAN	CCDC124	115926	67696	160778	38842.9

CCNH_HUMAN	CCNH	11911.4	13048.05	21350.2	10138.95
CEP85_HUMAN	CEP85	7166.45	5362.15	15091.5	2553.13
CEP97_HUMAN	CEP97	6493.35	4936.77	11357.8	1673.77
CH10_HUMAN	HSPE1	649155	195876	197914	487506
CH60_HUMAN	HSPD1	642337	458618.5	183285	889835
CIP2A_HUMAN	CIP2A	6634.39	4667.885	10212	1466.92
CLH1_HUMAN	CLTC	130457	259346	460201	68663
CLIP1_HUMAN	CLIP1	23841	8136.5	28392.9	1456.365
CLU_HUMAN	CLUH	113060	47466.75	144874	18815.6
CMTR1_HUMAN	CMTR1	5412.33	9003.25	10132.1	7156.05
CNN2_HUMAN	CNN2	21728.4	9277.1	28587.7	5886.9
CNO10_HUMAN	CNOT10	9865.21	12940.95	15438.1	7729.5
CNOT9_HUMAN	CNOT9	16190.6	26468.6	37783.4	19461.65
COMD3_HUMAN	COMMD3	6357.91	4720.985	6719.45	2433.665
COPE_HUMAN	COPE	46903.9	24344.55	48759.2	17950.35
COPZ1_HUMAN	COPZ1	45521.6	17299.3	49927.3	14273.15
CPNE3_HUMAN	CPNE3	98524.6	44341.65	121891	10020.4
CSK_HUMAN	CSK	21490.5	12137.9	23576.8	6320.15
CUL5_HUMAN	CUL5	7674.16	4242.06	10597.7	2080.315
DAAF5_HUMAN	DNAAF5	40799.1	27966.7	57982.6	7578.25
DBR1_HUMAN	DBR1	16583.8	9349.4	22241.5	4801.22
DCAF1_HUMAN	DCAF1	8875.65	4437.685	11529.6	2850.515
DCTN1_HUMAN	DCTN1	46750	23170.15	56598.3	6373
DCTN2_HUMAN	DCTN2	67646.7	29566.45	76704.8	8584.65
DDI2_HUMAN	DDI2	29974.3	9122.8	34400.6	1972.655
DDX11_HUMAN	DDX11	5178.22	5315.5	12816.5	2299.655
DDX6_HUMAN	DDX6	19961.2	18373.65	30647.4	13628.85
DIAP1_HUMAN	DIAPH1	117769	41041.5	141639	10192.35
DIAP3_HUMAN	DIAPH3	8351.58	7190.1	9423.32	3707.695
DNA2_HUMAN	DNA2	10371.2	6532.55	19872.8	2381.29
DNJB1_HUMAN	DNAJB1	63679.7	35386.35	87764.8	19330.25
DOHH_HUMAN	DOHH	18776.9	5004.7	30614.1	1654.98
DPOD1_HUMAN	POLD1	24542.4	13988.4	31042.3	5867.7
DPOD2_HUMAN	POLD2	19409.6	7578.3	21773.6	3214.33
DPOLA_HUMAN	POLA1	15176.5	9179.35	19164.5	3540.12
DPOLM_HUMAN	POLM	951.579	1487.14	1729.21	533.11
DUS3L_HUMAN	DUS3L	42367.5	28271.15	79224.9	12828.35
DYL1_HUMAN	DYNLL1	78423.8	56575	101017	39281.6
DYR1A_HUMAN	DYRK1A	2862.93	2375.125	3694.59	1622.71
ECHM_HUMAN	ECHS1	150634	57854	38954.3	118053
EIF2D_HUMAN	EIF2D	42971.5	23830.95	47275.5	11058.45
EIF3A_HUMAN	EIF3A	148077	77461	240310	33821.35
EIF3C_HUMAN	EIF3C	137359	66359.5	214690	26809.7

EIF3D_HUMAN	EIF3D	62389.8	34418.45	96433.6	13408.05
EIF3E_HUMAN	EIF3E	135136	71947.5	221751	27318.45
ELP6_HUMAN	ELP6	6913.55	2776.385	8103.23	754.445
EPS15_HUMAN	EPS15	10019.4	6497.5	12633.8	1936.88
ERC6L_HUMAN	ERCC6L	13234	4498.82	16368.1	2785.17
EST1A_HUMAN	SMG6	6453.81	5551.7	8621.54	3171.45
ETFB_HUMAN	ETFB	40818.2	15256.95	13835.4	31275.9
EXOC1_HUMAN	EXOC1	7148.51	12088.7	10394.4	6275.55
EXOC8_HUMAN	EXOC8	9168.33	11234.15	16856.7	4191.4
F120A_HUMAN	FAM120A	16185	16387.15	25280.7	8395.15
F262_HUMAN	PFKFB2	10556.9	6710.55	12599.5	3108.425
F263_HUMAN	PFKFB3	755.766	3047.485	2891.69	2025.895
FANCI_HUMAN	FANCI	15389.6	22271.4	33891.2	11058
FAS_HUMAN	FASN	701531	304484.5	881982	43416.4
FBX3_HUMAN	FBXO3	7253.57	3216.55	10572	657.805
FHOD1_HUMAN	FHOD1	14699.4	8741.7	17243.6	4815.665
FKBP5_HUMAN	FKBP5	16812.8	8630.1	23549.1	1541.55
FLII_HUMAN	FLII	40222.2	18433.7	47645.1	7443.4
FLNA_HUMAN	FLNA	178107	311701	401098	180266
FLNB_HUMAN	FLNB	79820.2	65935	161303	25724.1
FLNC_HUMAN	FLNC		15872.4	3642.48	6708.25
FNBP1_HUMAN	FNBP1	8611.89	3558.645	9635.9	1768.475
FXL12_HUMAN	FBXL12	6888.99	5353.45	12027.2	2273.33
GCP2_HUMAN	TUBGCP2	23785.4	23986.25	29265.4	9011.25
GEMI5_HUMAN	GEMIN5	75090.7	44466.5	117638	17459.1
GLRX5_HUMAN	GLRX5	8202.79	2541.72	2758.97	5074.1
GMFB_HUMAN	GMFB	5940.69	1523.52	8152.98	807.58
HAP28_HUMAN	PDAP1	138161	68095	178501	19767.35
HAUS2_HUMAN	HAUS2	3600.25	3826.605	7466.81	2346.77
HAUS6_HUMAN	HAUS6	7377.29	7412.8	14320.6	3232.97
HAUS8_HUMAN	HAUS8	4821	6934	10109.9	3748.255
HECD1_HUMAN	HECTD1	35287.7	12074.05	59311.1	2968.045
HMCES_HUMAN	HMCES	6340.12	7567.15	17253.9	3930.05
HOOK1_HUMAN	HOOK1	12209.2	4556.245	14933	1178.78
HOOK3_HUMAN	HOOK3	15347.3	8479.7	21490.7	3322.38
HPBP1_HUMAN	HSPBP1	35326.8	14607.4	50035.6	6187.2
IDH3B_HUMAN	IDH3B	53894.3	27299.05	11214.2	55879
IF1AX_HUMAN	EIF1AX	160467	117547	233012	69777.5
IF2B_HUMAN	EIF2S2	62338.6	40615	80342.8	27851.95
IF2P_HUMAN	EIF5B	73822.2	39884.2	93845	23389.4
IF4E_HUMAN	EIF4E	45613.1	20689.15	50489.7	9180.5
IKKA_HUMAN	CHUK	4049.03	4173.1	7641.81	813.72
IKKB_HUMAN	IKBKB	5844.86	5166.95	10117.3	800.23

IPO5_HUMAN	IPO5	244578	82904.5	302552	23508.15
IQGA1_HUMAN	IQGAP1	69256.5	61701.5	99263.4	21278.1
IQGA3_HUMAN	IQGAP3	4216.79	6376.05	5591.59	3311.06
K1C16_HUMAN	KRT16	1314.34	3925.19	2159.28	2463.345
K2C80_HUMAN	KRT80	2979.04	3319.64	6353.61	2358.73
KBRS1_HUMAN	NKIRAS1	2702.41	1902.96	4753.54	1411.475
KCTD3_HUMAN	KCTD3	7168.61	8683.4	9742.18	3659.305
KIF11_HUMAN	KIF11	12021	8386.65	25334.2	4695.215
KIF1C_HUMAN	KIF1C	3721.06	1846.7	3916.23	966.715
KIF3A_HUMAN	KIF3A	7909.33	3361.75	9961.23	1513.22
KINH_HUMAN	KIF5B	49280	28896.65	66389.4	10424.45
KITH_HUMAN	TK1	56692.6	26312.75	82791.5	20821.05
KLC1_HUMAN	KLC1	31958.3	16471.4	46517	4680.645
KLC2_HUMAN	KLC2	17336.4	13484.05	27161.3	5776.05
KPCD2_HUMAN	PRKD2	4973.92	4718.935	7238.04	1268.615
KTNB1_HUMAN	KATNB1	3648.61	3354.315	4294.02	1569.455
L2GL1_HUMAN	LLGL1	13880.9	8613.65	15065	2720.44
LARP1_HUMAN	LARP1	65567.4	42071.1	98516.2	27444.5
LIMD1_HUMAN	LIMD1	3756.86	2541.37	7814.77	543.46
LIPA1_HUMAN	PPFIA1	6623.52	14563.9	27992.8	7338.15
LRC41_HUMAN	LRRC41	13498.3	15272.9	19644.1	7878.75
LRC47_HUMAN	LRRC47	116487	56951	168873	17832.75
LRRF1_HUMAN	LRRFIP1	28268.7	11040.4	35850	4375.26
LZTR1_HUMAN	LZTR1	10637.3	4464.945	16059.4	3635.04
M3K7_HUMAN	MAP3K7	6944.41	5935.8	10347.1	3704.57
MARE2_HUMAN	MAPRE2	12093.9	5213.95	13697	3195.04
MARK2_HUMAN	MARK2	4457.05	5981.2	8724.11	4661.935
MARK3_HUMAN	MARK3	6425.14	5482.9	7581.72	3297.08
MEMO1_HUMAN	MEMO1	21469.5	17361.75	29903.4	6684.3
MINK1_HUMAN	MINK1	13034.3	11326.95	17956.1	7467
MIO_HUMAN	MIOS	4035.35	3527.785	5429.81	2113.68
MMAC_HUMAN	MMACHC	4157.4	1823.53	6335.52	713.89
MMS19_HUMAN	MMS19	69357	29976.8	82103.4	6225.45
MOCS3_HUMAN	MOCS3	2293.74	1385.41	3961.97	279.5675
MOES_HUMAN	MSN	353387	145082	403169	90317.5
MP2K3_HUMAN	MAP2K3	24158.8	8722.25	25166.5	3749.03
MYL6_HUMAN	MYL6	122927	157962.5	220192	53128.5
MYL6B_HUMAN	MYL6B	6907.23	9080.3	9235.04	2659.175
MYO1E_HUMAN	MYO1E	20735.9	22789.25	41809.2	13910.35
NEK7_HUMAN	NEK7	8805.83	6559.5	14328.4	4623.81
NEK9_HUMAN	NEK9	12264.9	6203.85	15629.1	2699.48
NEMF_HUMAN	NEMF	14642.7	6389.15	15574.5	1786.75
NEMO_HUMAN	IKBKG	6373.99	5034.05	11048.9	1098.71

NSUN2_HUMAN	NSUN2	56245.7	28334.1	93111.3	12853.15
NUB1_HUMAN	NUB1	6701.37	1800.485	7438.97	772.265
NUMB_HUMAN	NUMB	5867.25	5627.35	6164.15	3429.365
NUMBL_HUMAN	NUMBL	1257.2	881.425	1634.6	0
NXN_HUMAN	NXN	9931.75	1799.62	10796.6	716.51
OAT_HUMAN	OAT	113975	30906.75	19743.8	73693.5
OTU1_HUMAN	YOD1	2686.53	1310.63	4894.08	0
OTU6B_HUMAN	OTUD6B	18234.7	8378.45	26078.4	2009.95
OTU7B_HUMAN	OTUD7B	10520.7	5247.5	11488.6	2389.575
OTUD4_HUMAN	OTUD4	6008.94	7756.4	13833.1	2691.76
OTUL_HUMAN	OTULIN	26069.1	9344.3	35086.1	1586.47
P3C2A_HUMAN	PIK3C2A	8535.62	8797.35	9664.98	4303.835
P5CR3_HUMAN	PYCR3	33866.6	19890.15	44435.2	12331.15
PASK_HUMAN	PASK	3680.81	1092.67	4151.21	0
PCID2_HUMAN	PCID2	11875	8880.25	17188.9	6957.8
PDLI5_HUMAN	PDLIM5	30608.2	22763.25	37748.7	17639.95
PEF1_HUMAN	PEF1	5889.55	6838.05	7626.88	4997.345
PGP_HUMAN	PGP	20504.5	8958.15	26276.7	879.36
PHLB1_HUMAN	PHLDB1	6203.81	6143.15	11622.6	3155.89
PHLB2_HUMAN	PHLDB2	4572	10768.2	8436.56	4100.44
PIN1_HUMAN	PIN1	14830.6	9387.75	19438.7	5116.8
PKN2_HUMAN	PKN2	29769.4	21292.4	33650.4	12192
PLAP_HUMAN	PLAA	38425.8	23903.65	60690.7	6182.3
PPAC_HUMAN	ACP1	26687.4	11444.65	40642	3007.61
PPID_HUMAN	PPID	60132.8	23836.9	88796.2	4116.615
PRDX3_HUMAN	PRDX3	267455	108177	57457	267604.5
PRI1_HUMAN	PRIM1	18726.5	9900.4	21591.3	5443.25
PSF2_HUMAN	GINS2	8365.06	2198.4	10298.2	552.995
PSF3_HUMAN	GINS3	10802.7	4630.875	14003.7	1660.505
QRIC1_HUMAN	QRICH1	14285.8	6996.6	18797.3	1905.36
R113A_HUMAN	RNF113A	18927.2	18677.9	40455.9	3939.35
R3HD1_HUMAN	R3HDM1		449.135	504.696	217.939
RA54B_HUMAN	RAD54B	2852.93	3958.37	5694.44	2461.23
RECQ4_HUMAN	RECQL4	1822.89	1624.245	1928.39	768.025
RENT2_HUMAN	UPF2	6167.21	4670.985	9684.28	2721.97
REPS1_HUMAN	REPS1	3973.41	3274.67	7524.08	1295.505
RFWD3_HUMAN	RFWD3	2297.2	3162.61	6525.56	1577.695
RINI_HUMAN	RNH1	75525.5	72505	143465	26880.2
RL11_HUMAN	RPL11	47099.8	42023.75	75910.6	34114.95
RL35_HUMAN	RPL35	243927	164988.5	253045	122715.5
RL40_HUMAN	UBA52	526.125	715.475	575.536	0
RN214_HUMAN	RNF214	7670.68	4932.44	9839.85	1915.495
RNF31_HUMAN	RNF31	5182.79	5967.3	8246.16	1792.78

RO52_HUMAN	TRIM21	2491.89	1444.25	4826.61	0
ROCK2_HUMAN	ROCK2	14171	8085.75	18837	3318.78
RP25L_HUMAN	RPP25L	4461.47	2813.615	5589.37	1612.215
RPAP3_HUMAN	RPAP3	16629.8	8754.15	24685.8	5467.7
RPC1_HUMAN	POLR3A	5649.97	6483.15	10719	2800.025
RPC2_HUMAN	POLR3B	9939.92	6048.5	15022	3018.775
RPC6_HUMAN	POLR3F	9590.91	4650.605	13334.3	2248.86
RRP44_HUMAN	DIS3	20243.4	20198.6	34433.8	13398.35
RS11_HUMAN	RPS11	315952	199965	400893	155753.5
RS17_HUMAN	RPS17	176542	98641.5	196957	70432
RS18_HUMAN	RPS18	340463	221424.5	415527	151545
RS2_HUMAN	RPS2	154268	104446.5	161529	75327
RS20_HUMAN	RPS20	220134	135267.5	265178	102183
RS25_HUMAN	RPS25	103716	82187.5	117948	63680.5
RS27L_HUMAN	RPS27L	34321.3	27151.7	36808.1	17008.55
RS5_HUMAN	RPS5	213900	130458.5	250558	95361.5
RS6_HUMAN	RPS6	82988.1	66127	139929	54204.5
RS8_HUMAN	RPS8	126583	101809.5	137739	72698
RS9_HUMAN	RPS9	246534	159274.5	309794	118728.5
RTEL1_HUMAN	RTEL1	2721.28	3897.865	5140.76	1167.655
RTF2_HUMAN	RTF2	7306.35	7162.6	10827.4	5253.05
S23IP_HUMAN	SEC23IP	56172.3	18138.6	68645	7984.65
SART3_HUMAN	SART3	23453.1	19076.05	42547.1	7310.1
SBDS_HUMAN	SBDS	83344.9	49387.95	94690.5	23240.15
SC23B_HUMAN	SEC23B	48492.4	30882	49711.7	19781.35
SC24B_HUMAN	SEC24B	8907.91	11680.1	14242	7940.25
SC31A_HUMAN	SEC31A	27108	30202.05	56437.6	11595.95
SCYL1_HUMAN	SCYL1	6234.1	7747.1	10588.9	4768.465
SHIP2_HUMAN	INPPL1	10101.9	6061.5	15380.2	3148.57
SHLB2_HUMAN	SH3GLB2	24212	8084	24878.2	2263.025
SIK2_HUMAN	SIK2	3314.57	1620.525	4386.16	0
SIR1_HUMAN	SIRT1	10717.3	4962.14	19018	1049.29
SLBP_HUMAN	SLBP	23356.3	14704.5	32683.9	9089.45
SMC4_HUMAN	SMC4	27233	17266.9	28535.6	14050.85
SMG5_HUMAN	SMG5	2239.14	2143.19	3850.21	930.26
SMRCD_HUMAN	SMARCAD1	5286.55	6127.3	12123.5	3774.475
SRP14_HUMAN	SRP14	40066.2	35524.25	56375.4	20244.9
SSU72_HUMAN	SSU72	16008.5	8216.25	23751.8	3735.21
STRP1_HUMAN	STRIP1	3836.49	1709	4626.92	838.63
SYDC_HUMAN	DARS1	56653.1	76224	151028	15525.8
SYEP_HUMAN	EPRS1	181579	124320	308254	21158.25
SYIC_HUMAN	IARS1	61468.6	48612.65	93897.9	6796.6
SYK_HUMAN	KARS1	141720	68357	200069	18716.7

SYLC_HUMAN	LARS1	68825.6	61199.5	119393	7331.25
SYMC_HUMAN	MARS1	158481	82845.5	192282	11717.55
SYQ_HUMAN	QARS1	43464.1	33937.55	83929.3	7951.7
SYRC_HUMAN	RARS1	146039	92742.5	260925	24712.1
TAB3_HUMAN	TAB3	1489.14	1732.505	2135.66	1392.88
TACC3_HUMAN	TACC3	19051.3	11354.15	68820.5	3795.195
TARB1_HUMAN	TARBP1	11590.6	12891.45	15241.5	5660.05
TBCE_HUMAN	TBCE	32361.6	13765.75	35906.9	3302.18
TBD2B_HUMAN	TBC1D2B	19394.6	10221.15	31406.9	5076.6
TBK1_HUMAN	TBK1	9048.71	6655.2	11816.7	3536.82
TEDC1_HUMAN	TEDC1	1813.52	2314.965	2697.7	1111.545
TELO2_HUMAN	TELO2	25243.9	13024.15	37610.5	4197.71
TES_HUMAN	TES	31100.1	13093.05	36190.1	3326.4
TEX30_HUMAN	TEX30	8469.59	4294.91	10756.2	2267.395
TF2B_HUMAN	GTF2B	5975.47	8412.1	13472	3904.28
TIM_HUMAN	TIMELESS	15867.2	12516.5	22927.4	5735.4
TLN1_HUMAN	TLN1	41940.6	35020.75	75607.2	7182.35
TNG6_HUMAN	TANGO6	8209.2	3623.275	9655.42	1418.81
TNIP1_HUMAN	TNIP1	3069.45	1347.56	5239.33	781.15
TNPO1_HUMAN	TNPO1	73595.2	46677	110377	14233.1
TNPO3_HUMAN	TNPO3	34411.4	17173.5	49998.4	5529.85
TOP3B_HUMAN	TOP3B	2542.32	2682.19	3111.38	1343.08
TPC11_HUMAN	TRAPPC11	7080.99	5798.3	10077.3	4189.7
TPPC8_HUMAN	TRAPPC8	6075.83	4200.14	8232.07	1647.58
TRAF2_HUMAN	TRAF2	6042.87	5716.8	12201.7	3332.32
TRIP6_HUMAN	TRIP6	40443.9	32627.35	77349.9	11562.05
TRM1_HUMAN	TRMT1	32818	24138.45	71472.6	7042.7
TRM2A_HUMAN	TRMT2A	6523.94	4994.21	8727.86	2792.115
TS101_HUMAN	TSG101	23050.3	13125.55	25255.6	6850.5
TSR1_HUMAN	TSR1	88388.8	60862	131195	41670.75
TTC27_HUMAN	TTC27	13561.5	7076.15	18746.8	2111.13
TTC4_HUMAN	TTC4	41061.1	11557.85	51920.8	3733.88
TXND9_HUMAN	TXNDC9	18274.2	4521.875	26514.5	935.595
TYDP2_HUMAN	TDP2	12054.5	6833.45	22172.8	1721.025
UBP16_HUMAN	USP16	11935.9	5100.25	13459.1	3002.38
UBXN1_HUMAN	UBXN1	63567.6	22317.7	68878.6	9918.05
UGDH_HUMAN	UGDH	112864	42277.1	127005	8048.1
UN45A_HUMAN	UNC45A	35254.4	15095.7	44035	8481.4
UPP1_HUMAN	UPP1	7666.84	5345	13984.2	1910.985
URFB1_HUMAN	UHRF1BP1	11626.9	8099.7	14472.5	2334.515
URGCP_HUMAN	URGCP	9939	4579.69	15757.6	2377.205
USP9X_HUMAN	USP9X	70291	38326.4	93723.2	6289.6
VCIP1_HUMAN	VCPIP1	19251	6424.1	20939.5	2747.84

VP33A_HUMAN	VPS33A	15294.6	12842.4	16571.5	8179.75
VPS16_HUMAN	VPS16	6927.19	8205.95	7739.98	6272.45
VPS18_HUMAN	VPS18	6863.59	9564.45	10196.4	5975.15
WDR6_HUMAN	WDR6	22824.5	11842.15	32622.4	5974.15
WDR62_HUMAN	WDR62	3675.27	3741.505	8629.87	538.31
WDR7_HUMAN	WDR7	2937.64	2993.42	4002.09	1422.215
WDR70_HUMAN	WDR70	10213.7	9462.7	16128.7	4969.72
WRIP1_HUMAN	WRNIP1	25036.7	18251.25	45702.6	6127.95
XPO1_HUMAN	XPO1	110156	92999	180930	48625
XPO2_HUMAN	CSE1L	211775	79341.5	262324	14221.65
XPO5_HUMAN	XPO5	95202.9	66495.5	153248	16853.7
XPOT_HUMAN	XPOT	45533.3	32369	67389.2	3037.96
XRCC3_HUMAN	XRCC3	8586.58	7696.35	12491.6	6104.95
XRN1_HUMAN	XRN1	4744.75	5738.7	7470.5	2428.365
ZCCHL_HUMAN	ZC3HAV1L	5474.65	3241.48	7282.09	1378.455
ZYX_HUMAN	ZYX	91421.1	49877.05	144376	19586.75

Supplementary Table 2. Mass Spectrometry Analysis of MST2-containing Protein Complex in HepG2 Cells.

Protein Name	Coverage (%)	Peptides	Unique Peptides	Spectrum	Avg. Mass
MST2_HUMAN	99	499	379	12751	56301
5NT3B_HUMAN	13	4	4	6	34389
AAK1_HUMAN	10	6	6	10	103885
ABCF1_HUMAN	11	8	8	9	95926
ABCF2_HUMAN	35	30	30	91	71290
ABL2_HUMAN	18	15	13	18	128343
ACACA_HUMAN	3	4	4	4	265551
ACTZ_HUMAN	50	13	12	28	42614
ADDA_HUMAN	18	10	9	15	80955
AGAP3_HUMAN	12	8	5	12	95044
AIFM1_HUMAN	18	8	8	9	66901
AIMP1_HUMAN	30	6	6	8	34353
AIMP2_HUMAN	19	4	4	5	35349
ALDH2_HUMAN	34	14	14	17	56381
AMRA1_HUMAN	25	23	23	38	142507
ANFY1_HUMAN	14	13	12	13	128399
ANKZ1_HUMAN	10	6	6	7	80927
ANM3_HUMAN	11	4	4	5	59903
ANS1A_HUMAN	4	4	4	5	123108
AP2A2_HUMAN	12	9	3	12	103960
AP3B1_HUMAN	17	16	16	24	121320
AP3M1_HUMAN	36	12	12	23	46939
ARAP1_HUMAN	5	6	6	7	162191
ARF6_HUMAN	41	6	6	8	20082
ARIP4_HUMAN	10	10	10	12	162768
ARL6_HUMAN	21	4	4	4	21097
ARY1_HUMAN	11	4	4	5	33899
ARY1_HUMAN	6	2	2	2	33899
BCL10_HUMAN	28	8	8	12	26252
BCR_HUMAN	14	17	15	22	142819
BL1S3_HUMAN	29	4	4	11	21256
BN3D2_HUMAN	20	4	4	6	33200
BRAT1_HUMAN	6	4	4	5	88119
BUB1B_HUMAN	15	12	11	23	119545
C1QBP_HUMAN	34	8	2	37	31362
C2D1A_HUMAN	33	30	28	45	104062
CAN7_HUMAN	16	12	12	15	92652
CC124_HUMAN	38	10	10	17	25835
CCNH_HUMAN	18	6	6	7	37643

CEP85_HUMAN	14	9	9	11	85639
CEP97_HUMAN	7	5	5	6	96981
CH10_HUMAN	45	6	3	10	10932
CH60_HUMAN	59	50	50	180	61055
CIP2A_HUMAN	5	5	5	6	102185
CLH1_HUMAN	6	8	8	8	191613
CLIP1_HUMAN	6	7	7	7	162246
CLU_HUMAN	28	29	2	57	146669
CMTR1_HUMAN	9	8	8	13	95321
CNN2_HUMAN	17	4	3	5	33697
CNO10_HUMAN	15	9	7	11	82310
CNOT9_HUMAN	22	5	5	7	33631
COMD3_HUMAN	32	5	5	7	22151
COPE_HUMAN	53	17	17	48	34482
COPZ1_HUMAN	51	9	9	24	20198
CPNE3_HUMAN	22	11	11	16	60131
CSK_HUMAN	28	13	13	32	50704
CUL5_HUMAN	28	21	21	32	90955
DAAF5_HUMAN	15	10	10	16	93521
DBR1_HUMAN	7	4	4	4	61555
DCAF1_HUMAN	9	12	12	17	169007
DCTN1_HUMAN	24	28	28	46	141694
DCTN2_HUMAN	32	10	3	16	44231
DDI2_HUMAN	21	5	5	8	44523
DDX11_HUMAN	17	14	1	22	108313
DDX6_HUMAN	26	8	8	13	54417
DIAP1_HUMAN	25	29	29	41	141347
DIAP3_HUMAN	19	20	20	29	136926
DNA2_HUMAN	29	30	29	59	120415
DNJB1_HUMAN	51	19	19	33	38044
DOHH_HUMAN	25	6	6	7	32904
DPOD1_HUMAN	40	44	42	89	123631
DPOD2_HUMAN	43	18	18	43	51290
DPOLA_HUMAN	6	7	7	7	165912
DPOLM_HUMAN	17	6	6	7	54816
DUS3L_HUMAN	10	4	4	10	72594
DYL1_HUMAN	52	5	5	7	10366
DYR1A_HUMAN	7	4	4	4	85584
ECHM_HUMAN	48	13	13	53	31387
EIF2D_HUMAN	28	12	12	24	64707
EIF3A_HUMAN	47	85	85	168	166569
EIF3C_HUMAN	33	37	36	82	105344
EIF3D_HUMAN	16	7	7	14	63973

EIF3E_HUMAN	43	21	21	44	52221
ELP6_HUMAN	34	9	9	17	29793
EPS15_HUMAN	11	7	7	9	98656
ERC6L_HUMAN	8	8	8	8	141103
EST1A_HUMAN	5	6	6	7	160461
ETFB_HUMAN	41	9	9	15	27844
EXOC1_HUMAN	8	6	6	6	101982
EXOC8_HUMAN	6	4	4	4	81799
F120A_HUMAN	11	9	9	10	121888
F262_HUMAN	53	24	18	52	58477
F263_HUMAN	33	20	15	38	59609
FANCI_HUMAN	24	25	6	35	149323
FAS_HUMAN	23	50	49	79	273424
FBX3_HUMAN	14	4	4	7	54561
FHOD1_HUMAN	14	14	14	17	126551
FKBP5_HUMAN	24	9	9	11	51212
FLII_HUMAN	15	17	17	34	144751
FLNA_HUMAN	7	15	13	17	280737
FLNB_HUMAN	8	15	14	15	278162
FLNC_HUMAN	6	12	10	14	291020
FNBP1_HUMAN	8	4	4	5	71307
FXL12_HUMAN	24	5	5	7	37026
GCP2_HUMAN	10	7	7	7	102534
GEMI5_HUMAN	21	26	26	45	168589
GLRX5_HUMAN	53	10	10	27	16628
GMFB_HUMAN	40	7	6	7	16713
HAP28_HUMAN	41	10	10	14	20630
HAUS2_HUMAN	20	5	5	6	26933
HAUS6_HUMAN	10	10	10	10	108621
HAUS8_HUMAN	12	4	4	4	44857
HECD1_HUMAN	2	5	5	5	289383
HMCES_HUMAN	35	11	11	15	40575
HOOK1_HUMAN	7	4	4	4	84648
HOOK3_HUMAN	14	8	8	9	83126
HPBP1_HUMAN	30	8	8	19	39303
IDH3B_HUMAN	34	18	18	52	42184
IF1AX_HUMAN	49	6	6	23	16460
IF2B_HUMAN	29	11	11	13	38388
IF2P_HUMAN	9	10	10	12	138827
IF4E_HUMAN	33	11	11	25	25097
IKKA_HUMAN	16	10	10	17	84640
IKKB_HUMAN	7	6	6	7	86564
IPO5_HUMAN	35	33	29	77	123630

IQGA1_HUMAN	23	32	30	44	189251
IQGA3_HUMAN	2	4	2	6	184698
K1C16_HUMAN	65	38	18	124	51268
K2C80_HUMAN	16	6	5	7	50525
KBRS1_HUMAN	36	7	6	9	21643
KCTD3_HUMAN	9	5	5	5	88984
KIF11_HUMAN	51	69	68	193	119159
KIF1C_HUMAN	9	7	3	13	122947
KIF3A_HUMAN	19	12	9	14	80041
KINH_HUMAN	53	50	34	107	109685
KLC1_HUMAN	14	7	5	13	65310
KLC2_HUMAN	11	5	3	7	68935
KPCD2_HUMAN	14	11	8	17	96722
KTNB1_HUMAN	17	8	8	11	72334
L2GL1_HUMAN	16	12	12	17	115418
LARP1_HUMAN	15	12	12	16	123510
LIMD1_HUMAN	12	5	5	11	72190
LIPA1_HUMAN	26	21	20	29	135778
LRC41_HUMAN	15	12	12	14	88650
LRC47_HUMAN	30	14	14	15	63473
LRRF1_HUMAN	16	11	11	22	89253
LZTR1_HUMAN	9	5	5	6	94719
M3K7_HUMAN	9	4	4	7	67196
MARE2_HUMAN	28	7	6	12	37031
MARK2_HUMAN	7	5	4	5	87911
MARK3_HUMAN	8	4	3	5	84428
MEMO1_HUMAN	27	6	6	9	33733
MINK1_HUMAN	18	22	8	34	149821
MIO_HUMAN	7	6	6	7	98584
MMAC_HUMAN	42	8	8	15	31728
MMS19_HUMAN	13	12	12	21	113289
MOCS3_HUMAN	23	8	8	13	49669
MOES_HUMAN	8	5	5	5	67820
MP2K3_HUMAN	46	13	10	25	39318
MYL6_HUMAN	78	12	10	71	16930
MYL6B_HUMAN	22	4	2	13	22764
MYO1E_HUMAN	5	4	4	4	127062
NEK7_HUMAN	43	11	11	31	34551
NEK9_HUMAN	22	20	20	30	107168
NEMF_HUMAN	7	7	7	8	122954
NEMO_HUMAN	16	6	6	10	48198
NSUN2_HUMAN	44	34	34	74	86471
NUB1_HUMAN	22	11	11	13	70538

NUMB_HUMAN	12	7	6	7	70804
NUMBL_HUMAN	14	7	6	8	64891
NXN_HUMAN	38	15	15	28	48392
OAT_HUMAN	45	15	15	30	48535
OTU1_HUMAN	16	4	4	8	38322
OTU6B_HUMAN	21	5	5	6	33813
OTU7B_HUMAN	7	6	6	6	92526
OTUD4_HUMAN	7	6	6	7	124045
OTUL_HUMAN	22	8	8	11	40263
P3C2A_HUMAN	5	7	6	8	190678
P5CR3_HUMAN	46	11	11	30	28663
PASK_HUMAN	10	8	8	9	142929
PCID2_HUMAN	21	8	8	13	46030
PDLI5_HUMAN	11	5	5	5	63945
PEF1_HUMAN	16	5	5	15	30381
PGP_HUMAN	33	9	9	15	34006
PHLB1_HUMAN	10	10	10	10	151161
PHLB2_HUMAN	9	9	8	12	142158
PIN1_HUMAN	58	8	8	44	18243
PKN2_HUMAN	51	48	44	83	112035
PLAP_HUMAN	15	9	9	12	87157
PPAC_HUMAN	78	13	10	69	18042
PPID_HUMAN	24	6	6	10	40764
PRDX3_HUMAN	50	17	17	89	27693
PRI1_HUMAN	34	11	11	17	49902
PSF2_HUMAN	35	7	7	13	21428
PSF3_HUMAN	49	11	11	26	24535
QRIC1_HUMAN	14	11	11	15	86436
R113A_HUMAN	22	6	6	7	38787
R3HD1_HUMAN	4	4	3	7	120696
RA54B_HUMAN	12	8	8	8	102967
RECQ4_HUMAN	8	6	6	6	133067
RENT2_HUMAN	4	4	4	5	147810
REPS1_HUMAN	8	5	5	5	86662
RFWD3_HUMAN	9	6	6	7	85094
RINI_HUMAN	24	9	9	14	49974
RL11_HUMAN	51	17	17	100	20252
RL35_HUMAN	20	4	4	6	14551
RL40_HUMAN	43	13	13	102	14728
RN214_HUMAN	13	8	8	8	77667
RNF31_HUMAN	9	8	8	12	119652
RO52_HUMAN	35	15	15	25	54170
ROCK2_HUMAN	13	16	14	24	160899

RP25L_HUMAN	38	7	7	7	17631
RPAP3_HUMAN	27	14	14	18	75719
RPC1_HUMAN	16	19	19	21	155641
RPC2_HUMAN	8	10	10	10	127785
RPC6_HUMAN	20	5	5	6	35684
RRP44_HUMAN	49	50	48	134	109003
RS11_HUMAN	65	17	17	42	18431
RS17_HUMAN	75	14	14	58	15550
RS18_HUMAN	72	27	27	123	17719
RS2_HUMAN	58	19	19	58	31324
RS20_HUMAN	67	24	24	134	13373
RS25_HUMAN	43	7	7	24	13742
RS27L_HUMAN	43	7	3	27	9477
RS5_HUMAN	66	29	29	159	22876
RS6_HUMAN	23	5	5	6	28681
RS8_HUMAN	23	5	5	7	24205
RS9_HUMAN	39	13	13	24	22591
RTEL1_HUMAN	6	6	6	6	133683
RTF2_HUMAN	30	8	8	12	33887
S23IP_HUMAN	25	27	27	43	111076
SART3_HUMAN	31	26	26	54	109935
SBDS_HUMAN	48	13	13	23	28764
SC23B_HUMAN	25	17	14	27	86479
SC24B_HUMAN	16	15	14	33	137417
SC31A_HUMAN	15	14	14	14	133015
SCYL1_HUMAN	13	8	8	12	89631
SHIP2_HUMAN	29	31	31	50	138599
SHLB2_HUMAN	31	9	9	9	43974
SIK2_HUMAN	8	6	6	6	103915
SIR1_HUMAN	14	8	8	12	81681
SLBP_HUMAN	17	5	5	7	31286
SMC4_HUMAN	42	59	59	110	147182
SMG5_HUMAN	7	7	6	9	113928
SMRCD_HUMAN	9	7	7	11	117402
SRP14_HUMAN	59	10	10	31	14570
SSU72_HUMAN	39	7	7	8	22574
STRP1_HUMAN	5	5	5	5	95576
SYDC_HUMAN	16	5	5	7	57136
SYEP_HUMAN	38	59	59	117	170590
SYIC_HUMAN	30	36	35	78	144498
SYK_HUMAN	10	4	4	5	68048
SYLC_HUMAN	44	55	54	110	134466
SYMC_HUMAN	22	16	16	29	101116

SYQ_HUMAN	12	7	7	7	87799
SYRC_HUMAN	16	9	9	10	75379
TAB3_HUMAN	12	6	6	6	78653
TACC3_HUMAN	18	14	14	18	90360
TARB1_HUMAN	4	5	5	7	181674
TBCE_HUMAN	25	8	8	9	59346
TBD2B_HUMAN	9	6	6	7	109880
TBK1_HUMAN	17	10	10	11	83642
TEDC1_HUMAN	17	6	6	8	54231
TELO2_HUMAN	14	10	10	12	91747
TES_HUMAN	27	13	13	20	47996
TEX30_HUMAN	23	5	5	7	25585
TF2B_HUMAN	51	15	15	22	34833
TIM_HUMAN	6	5	5	5	138658
TLN1_HUMAN	7	13	12	15	269765
TNG6_HUMAN	5	4	4	6	120747
TNIP1_HUMAN	10	5	5	5	71864
TNPO1_HUMAN	14	11	11	20	102355
TNPO3_HUMAN	5	5	5	6	104203
TOP3B_HUMAN	9	7	7	7	96662
TPC11_HUMAN	4	4	4	5	128881
TPPC8_HUMAN	9	12	12	13	160996
TRAF2_HUMAN	14	5	5	9	55859
TRIP6_HUMAN	23	9	9	27	50288
TRM1_HUMAN	25	14	14	17	72234
TRM2A_HUMAN	18	9	9	9	68726
TS101_HUMAN	35	13	13	25	43944
TSR1_HUMAN	7	5	5	5	91810
TTC27_HUMAN	10	6	6	6	96632
TTC4_HUMAN	64	29	29	72	44679
TXND9_HUMAN	31	7	7	10	26534
TYDP2_HUMAN	32	9	9	17	40930
UBP16_HUMAN	5	4	4	5	93570
UBXN1_HUMAN	70	23	23	50	33325
UGDH_HUMAN	22	10	10	14	55024
UN45A_HUMAN	27	23	23	28	103077
UPP1_HUMAN	15	4	4	5	33934
URFB1_HUMAN	8	8	7	12	159484
URGCP_HUMAN	18	15	15	22	104987
USP9X_HUMAN	2	4	3	4	292278
VCIP1_HUMAN	15	13	13	14	134320
VP33A_HUMAN	10	4	3	4	67611
VPS16_HUMAN	13	9	9	9	94694

VPS18_HUMAN	9	7	7	9	110186
WDR6_HUMAN	7	5	5	8	121724
WDR62_HUMAN	4	4	4	4	165953
WDR7_HUMAN	14	16	16	20	163809
WDR70_HUMAN	17	9	9	9	73201
WRIP1_HUMAN	11	6	6	7	72133
XPO1_HUMAN	33	33	32	80	123386
XPO2_HUMAN	29	30	30	58	110417
XPO5_HUMAN	20	19	19	30	136311
XPOT_HUMAN	15	12	12	16	109964
XRCC3_HUMAN	36	10	10	19	37850
XRN1_HUMAN	5	7	7	9	194106
ZCCHL_HUMAN	31	7	7	13	32962
ZYX_HUMAN	10	4	4	6	61277

Supplementary Table 3. Mass Spectrometry Analysis of NUMB-containing Protein Complex in HepG2 Cells.

Protein Name	Coverage (%)	Peptides	Unique Peptides	Spectrum	Avg. Mass
NUMB_HUMAN	58	125	106	70804	54027
SPTAN1_HUMAN	17	38	34	44	284538
ZO1_HUMAN	1	2	2	2	195457
ZO2_HUMAN	53	64	32	156	133958
PATJ_HUMAN	15	24	24	36	196367
AMOT_HUMAN	40	42	42	81	118085
WWC1_HUMAN	7	6	6	8	125301
α-Catenin_HUMAN	4	3	3	4	100071
β-Catenin_HUMAN	7	5	3	6	85681
NF2_HUMAN	10	5	2	7	69690
FRMD6_HUMAN	8	4	4	4	72044
AJUBA_HUMAN	7	2	2	3	56934
MARK1_HUMAN	20	11	11	16	89003
MARK2_HUMAN	8	5	4	5	87911
MARK3_HUMAN	4	2	1	2	84428
RASSF1_HUMAN	7	2	2	2	39219
MST1_HUMAN	44	18	9	35	55630
MST2_HUMAN	5	2	2	2	56301

Supplementary Table 4. siRNA sequences

siRNAs	Sense（5'-3'）	Antisense（5'-3'）
ABCF2-1	GGUCGUGAGCGAUAAGACATT	UGUCUUAUCGCUCACGACCTT
ABCF2-2	UGAGCUUCAAGUAUACAAATT	UUUGUAUACUUGAAGCUCATT
ACP1-1	CUAUGUAUGGAUGAAAGCAAUTT	AUUGCUUUCAUCCAUACAUAGTT
ACP1-2	GAACUACUUGGGAGCUAUGAUTT	AUCAUAGCUCCCAAGUAGUUCTT
ADD1-1	CGGUGUAAAUUGGCAGCGUUUTT	AAACGCUGCCAAUUUACACCGTT
ADD1-2	GCAGAAUUUACAGGACAUUAATT	UUAAUGUCCUGUAAAUUCUGCTT
AMOT-1	GCAAGAGUUGGAAGGAUGCUATT	UAGCAUCCUUCCAACUCUUGCTT
AMOT-2	CGACACAUCGAAAUCCGAGAUTT	AUCUCGGAUUUCGAUGUGUCGTT
ANKZF1-1	GCUGACAAAUCAACACGUAAUTT	AUUACGUGUUGAUUUGUCAGCTT
ANKZF1-2	CAGUAAGAGAGGAGAGAAATT	UUUCUCUCCUCUCUUACUGTT
AJUBA-1	GGACCGGGAUUAUCACUUUTT	AAAGUGAUAAUCCCGGUCCTT
AJUBA-2	CCAAGUAUACUGUGUCACCTT	GGUGACACAGUAUACUUGGTT
CCDC124-1	CGAGAAAGCCAAGAGCCAUCUTT	AGAUGGCUCUUGGCUUUCUCGTT
CCDC124-2	GAAACAGCUGCUCAAGAAGGATT	UCCUUCUUGAGCAGCUGUUUCTT
C1QBP-1	GUGAUGAAAUUAAGGAGGAAATT	UUUCCUCCUUAAUUUCAUCACTT
C1QBP-2	GCACCAGGAGUACAUUACUUUTT	AAAGUAAUGUACUCCUGGUGCTT
CIP2A-1	CGGCACAAUCUUUCUGUUCAATT	UUGAACAGAAAGAUUGUGCCGTT
CIP2A-2	GUCUAGGAUUAUUGGCAAATT	UUUGCCAAUAAUCCUAGACTT
CNN2-1	AACCGGCUCCUGUCCAAAUAUGATT	UCAUAUUUGGACAGGAGCCGGUUTT
CNN2-2	UCAAGGCCAUAUCCCAAUATT	UAUUGGGAUAUGGCCUUGATT
CPNE3-1	GGUGGAGUGUUAUGAUUAUTT	AUAAUCAUAACACUCCACCTT
CPNE3-2	GCAGACAGCUUCUCAAUAUTT	AUAUUGAGAAGCUGUCUGCTT
CSK-1	GCAUUUUACAAGAAGUACGAAUCTT	GAUUCGUACUUCUUGUAAAAUGCTT
CSK-2	GAUGGAAUUGUAAUAAACCACGCCATT	UGGCGUGGUUUAUUACAAUUCCAUCUCTT
CTNNA1-1	GUAAAGGGCCCUCUAAUAAUU	AAUUAUUAGAGGGCCCUUUAC
CTNNA1-2	GAAGAGAGGUCGUUCUAAGUU	AACUUAGAACGACCUCUCUUC
CTNNB1-1	GGAUGUUCACAACCGAAUUTT	AAUUCGGUUGUGAACAUCCTT
CTNNB1-2	GCUUGGAAUGAGACUGCUGAUTT	AUCAGCAGUCUCAUUCCAAGCTT
EXOC1-1	CCAGAGCUGAACAACCUAAUUTT	AAUUAGGUUGUUCAGCUCUGGTT
EXOC1-2	GAAAGCAACCACCUAAUUCAUTT	AUGAAUUAGGUGGUUGCUUUCTT
FAM120A-1	GCCUUGAAUAAUGACUCUAAATT	UUUAGAGUCAUUAUUCAAGGCTT
FAM120A-2	GCUGACUAUGUACGCAACAUUTT	AAUGUUGCGUACAUAGUCAGCTT
FHOD1-1	CAGCGAGAGGAGCAUCUACAATT	UUGUAGAUGCUCCUCUCGCUGTT
FHOD1-2	AGGGUCAACGCUAUCUUGGAATT	UUCCAAGAUAGCGUUGACCCUTT
FLNA-1	GGCAAAAGUGACCGCCAAUAACGACTT	GUCGUUAUUGGCGGUCACUUUUGCCUCTT
FLNA-2	GUGACCGCCAAUAACGACATT	GUGACCGCCAAUAACGACATT
FLNB-1	ACGCAUUGACAUCCAGAUGAATT	UUCAUCUGGAUGUCAAUGCGUTT
FLNB-2	CCUUCAGGAAUCGGGAUUAATT	UUAAUCCCGAUUCCUGAAGGTT
FLNC-1	CGGUACCUUUGACAUCUACUATT	UAGUAGAUGUCAAAGGUACCGTT
FLNC-2	GCUAAGGUGGUUCCCAACAAUTT	AUUGUUGGGAACCACCUUAGCTT

FRMD6-1	GCACAUUCCAAACAUGCACAATT	UUGUGCAUGUUUGGAAUGUGCTT
FRMD6-2	CCACAGACUAUAUGUCGGAAATT	UUUCCGACAUAUAGUCUGUGGTT
HECTD1-1	GGCACACUUACAGCGAGUUTT	AACUCGCUGUAAGUGUGCCTT
HECTD1-2	GCACUUUGUAAUCGUUUGGTT	CCAAACGAUUACAAAGUGCTT
HSPD1-1	CCUGCUCUUGAAAUUGCCAAUTT	AUUGGCAAUUUCAAGAGCAGGTT
HSPD1-2	GCAAUGACCAUUGCUAAGAAUTT	AUUCUUAGCAAUGGUCAUUGCTT
HSPE1-1	GCUGCUGAAACUGUAACCAAATT	UUUGGUUACAGUUUCAGCAGCTT
HSPE1-2	GUUCUAGAUGACAAGGAUUAUTT	AUAAUCCUUGUCAUCUAGAACTT
INPPL1-1	GACUACCUGAAAGGCAGCUAUTT	AUAGCUGCCUUUCAGGUAGUCTT
INPPL1-2	CCUGAACUACAUCAGCAGGAATT	UUCCUGCUGAUGUAGUUCAGGTT
KCTD3-1	GCCCAUAUUUGGAUUGGACUATT	UAGUCCAAUCCAAAUAUGGGCTT
KCTD3-2	CGAGAUGAAACUGGUGCUAUATT	UAUAGCACCAGUUUCAUCUCGTT
KIF5B-1	UGAAUUGCUUAGUGAUGAATT	UUCAUCACUAAGCAAUUCATT
KIF5B-2	AAACCGAGUUCCCUAUGUAAATT	UUUACAUAGGGAACUCGGUUUTT
KLC2-1	CGCGCUCAUGAGAAAGAGUUUTT	AAACUCUUUCUCAUGAGCGCGTT
KLC2-2	CUAUCGGGAUCAGAACAAGUATT	UACUUGUUCUGAUCCCGAUAGTT
LIMD1-1	GCAGAAUGGCUGCAAAUUUAATT	UUAAAUUUGCAGCCAUUCUGCTT
LIMD1-2	GUCUGCAGCAUGGAUAAGUATT	UACUUAUCCAUGCUGCAGACTT
LRRC41-1	CAUCUCCACCUUGGAGCUAUUTT	AAUAGCUCCAAGGUGGAGAUGTT
LRRC41-2	GCAAACACUCAAAGAGUACAATT	UUGUACUCUUUGAGUGUUUGCTT
LRRFIP1-1	GGUUAUCACCCAGAUUAGATT	UCUAAUCUGGGUGAUAACCTT
LRRFIP1-2	AAUGGAGAGACUUCCGACATT	UGUCGGAAGUCUCUCCAUUTT
MAP2K3-1	CCGCAGAGCGUAUGAGCUATT	UAGCUCAUACGCUCUGCGGTT
MAP2K3-2	UGGACAAGUUCUACCGGAATT	UUCCGGUAGAACUUGUCCATT
MARK2-1	GGAAAGAGGUAGCUGUGAAGATT	UCUUCACAGCUACCUCUUUCCTT
MARK2-2	AACGCAGAAAAUAAGCGGCCUTT	AGGCCGCUUAUUUUCUGCGUUTT
MARK3-1	CGGAAACUACAGACUGUUGAATT	UUCAACAGUCUGUAGUUUCCGTT
MARK3-2	CCAAUUAAACGCGGCACUCUATT	UAGAGUGCCGCGUUUAAUUGGTT
MSN-1	GCAUUGACGAAUUUGAGUCUATT	UAGACUCAAAUUCGUCAAUGCTT
MSN-2	GCGGAUUAACAAGCGGAUCUUTT	AAGAUCCGCUUGUUAAUCCGCTT
MYO1E-1	GAAGAGAUACAUGGAUGACUATT	UAGUCAUCCAUGUAUCUCUUCTT
MYO1E-2	GCGUCAUUAUCAGUGGUGAAATT	UUUCACCACUGAUAAUGACGCTT
NF2-1	GCUUCGUGUUAAUAAGCUGAUTT	AUCAGCUUAUUAACACGAAGCTT
NF2-2	GCUCUGGAUAUUCUGCACAAUTT	AUUGUGCAGAAUAUCCAGAGCTT
NUMB-1	CAGCCACUGAACAAGCAGATT	UCUGCUUGUUCAGUGGCUGTT
NUMB-2	CUAAGGACCUCAUAGUUGATT	UCAACUAUGAGGUCCUUAGTT
PATJ-1	CCUGAUUAUGAAGUAAUGGUUTT	AACCAUUACUUCAUAAUCAGGTT
PATJ-2	GCAAGAAGAUUUGCCUUUAUATT	UAUAAAGGCAAAUCUUCUUGCTT
PDAP1-1	GCAGUCACUCUCCCUGAAUAATT	UUAUUCAGGGAGAGUGACUGCTT
PDAP1-2	GAGAAGAAAUCUCUAGACUCATT	UGAGUCUAGAGAUUUCUUCUCTT
PDLIM5-1	UGUUAGGUAGUUAUGAGUAAATT	UUUACUCAUAACUACCUAACATT
PDLIM5-2	CGCCCAUUGUAACCAGGUCAUTT	AUGACCUGGUUACAAUGGGCGTT
PHLDB2-1	CAGCGAGUCCUCUUAUCUATT	UAGAUAAGAGGACUCGCUGTT

PHLDB2-2	CUGUUGAGAACGAUUCCCAAATT	UUUGGGAAUCGUUCUCAACAGTT
PKN2-1	CAAAUGAGAUGUUUGCUAUTT	AUAGCAAACAUCUCAUUUGTT
PKN2-2	CAGACUAAUGAAUUGGCUUTT	AAGCCAAUUCAUUAGUCUGTT
PPFIA1-1	CCACAAAGCUCUGGAUGAATT	UUCAUCCAGAGCUUUGUGGTT
PPFIA1-2	GAGGAGAUUGAAAGUCGAGUUTT	AACUCGACUUUCAAUCUCCUCTT
PRKD2-1	CUGCAAGUUUAACUGUCACAATT	UUGUGACAGUUAAACUUGCAGTT
PRKD2-2	UUGUGACAGUUAAACUUGCAGTT	CUGCAAGUUUAACUGUCACAATT
REPS1-1	GCGAUCAUACAAAUCCCACUATT	UAGUGGGAUUUGUAUGAUCGCTT
REPS1-2	GCCUGAUCUAAACGGAUUUAUTT	AUAAAUCCGUUUAGAUCAGGCTT
SCYL1-1	CCCGUGUCCAUCUUCGUCUAUTT	AUAGACGAAGAUGGACACGGGTT
SCYL1-2	CCUGUCCAAAUUGGAGUCUGUTT	ACAGACUCCAAUUUGGACAGGTT
SH3GLB2-1	CACCAAGAACUGGACAGAGAATT	UUCUCUGUCCAGUUCUUGGUGT
SH3GLB2-2	CGUCAAGUCUCAGACAACCUATT	UAGGUUGUCUGAGACUUGACGTT
SPTAN1-1	GCCAUUGUUAAGCUGGAUGAATT	UUCAUCCAGCUUAACAAUGGCTT
SPTAN1-2	GCCACUGAACUGAAAGGAAUATT	UAUUCCUUUCAGUUCAGUGGCTT
SRC-1	CUCUAUGACUAUGAGUCUATT	UAGACUCAUAGUCAUAGAGTT
SRC-2	CAGUGUCUGACUUCGACAATT	UUGUCGAAGUCAGACACUGTT
TAB3-1	CCACCUCAACAGCCAUCUUTT	AAGAUGGCUGUUGAGGUGGTT
TAB3-2	GGUUGAAGUCUGAAGUUAATT	UUAACUUCAGACUUCAACCTT
TES-1	CGAACUGCACUUCUGGAGAAATT	UUUCUCCAGAAGUGCAGUUCGTT
TES-2	GCUGAUAUUCAGCAAUGAGUATT	UACUCAUUGCUGAAUAUCAGCTT
TLN1-1	ACAAGAUGGAUGAAUCAAAUUTT	AAUUUGAUUCAUCCAUCUUGUTT
TLN1-2	AGCAGAAGGGAGAGCGUAAUUTT	AAUUACGCUCUCCCUUCUGCUTT
WW45-1	GAAUUUGGAACCUAUUATT	AAUUGGAGCAGAUUGUUATT
WW45-2	AAUUGGAGCAGAUUGUUATT	UCAACUAUGAGGUCCUUAGTT
WWC1-1	GAGAUCCUGAAAGCUGAAAUUTT	AAUUUCAGCUUUCAGGAUCUCTT
WWC1-2	CAACCUUCUCAGCUACAAAUATT	UAUUUGUAGCUGAGAAGGUUGTT
ZO1-1	GGAUGUUUAUCGUCGCAUUTT	AAUGCGACGAUAAACAUCCTT
ZO1-2	CGAUCUCAUAAACUUCGUATT	UACGAAGUUUAUGAGAUCGTT
ZO2-1	CGUCAUCAGUAUUCUGAUUAUTT	AUAAUCAGAAUACUGAUGACGTT
ZO2-2	CGGUUAAAUACCGUGAGGCAATT	UUGCCUCACGGUAUUUAACCGTT
ZYX-1	GCCUCAGGUCCAACUCCAUTT	AUGGAGUUGGACCUGAGGCTT
ZYX-2	GGAUCUGGGUCACAACCAATT	UUGGUUGUGACCCAGAUCCTT

Supplementary Table 5. qRT-PCR primers

Species: h.—Human; m.—Mouse

qPCR primers	Forward primer sequence(5'-3')	Reverse primer sequence(5'-3')
h. *CTGF*	CTCCTGCAGGCTAGAGAAGC	GATGCACTTTTTGCCCTTCTT
h. *CYR61*	AAGAAACCCGGATTTGTGAG	GCTGCATTTCTTGCCCTTT
h.*18s rRNA*	GTAACCCGTTGAACCCCATT	CCATCCAATCGGTAGTAGCG
h.*GAPDH*	GCACCGTCAAGGCTGAGAAC	TGGTGAAGACGCCAGTGGA
h.*CTNNB1*	CATCTACACAGTTTGATGCTGCT	GCAGTTTTGTCAGTTCAGGGA
h.*CTNNA1*	GGGGATAAAATTGCGAAGGAGA	GTTGCCTCGCTTCACAGAAGA
h.*WWC1*	AGCTCCAAGTATGACCCTGAG	AAAGCCACGCTCTTTGAACTG
h.*FRMD6*	CCACCTCTTTGGACTCAGTGT	CAAATTGGTCGATACCCTTGCT
h.*NF2*	TTGCGAGATGAAGTGGAAAGG	CAAGAAGTGAAAGGTGACTGGTT
h.*PATJ*	GAGTGTAGCAGACAGGGATCA	TGCTTTTTGTGTGGACTGGTT
h.*AMOT*	AGGGCGAGATTCGGAGGAT	CCTCTGACCCCTCATATTCCTT
h.*ZO1*	CAACATACAGTGACGCTTCACA	CACTATTGACGTTTCCCCACTC
h.*ZO2*	GGGAAGGTCGCTGCTATTGT	CTCTCGCTGTAGCCACTCC
h.*SRC*	TGGCAAGATCACCAGACGG	GGCACCTTTCGTGGTCTCAC
h.*GAPDH*	GCACCGTCAAGGCTGAGAAC	TGGTGAAGACGCCAGTGGA
h.*CTNNB1*	CATCTACACAGTTTGATGCTGCT	GCAGTTTTGTCAGTTCAGGGA
h.*MARK1*	CCCCGGTGTAGAAACTCCATT	AGAACGTGTCTTGCCAATTTGA
h.*MARK2*	CACATTGGAAACTACCGGCTC	GGAGGAGTTCAGTTGAGTCTTGT
h.*MARK3*	CCTCCTGTGCAGATGAACAAC	GCCTGTAAGGATATGTCTTGCC
h.*MARK4*	TGAAGGGCCTAAACCACCC	CCAGCACTTGCGTACTCCA
h.*CNN2*	ACCGGCTCCTGTCCAAATATG	CCCGGCTGTAGCTTGTTCA
h.*ZYX*	TCTCCCGCGATCTCCGTTT	CCGGAAGGGATTCACTTTGGG
h.*MYO1E*	ACTCTGCACGCCATGAATGT	CTCCACAGCCGCGTAGTTG
h.*IQGAP3*	TGACCACTACCTAACTCAGGC	CAGGGCAGGGTCTTGAAGG
h.*OBSL1*	ACGAGCAGATCGAAGAGGG	CGCATCTCGCACAGGTAGATA
h.*ADD1*	ACTTCGACCGAGTAGATGAGAA	GCAGAATCATGGACACCCTCT
h.*CSK*	AGGACCCCAACTGGTACAAAG	CGTGGAACCAAGGCATGAG
h.*EPS15*	AGTTGTTGCAGCAAGCGATTC	GTGGCTGAACGAAAAGGATCT
h.*FHOD1*	GGGTCAACGCTATCTTGGAAA	CAGCCCCTCTGAATGCACAA
h.*FLNA*	CTTATCGCGCTGTTGGAGGT	GCCACCGACACGTTCTCAA
h.*KIF5B*	CTGGCCGAGTGCAACATCA	CGATCACGACCGTGTCTTCT
h.*LRRFIP1*	GCTATGGTTTCCAATGCTCAGC	GCCGCCTAGATTCAGCCAG
h.*PDLIM5*	AAGAATAGGCGATGTGGTTCTCA	GCAGCAGATGCTCTTTGCAG
h.*PHLDB2*	TGGTGCATTCTGTTGAGAACG	CAGGCACAGGTTGTGAGAG
h.*PKN2*	TGACCCTCGTTGTTCTACTAGC	GTTTCCGATCCTTTGAAGATCCA
h.*SH3GLB2*	GAGGCGGCTCCTCCAAAAC	CGAGGTCTAGTCTCCTGAAAGTC
h.*TBCD*	CACCAAGGTTCGAGGCTATAAAA	GCAAAAGCATGTAGCGGGTTT
h.*TES*	AGTGCCATGAGTTGTCTCCC	GGGTGCTTCTATCCCCTCCA
h.*TLN1*	GACGATGCAGTTTGAGCCG	GGGTCATCATCTGACAGAAAGAG
h.*TXNDC9*	CTGCTTCAGACTACCAAACTGG	CTCTGTAGAAATGGCAAACCACA

h.*IQGAP1*	TTTGTACCTGTTCAAGCTAGGC	CCACTGACAGTTCATTAGCCAAG
h.*DNAJB1*	CCAGTCACCCACGACCTTC	CCCTTCTTCACTTCGATGGTCA
h.*KLC2*	ACTGGTCTATCGGGATCAGAAC	CCAGGTTGTTTAGTGTCGCAG
h.*SCYL1*	ATCCCCGAGCTTGAGCAGTAT	GGCCCATTGAAGACTTCCCAA
h.*UNC45A*	TCTGAGCATCAGTCACGGACA	TGAGGGCATCAAACATAACCTG
h.*USP8*	AAGGAGCAATCACAGCAAAGG	CTGCATTCTTCGAGCATCCATTA
h.*RAB10*	CAAGGGAGCATGGTATTAGGTTT	CTAACGTGAGGAACGCCTTTT
h.*DIAPH3*	AGAGCGAGAAAGACTCGAACG	TTTTGGCATCGGTGTCCTTTT
h.*FASN*	AAGGACCTGTCTAGGTTTGATGC	TGGCTTCATAGGTGACTTCCA
h.*CC2D1A*	CATTGACGAAGCGGACATCC	GCTGACGCGATTCTAGGGG
h.*LIMD1*	GAGCCTGGCGACTTCTGAG	CCTGGTTTGTCACCCCACTTT
h.*FLNC*	GCTCGTGTCCATAGACAGCAA	CTGGGGCACCTTGTTCTGG
h.*FLNB*	AACTGGCAAGACGGCAAAG	CGTGCATTATCCACAGGCTTC
h.*C1QBP*	ATCAACTCCCAATTTCGTGGTT	GGTGGTCATATAAGGCCCAGT
h.*HSPD1*	GTGTAGACCTTTTAGCCGATGC	GTGCCAGTACAGTAGCAGTGG
h.*HSPE1*	CGCTGTTGGATCGGGTTCTAA	AACTTTCACGCTAACTGGTTGA
m. *Ctgf*	CCAATGACAACGCCTCCTG	TGGTGCAGCCAGAAAGCTC
m. *Cyr61*	AGCCTCGCATCCTATACAACC	TTCTTTCACAAGGCGGCACTC
m.*18s rRNA*	TTCGAACGTCTGCCCTATCAA	ATGGTAGGCACGGCGACTA
m.*Hes1*	CCAGCCAGTGTCAACACGA	AATGCCGGGAGCTATCTTTCT
m.*Hey1*	CCGACGAGACCGAATCAATAAC	TCAGGTGATCCACAGTCATCTG
m.*Gapdh*	CGTCCCGTAGACAAAATGGT	GAATTTGCCGTGAGTGGAGT

Figure S1

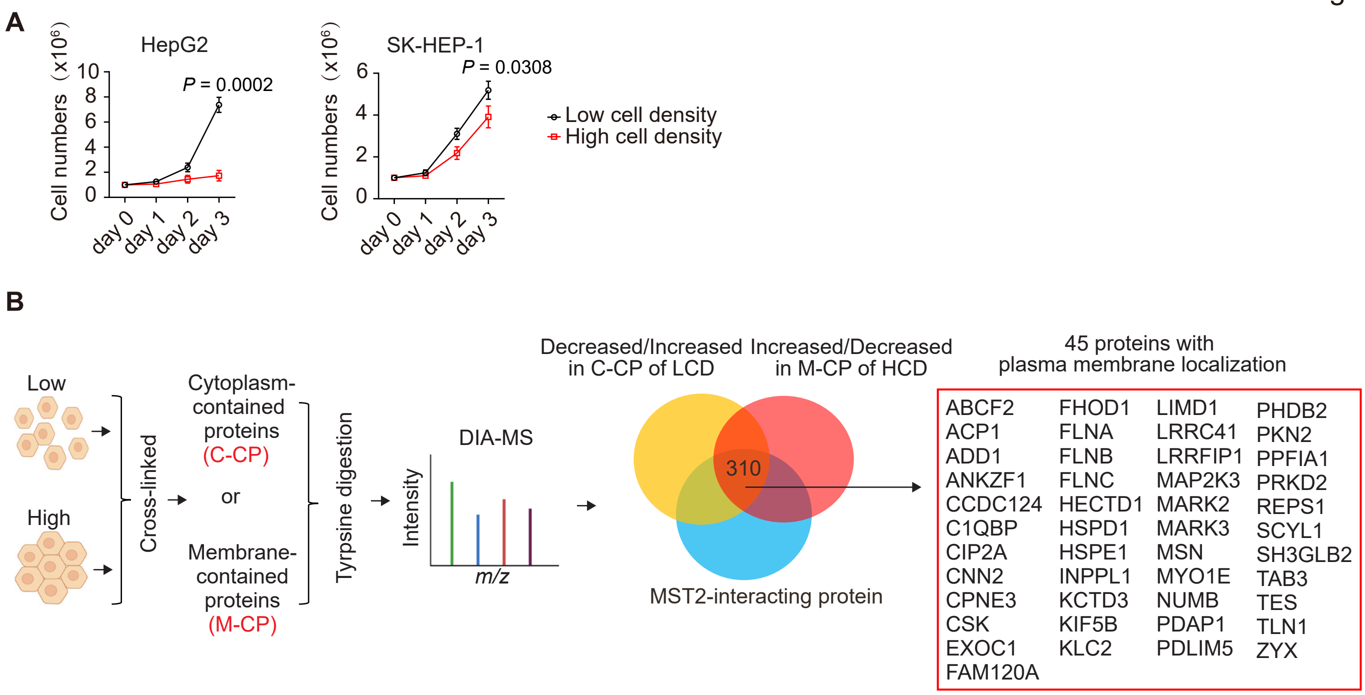

Figure S1 (Related to Figure 1). NUMB sense cell density to modulate YAP activity. (A) Growth curve assays of HepG2 or SK-HEP-1 cells cultured at low cell density (LCD) or high cell density (HCD). Data represent the mean ± SD from experimental triplicate experiments and the *P* values of two-tailed unpaired Student's t test. (B) Schematic diagram of cell density sensing proteins screening for Hippo/MST-YAP signaling regulation. The fold change of decreased or increased in C-CP of LCD than of HCD, and increased or decreased in M-CP of HCD than of LCD were > 1.5.

Figure S2

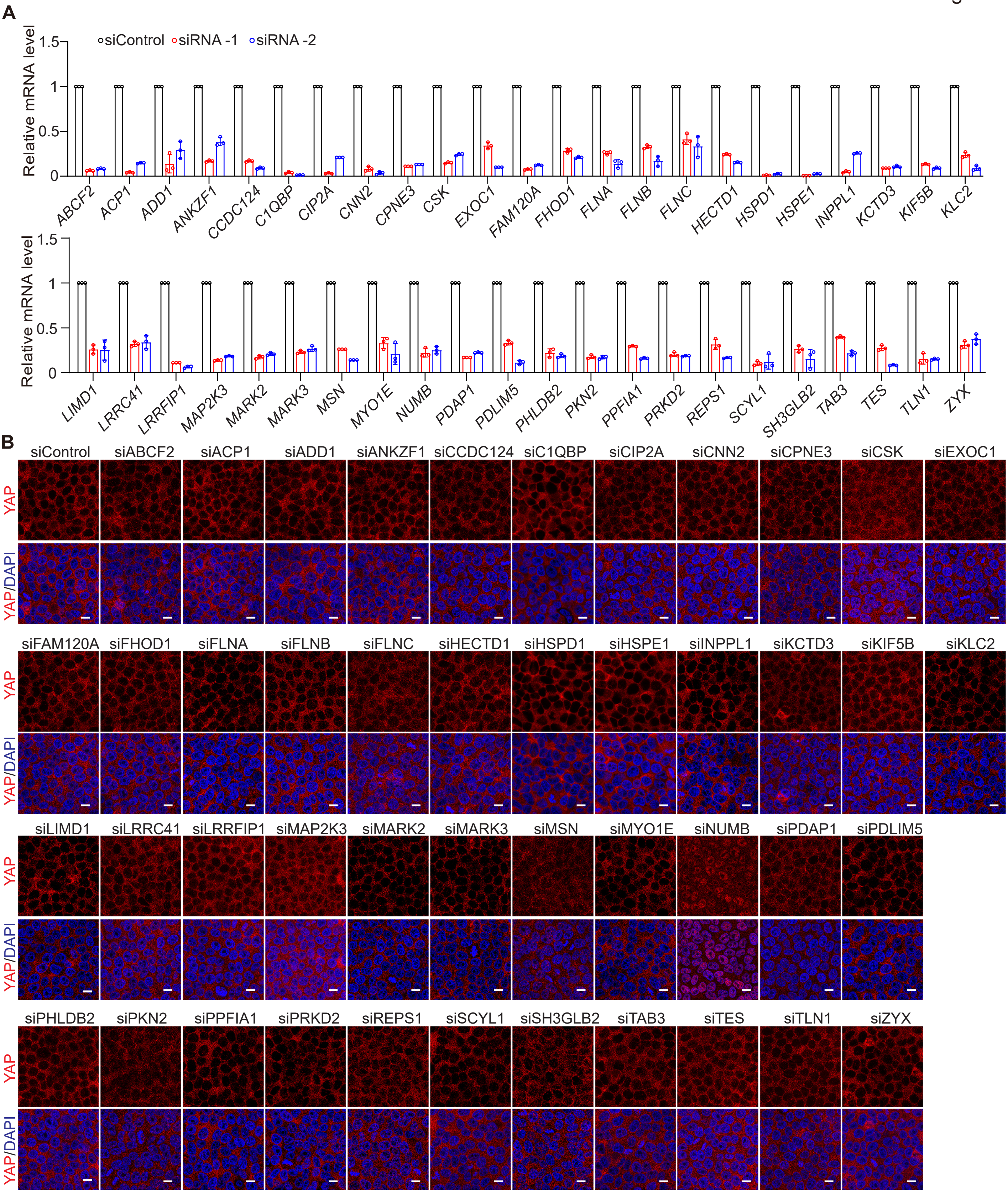

Figure S2 (Related to Figure 1 and S1). NUMB sense cell density to modulate YAP activity. (A) Knockdown efficiency of indicated siRNAs was analyzed by qPCR. Each bar represents the mean ± SD from experimental triplicate experiments. (B) Immunofluorescence staining of YAP (red) and DAPI (blue) in HepG2 cells transfected with indicated siRNAs and cultured at HCD. Scale bars, 10 μm.

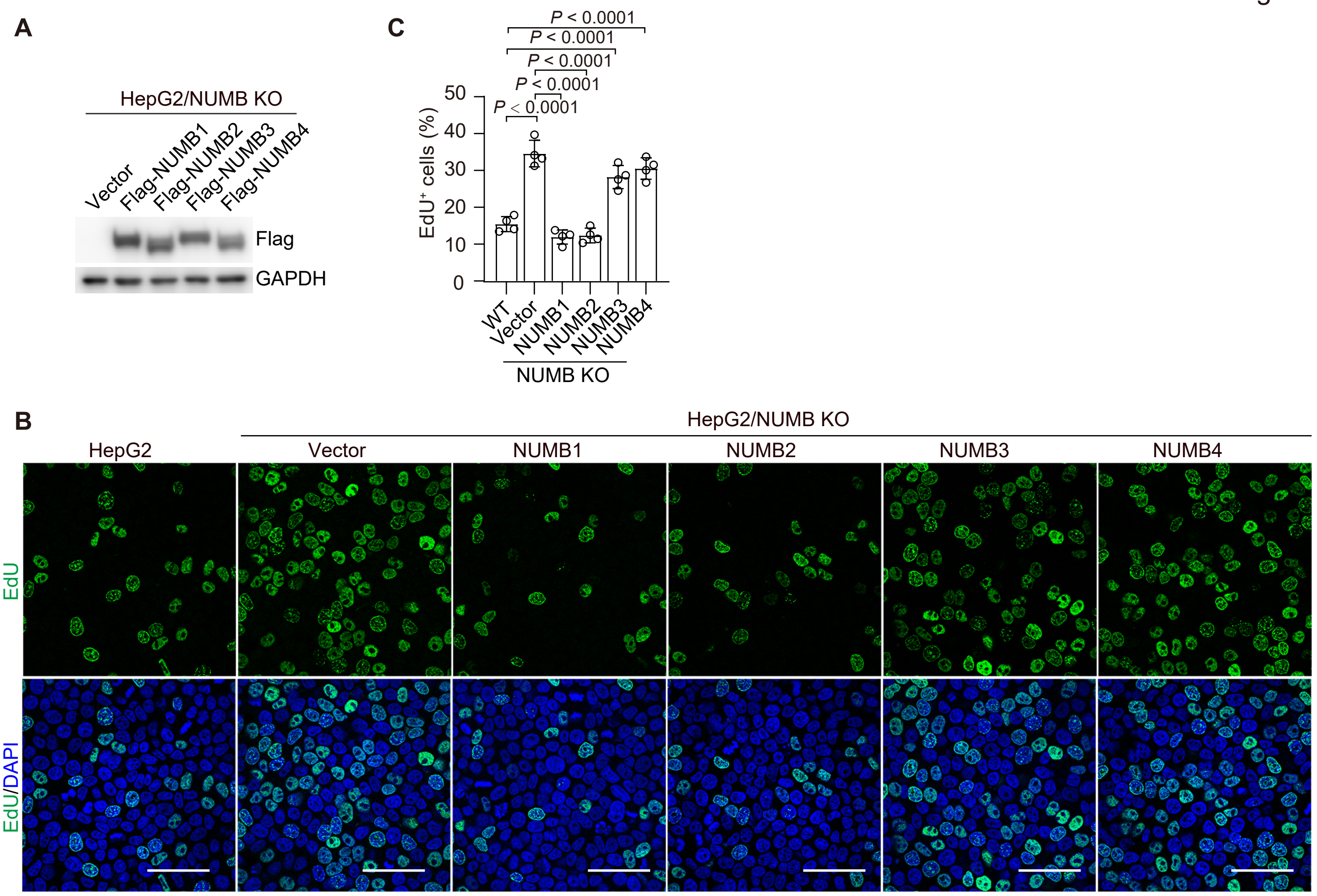

Figure S3 (Related to Figure 2). NUMB1/2 repress the cell proliferation of NUMB KO HepG2 cells. (A) Immunoblot analysis of NUMB KO HepG2 cells stably expressing vector or Flag-tagged NUMB1-4. (B-C) Immunofluorescence staining of EdU (green) and DAPI (blue) (B) and the percentage of EdU positive cells (C) of WT and NUMB KO HepG2 cells expressing vector, Flag-tagged NUMB1-4 cultured at HCD. Scale bar, 50 µm. Data are presented as the mean ± SD from biological triplicate experiments and the *P* value was assessed using one-way ANOVA followed by Tukey's multiple comparisons test.

Figure S4

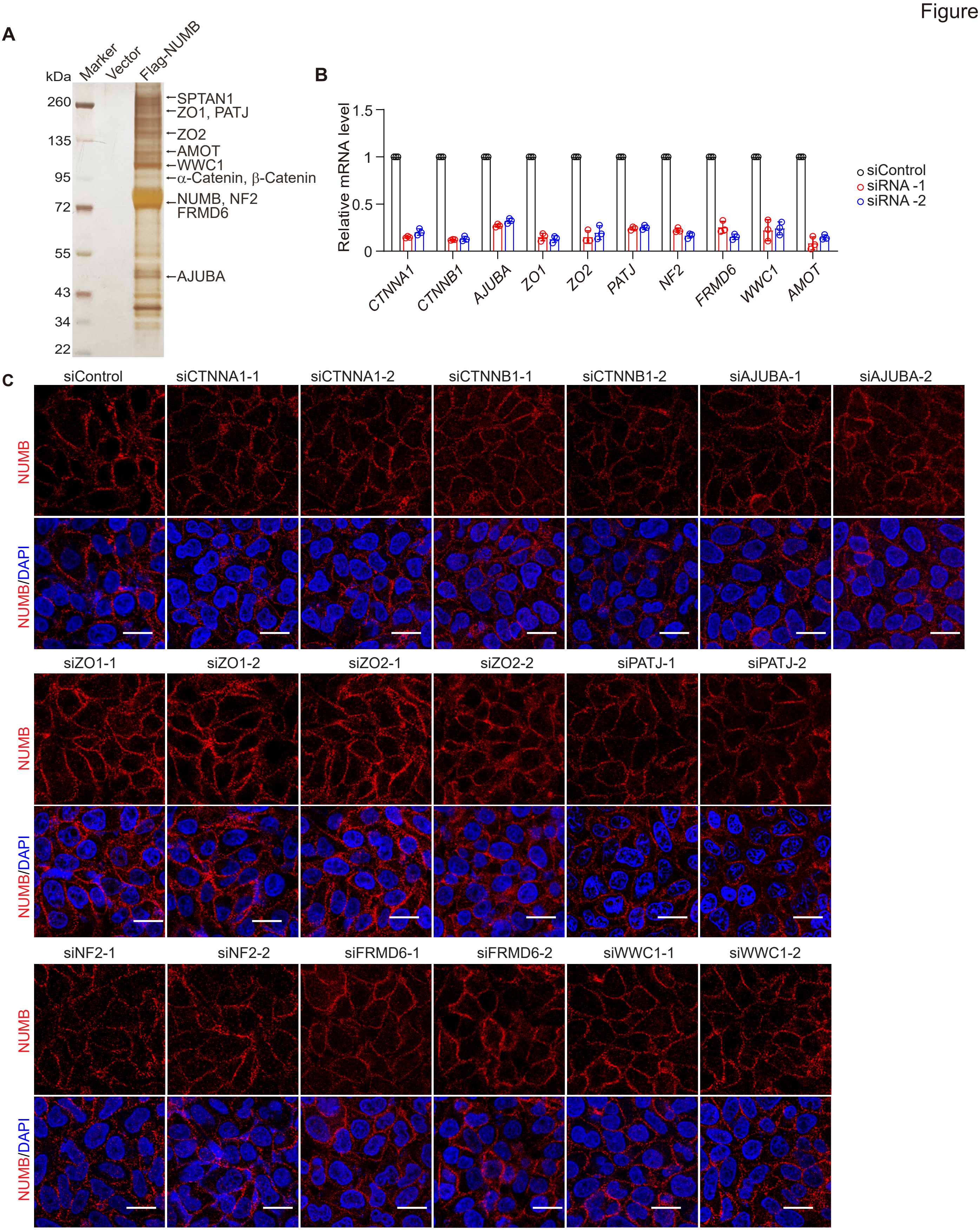

Figure S4 (Related to Figure 3). SPTAN1 is required for NUMB membrane retention at HCD. (A) Silver stain of SDS-PAGE of purified NUMB-containing protein complexes. (B) Knockdown efficiency of indicated siRNAs was analyzed by qPCR. Each bar represents the mean ± SD from experimental triplicate experiments. (C) Immunofluorescence staining of NUMB (red) and DAPI (blue) in HepG2 cells transfected with indicated siRNAs and cultured at HCD. Scale bars, 20 μm.

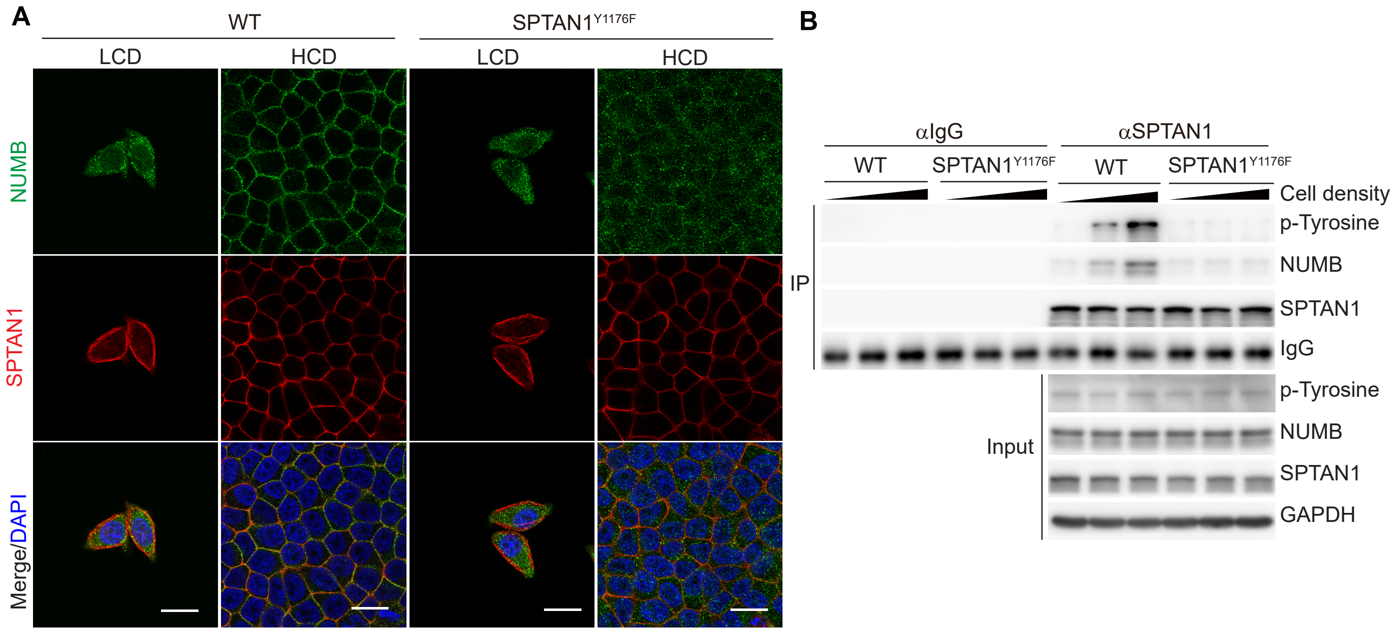

Figure S5 (Related to Figure 4). High cell density induces SPTAN1 phosphorylation for NUMB1/2 membrane retention. (A) Immunofluorescence staining of NUMB (green), SPTAN1 (red) and DAPI (blue) in WT or SPTAN1 Y1176F mutant HepG2 cells cultured at LCD or HCD. Scale bars, 20 μm. (B) Whole-cell lysates from WT and SPTAN1 Y1176F HepG2 cells cultured at different cell densities were collected for co-IP analysis.

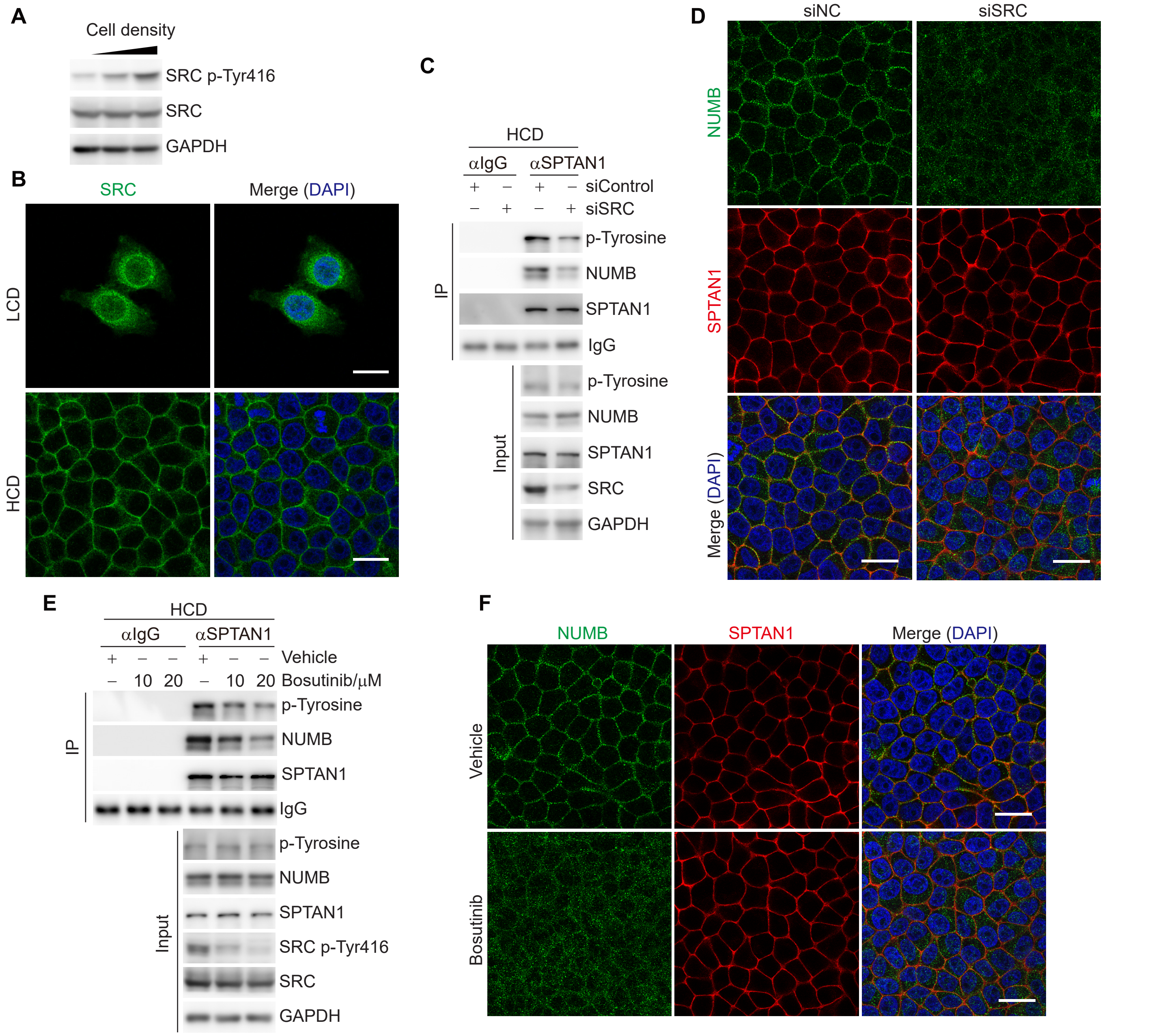

Figure S6 (Related to Figure 4). SRC promotes SPTAN1 phosphorylation for NUMB1/2 membrane retention. (A) Immunoblot analysis of p-SRC, SRC and GAPDH in HepG2 cells cultured at different cell densities. (B) Immunofluorescence staining of SRC (green) and DAPI (blue) in HepG2 cells cultured at LCD or HCD. Scale bars, 20 μm. (C) Whole-cell lysates from HepG2 cells co-transfected with indicated siRNAs and cultured at HCD were collected for co-IP analysis. (D) Immunofluorescence staining of NUMB (green), SPTAN1 (red) and DAPI (blue) in HepG2 cells transfected with indicated siRNAs and cultured at HCD. Scale bars, 20 μm. (E) Whole-cell lysates from HepG2 cells treated with vehicle or Bosutinib for 4h and cultured at HCD were collected for co-IP analysis. (F) Immunofluorescence staining of NUMB (green), SPTAN1 (red) and DAPI (blue) in HepG2 cells treated with vehicle or 20 μM Bosutinib for 4h and cultured at HCD. Scale bars, 20 μm.

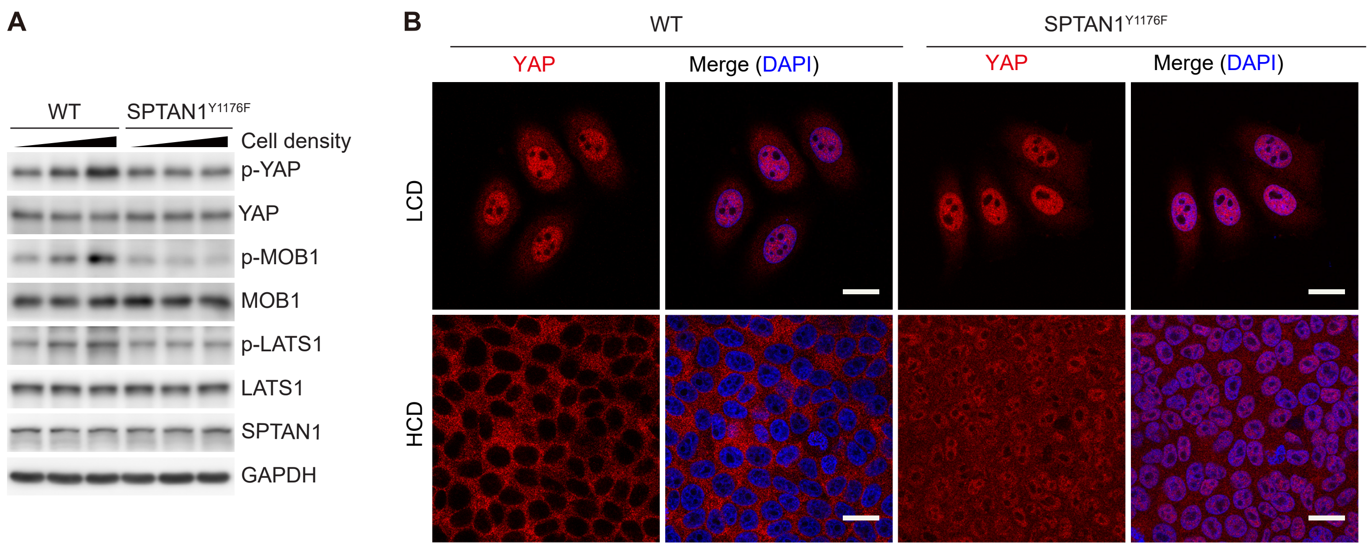

Figure S7 (Related to Figure 4). SPTAN1 phosphorylation is required for Yap in activation. (A-B) Immunoblot analysis of indicated proteins (A), and immunofluorescence staining of YAP (red) and DAPI (blue) (B) in WT and SPTAN1 Y1176F mutant HepG2 cells cultured at different cell densities. Scale bars, 20 μm.

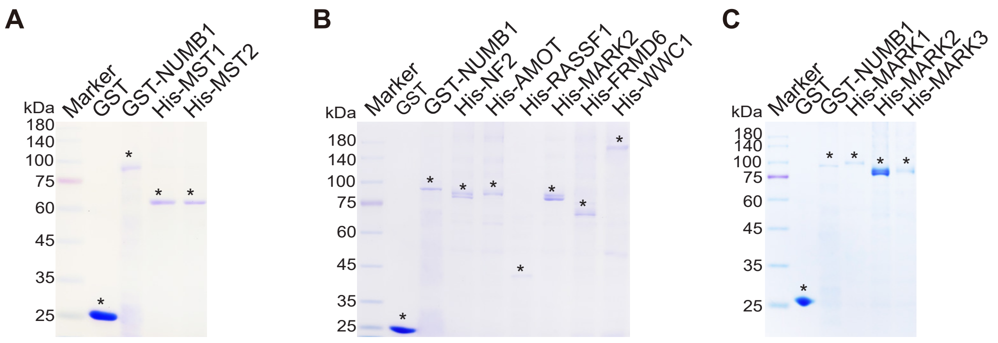

Figure S8 (Related to Figure 5). NUMB1/2 sequestrate MARK on the plasma membrane at high cell density. (A-C) Commassie Blue staining of indicated recombinant proteins (marked by asterisks).

Figure S9

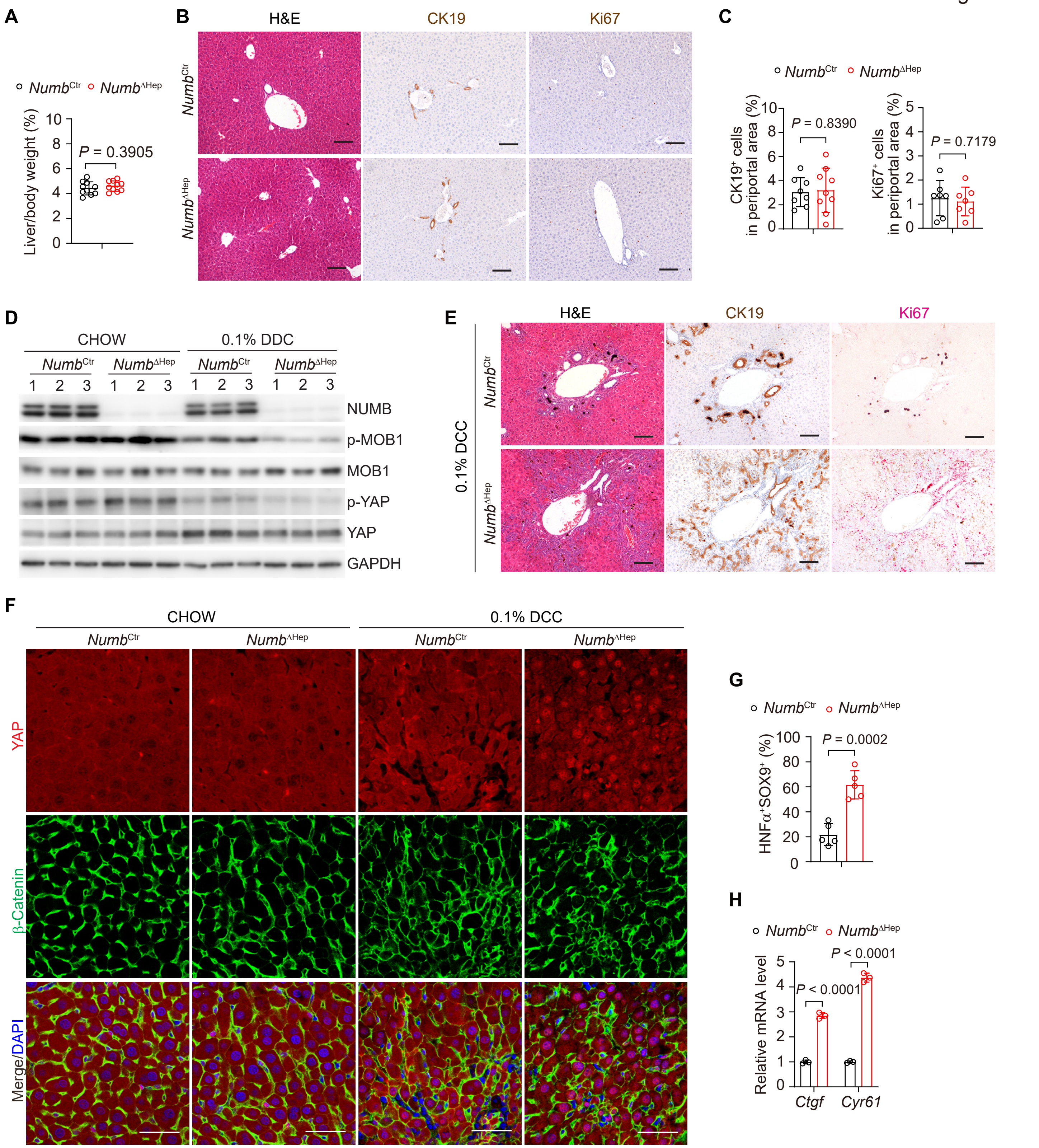

Figure S9 (Related to Figure 7). NUMB deficiency promotes Yap activation in the liver with DDC treatment. (A-C) The liver-to-body weight ratios (n=10, 10) (A), H&E staining and IHC staining of CK19 and Ki67 (B), and the percentage of CK19 or Ki67 positive cells in the liver periportal areas (C) of *Numb*^{Ctr} and *Numb*$^{\Delta Hep}$ mice at 3 months old. Scale bars, 50 μm. (D) Immunoblot analysis of primary hepatocytes lysates of *Numb*^{Ctr} and *Numb*$^{\Delta Hep}$ mice treated with chow or DDC. (E) H&E staining and IHC staining of CK19 and Ki67 of *Numb*^{Ctr} and *Numb*$^{\Delta Hep}$ mice treated with 0.1% DDC. Scale bars, 50 μm. (F) Immunofluorescence staining of YAP (red) and β-Catenin (green) in the liver sections of *Numb*^{Ctr} and *Numb*$^{\Delta Hep}$ mice treated with chow or DDC. Scale bars, 50 μm. (G and H) The percentage of SOX9 and HNF4α double positive cells (G), and qPCR analysis (H) in CLiPs (Chemically Induced Liver Progenitors) derived from primary hepatocytes of *Numb*^{Ctr} and *Numb*$^{\Delta Hep}$ mice. Data are presented as mean ± SD and the *P* value of two-tailed unpaired Student's t test in A, C, G and H.

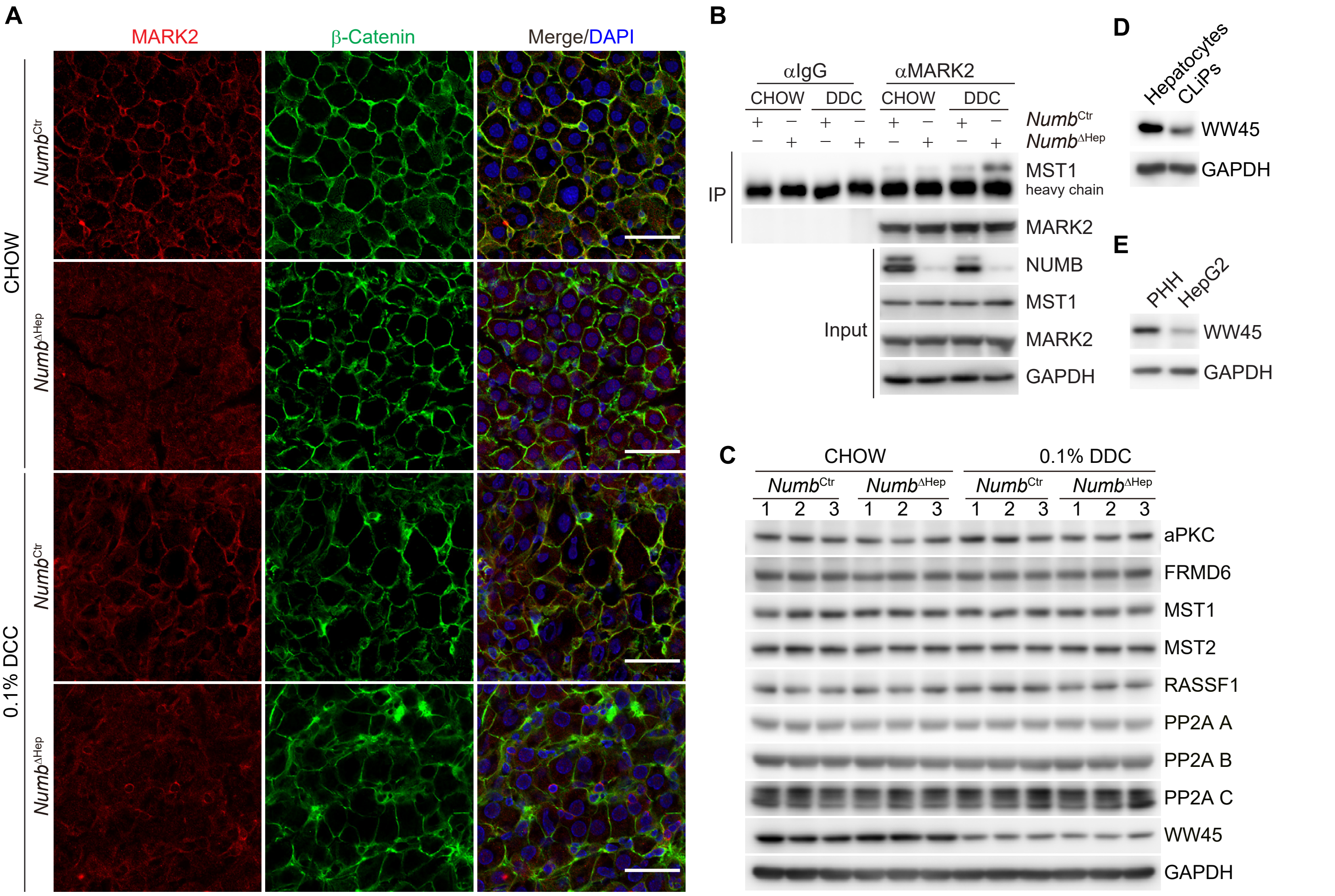

Figure S10 (Related to Figure 8). WW45 competes with MARK to modulate Hippo kinase MST1/2 activity. (A) Immunofluorescence staining MARK2 (red) and β-Catenin (green) in the liver sections of *Numb*^{Ctr} and *Numb*$^{\Delta Hep}$ mice treated with chow or DDC. Scale bars, 25 µm. (B) Whole-cell lysates of hepatocytes isolated from mice were collected for co-IP analysis. (C) Immunoblot analysis of indicated proteins in the primary hepatocytes of *Numb*^{Ctr} and *Numb*$^{\Delta Hep}$ mice treated with with chow or DDC. (D and E) Immunoblot analysis of WW45 and GAPDH in primary or CLiPs treated mouse hepatocytes (D) or in PHH or HepG2 cells (E).

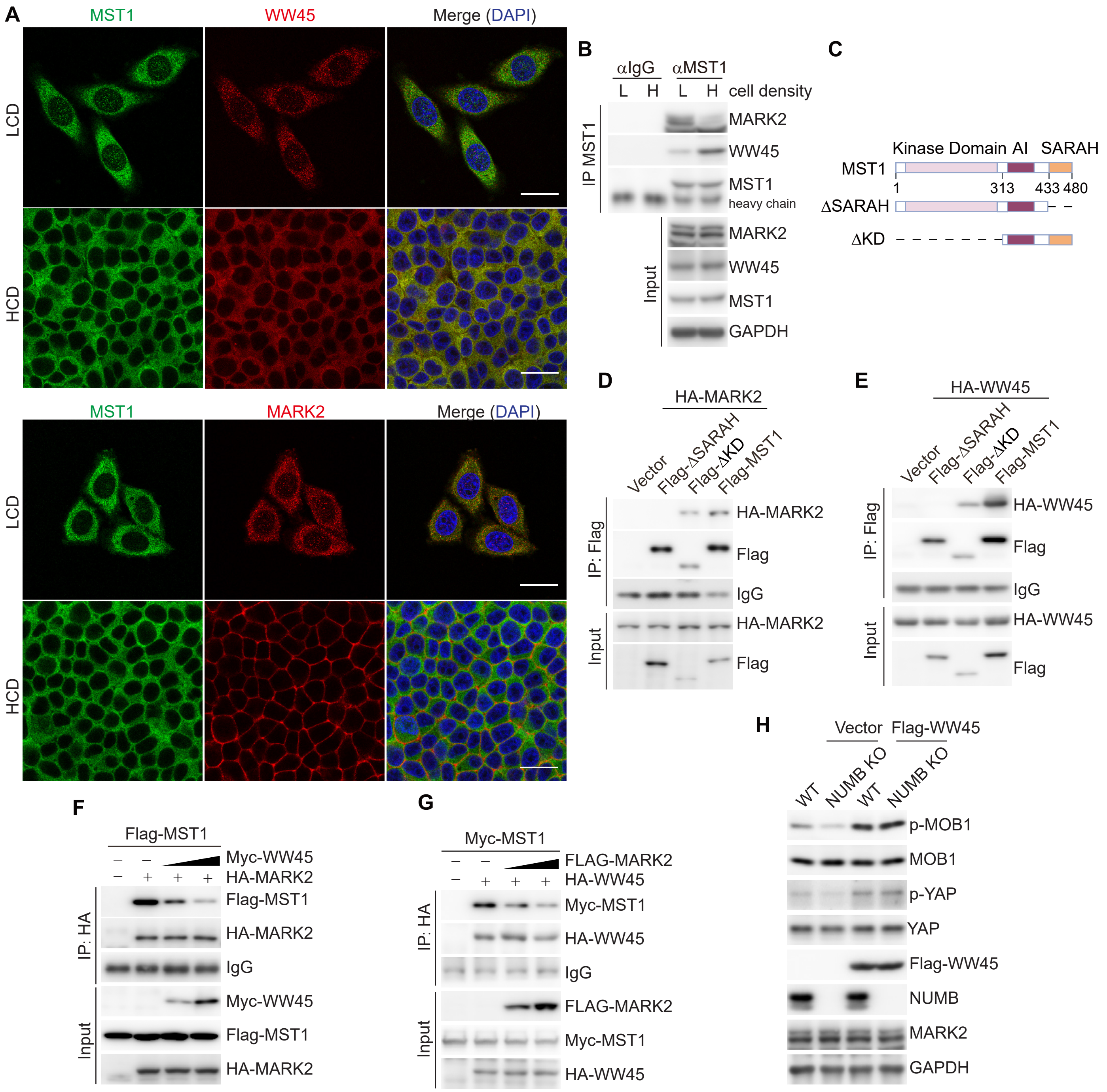

Figure S11 (Related to Figure 8 and S10). WW45 competes with MARK to modulate Hippo kinase MST1/2 activity. (A) Immunofluorescence staining of MST1 (green), WW45 (red) or MARK2 (red) and DAPI (blue) as indicated in HepG2 cells cultured at LCD and HCD. Scale bars, 20 µm. (B) Whole-cell lysates from HepG2 cells cultured at LCD and HCD were collected for co-IP analysis. (C) Diagram of the structures of full length or truncated MST1. AI, auto-inhibitory domain; SARAH, Salvador-RASSF-Hippo domain. (D-G) Whole-cell lysates from HEK293T cells co-transfected with indicated constructs were collected for co-IP analysis as indicated. (H) Immunoblot analysis of indicated proteins in WT and NUMB KO HepG2 cells expressing vector or Flag-tagged WW45.

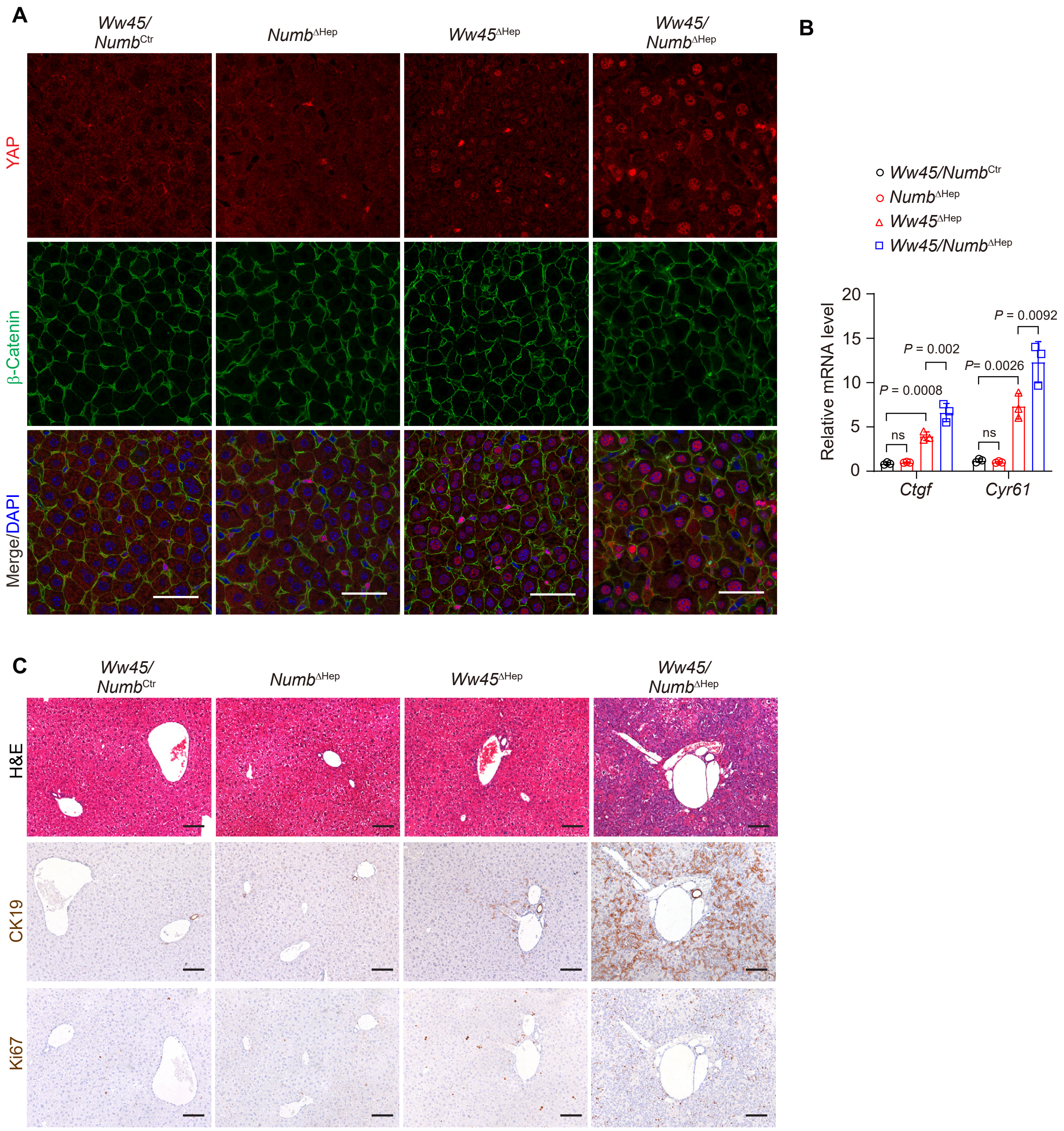

Figure S12 (Related to Figure 8). NUMB interfaces with WW45 to regulate YAP activity. (A-C) Immunofluorescence staining of YAP (red),β-Catenin (green) and DAPI (blue) (A), qPCR analysis of *Ctgf* and *Cyr61* (B), and H&E and IHC staining (C) in liver of *Ww45/Numb*^{Ctr}, *Numb*$^{\Delta\text{Hep}}$, *Ww45*$^{\Delta\text{Hep}}$ and *Ww45/Numb*$^{\Delta\text{Hep}}$ mice at 3 months old. Data are presented as mean ± SD and the *P* value was assessed using one-way ANOVA followed by Tukey's multiple comparisons test, Scale bars, 50 μm.

Figure S13

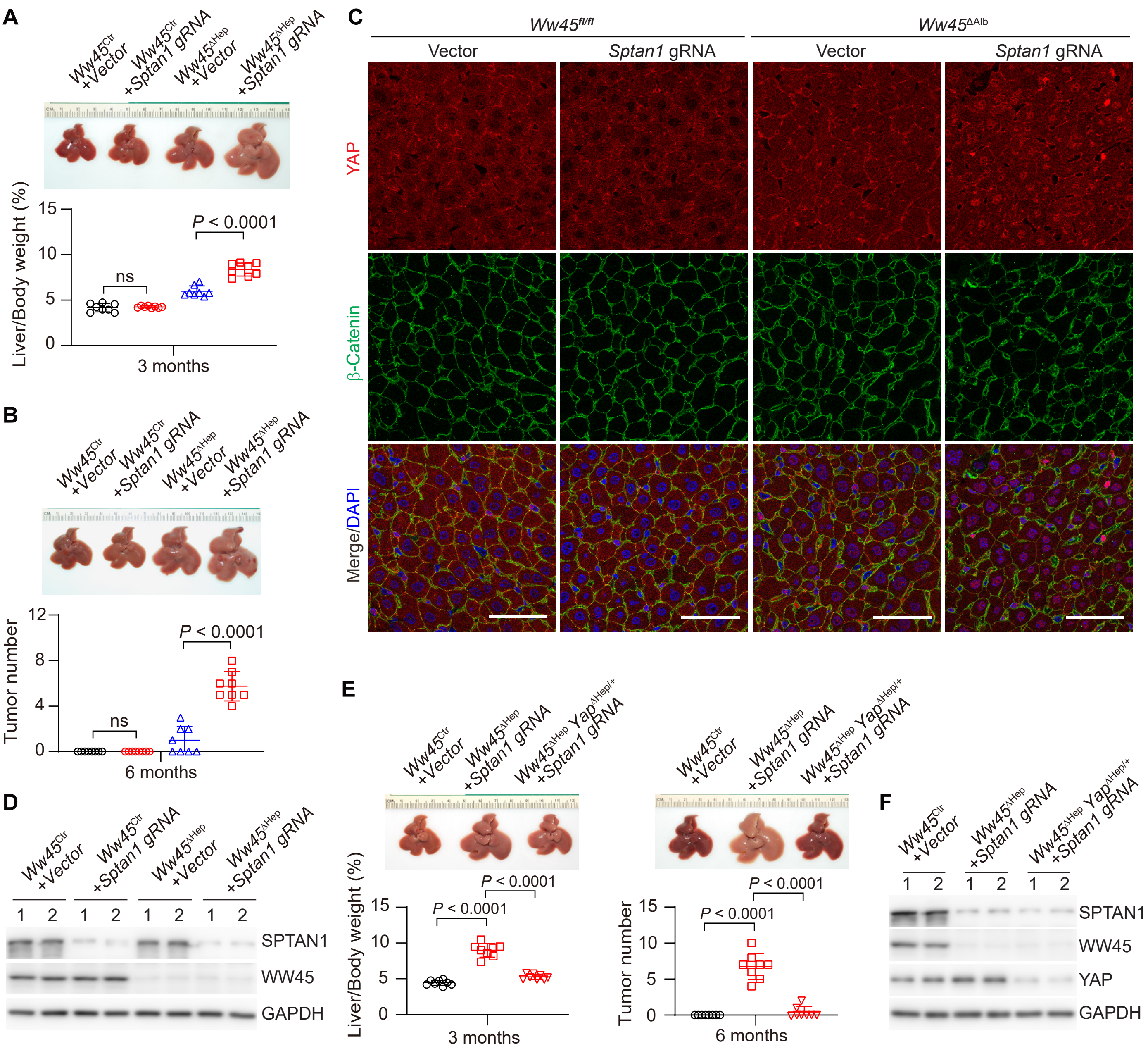

Figure S13 (Related to Figure 8). SPTAN1 interfaces with WW45 to restrain liver size and tumorigenesis. (A and B) Representative liver images and the liver-to-body weight ratios (3 months old, n=8, 8, 8, 8) (A), or the tumor numbers (6 months old, n=8, 8, 8, 8) (B) of *Ww45*^{Ctr} and *Ww45*$^{\Delta Hep}$ mice treated with *Sptan1* gRNA or control vector. (C and D) Immunofluorescence staining of YAP (red), β-Catenin (green) and DAPI (blue) (C), and immunoblot analysis of indicated proteins (D) in the liver of mice *Ww45*^{Ctr} and *Ww45*$^{\Delta Hep}$ mice treated with *Sptan1* gRNA or control vector at 3 months old. Scale bars, 50 µm. (E) Representative liver images, the liver-to-body weight ratios (3 months old, n=8, 8, 8, 8) and tumor numbers (6 months old, n=8, 8, 8) of *Ww45*^{Ctr} and *Ww45*$^{\Delta Hep}$ mice treated with *Sptan1* gRNA or control vector. (F) Immunoblot analysis of indicated proteins in the livers of *Ww45*^{Ctr}, *Ww45*$^{\Delta Hep}$ or *Ww45*$^{\Delta Hep}$*Yap*$^{\Delta hep/+}$ mice treated with *Sptan1* gRNA or control vector mice at 3 months old. Data are presented as mean ± SD, the *P* value was assessed using two-tailed unpaired Student's t test in A, B and one-way ANOVA followed by Tukey's multiple comparisons test in E.

Figure S14

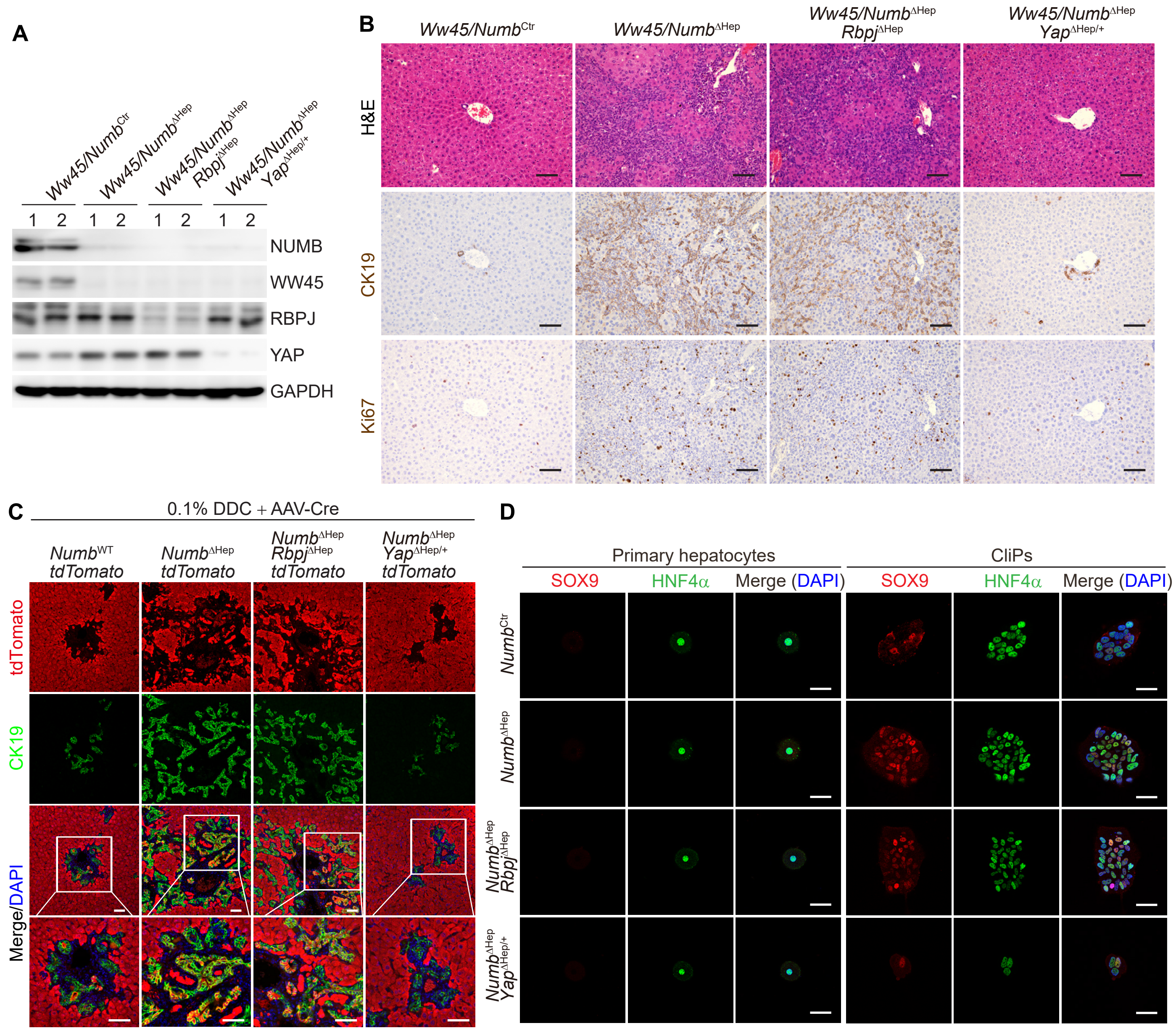

Figure S14 (Related to Figure 9). NUMB interfaces with WW45 to restrain liver dedifferentiation and tumorigenesis in a YAP- but not RBP-J-dependent manner. (A and B) Immunoblot analysis of indicated proteins (A), and the H&E and IHC staining of CK19 and Ki67 (B) in the liver sections of *Ww45/Numb*^{Ctr}, *Ww45/Numb*$^{\Delta Hep}$, *Ww45/Numb*$^{\Delta Hep}$*Rbpj*$^{\Delta Hep}$ and *Ww45/Numb*$^{\Delta Hep}$*Yap*$^{\Delta Hep/+}$ mice 3 months old. Scale bars, 50 µm. (C) Immunofluorescence staining of tdTomato (red), CK19 (green) and DAPI (blue) in the liver sections of *Ww45/Numb*^{Ctr}, *Ww45/Numb*$^{\Delta Hep}$, *Ww45/Numb*$^{\Delta Hep}$*Rbpj*$^{\Delta Hep}$ and *Ww45/Numb*$^{\Delta Hep}$*Yap*$^{\Delta Hep/+}$ mice (with tdTomato labeled hepatocytes) treated with AAV-Cre and DDC. Scale bars, 50 µm. (D) Immunofluorescence staining of SOX9 (red), HNF4α (green) and DAPI (blue) in primary and CLiPs treated mouse hepatocytes from indicated mice. Scale bars, 50 µm.

Figure S15

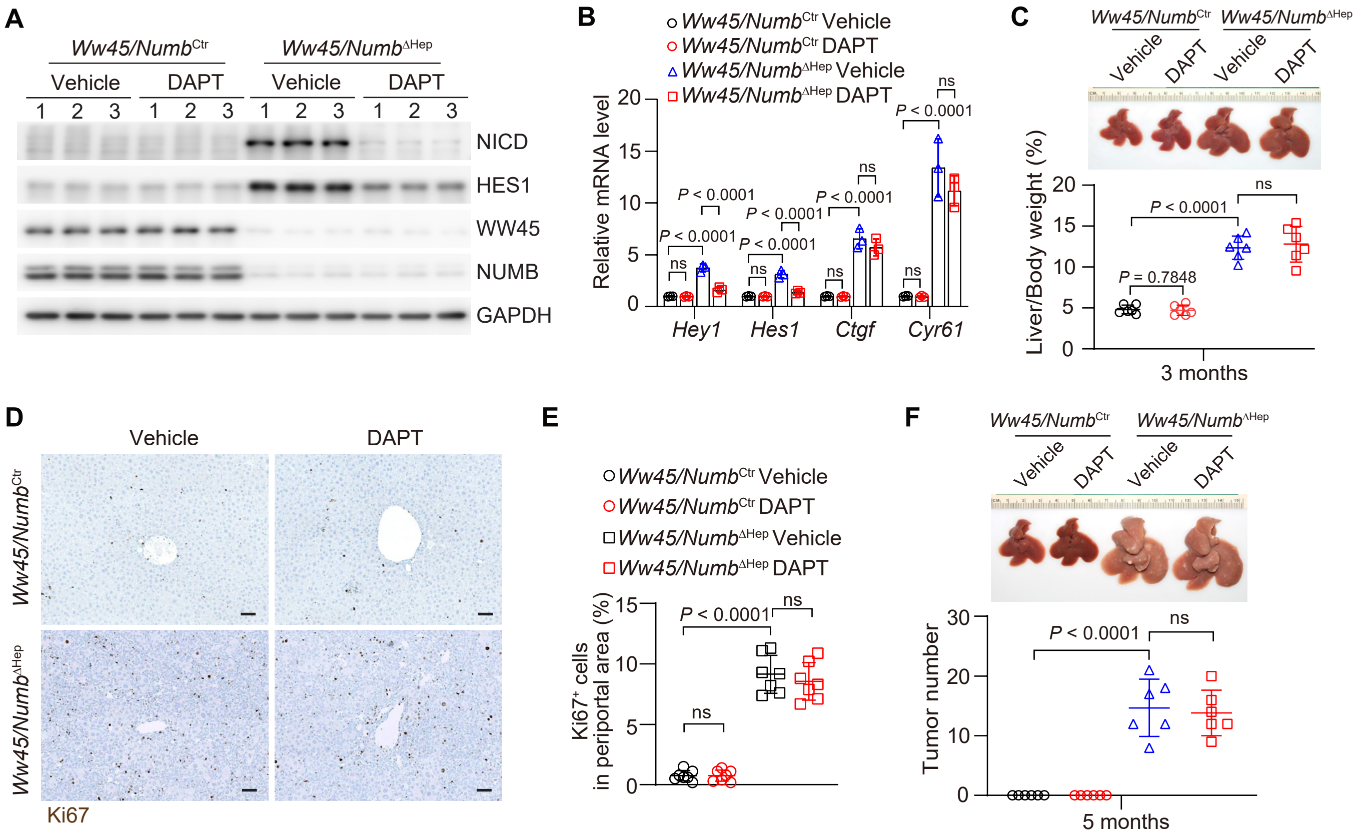

Figure S15 (Related to Figure 9). NUMB interfaces with WW45 to restrain liver size and tumorigenesis in a NICD-independent manner. (A-E) Immunoblot analysis of indicated proteins (A), qPCR analysis of indicated genes (B), representative liver images and the liver-to-body weight ratios (n=6, 6, 6, 6) (C), Ki67 staining (D) and the percentage of Ki67 positive cells (E) in the livers of *Ww45/Numb*^{Ctr} and *Ww45/Numb*$^{\Delta Hep}$ mice treated with vehicle or DAPT for 1.5 months. Scale bars, 50 µm. (F) Representative liver images and tumor numbers (n=6, 6, 6, 6) of indicated mice treated with vehicle or DAPT for 3.5 months. Data are presented as mean ± SD and the *P* value was assessed using one-way ANOVA followed by Tukey's multiple comparisons test in B,C, E and F.

Figure S16

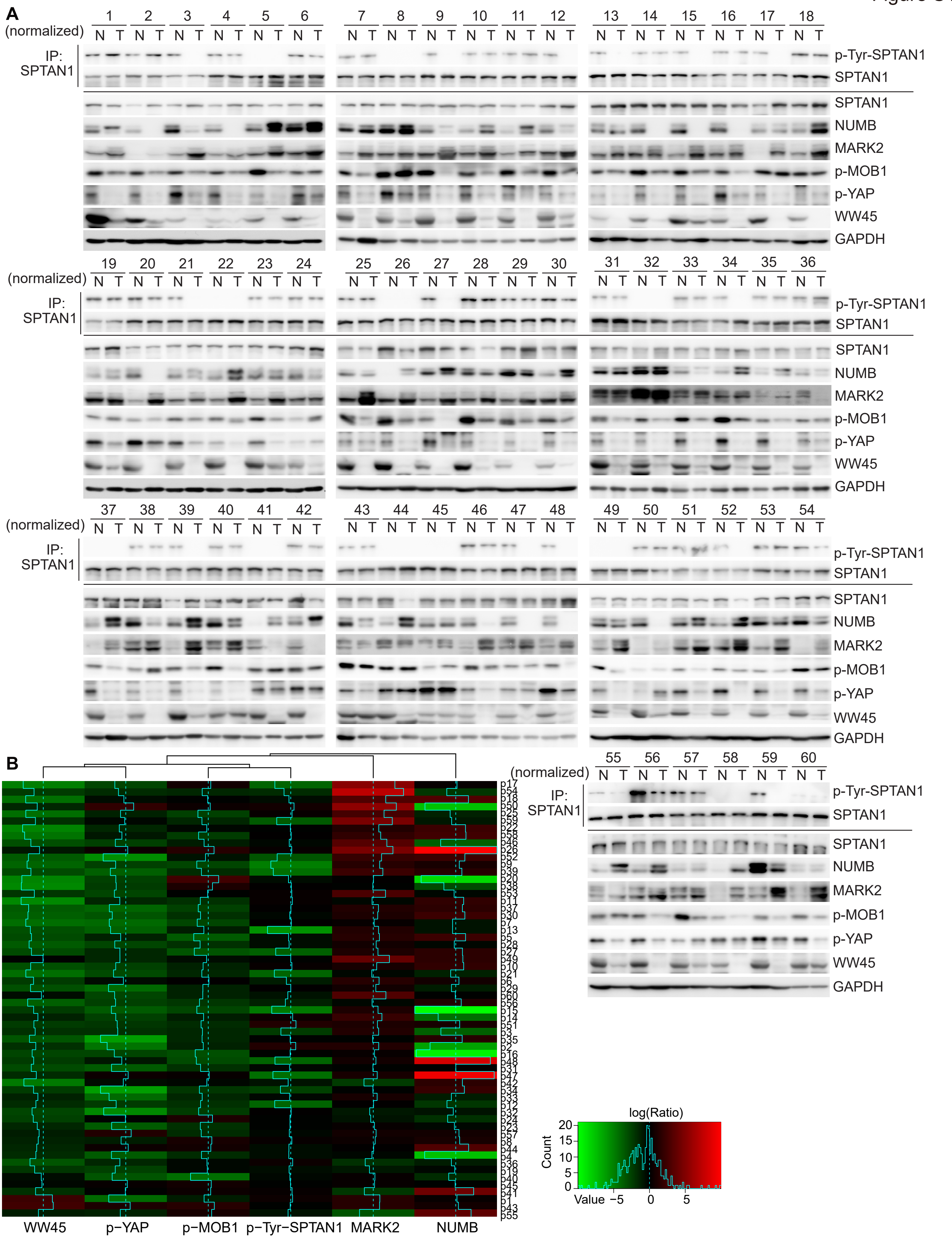

Figure S16 (Related to Figure 10). The analysis of SPTAN1/NUMB-MARK/Hippo signaling in human liver cancer. (A and B) Immunoblot analysis (A) and a heatmap representation (B) of the ratios of the relative expression of the indicated proteins in the T and N samples from one patient. For the detection of p-Tyr of SPTAN1, the loading of immunoprecipitates was normalized according to the levels of total SPTAN1. The intensities of the immunoblot bands were quantified using ImageJ software. Clustering was performed by using Pearson correlation metric and centroid linkage.

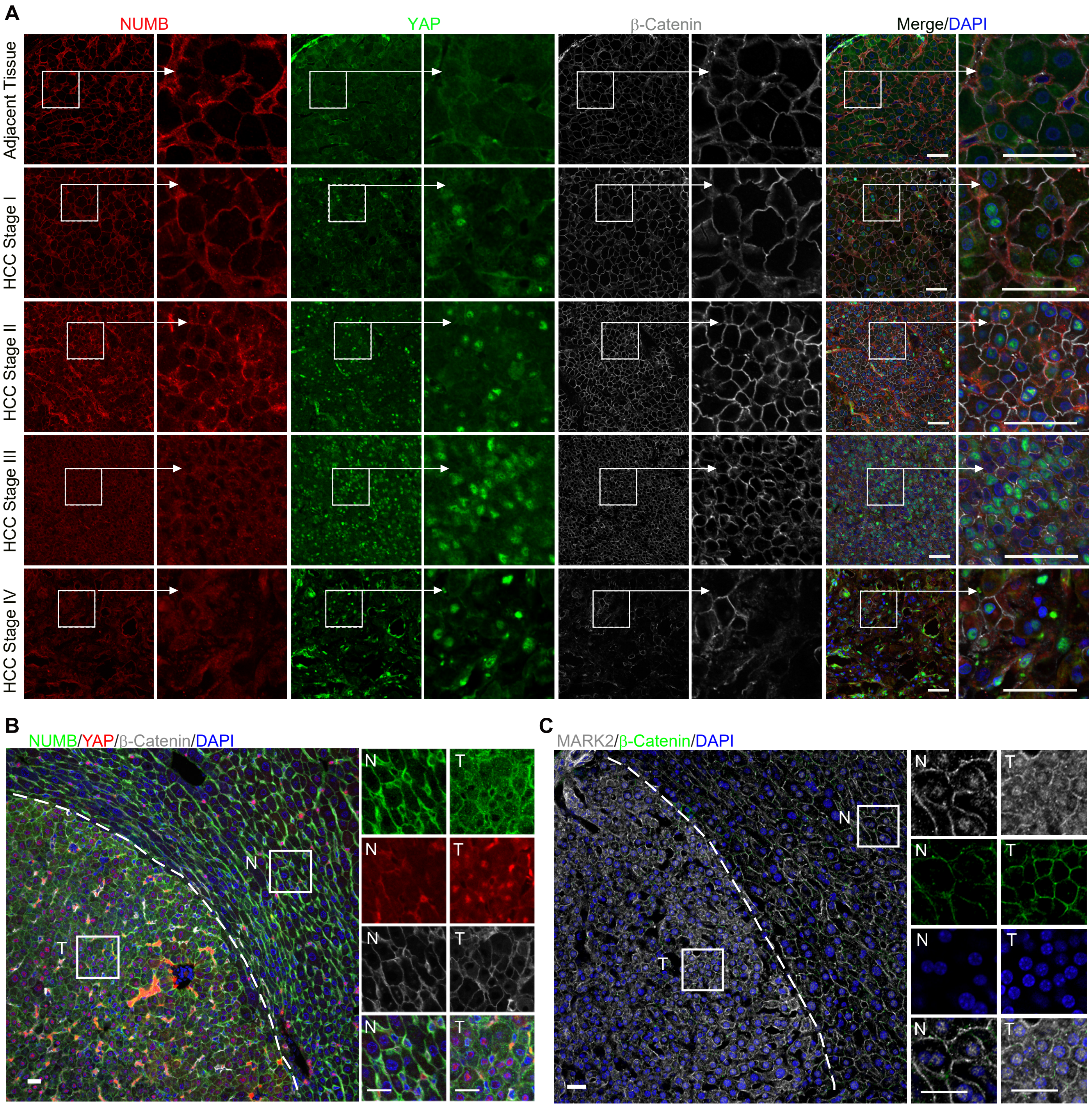

Figure S17 (Related to Figure 10). NUMB3/4 isoforms that fail to activate MST1/2 are preferably expressed in human liver cancer. (A) Immunofluorescence staining of NUMB (red), YAP (green), β-Catenin (gray) and DAPI (blue) in sections of non-tumorous tissues and HCC tumor tissues of the different stages. Scale bars, 50 μm. (B and C) Immunofluorescence staining of NUMB (red), YAP (green), β-Catenin (gray) and DAPI (blue) in liver sections from WT mouse treated with DEN for 8 months. Scale bars, 25 μm.

Figure S18

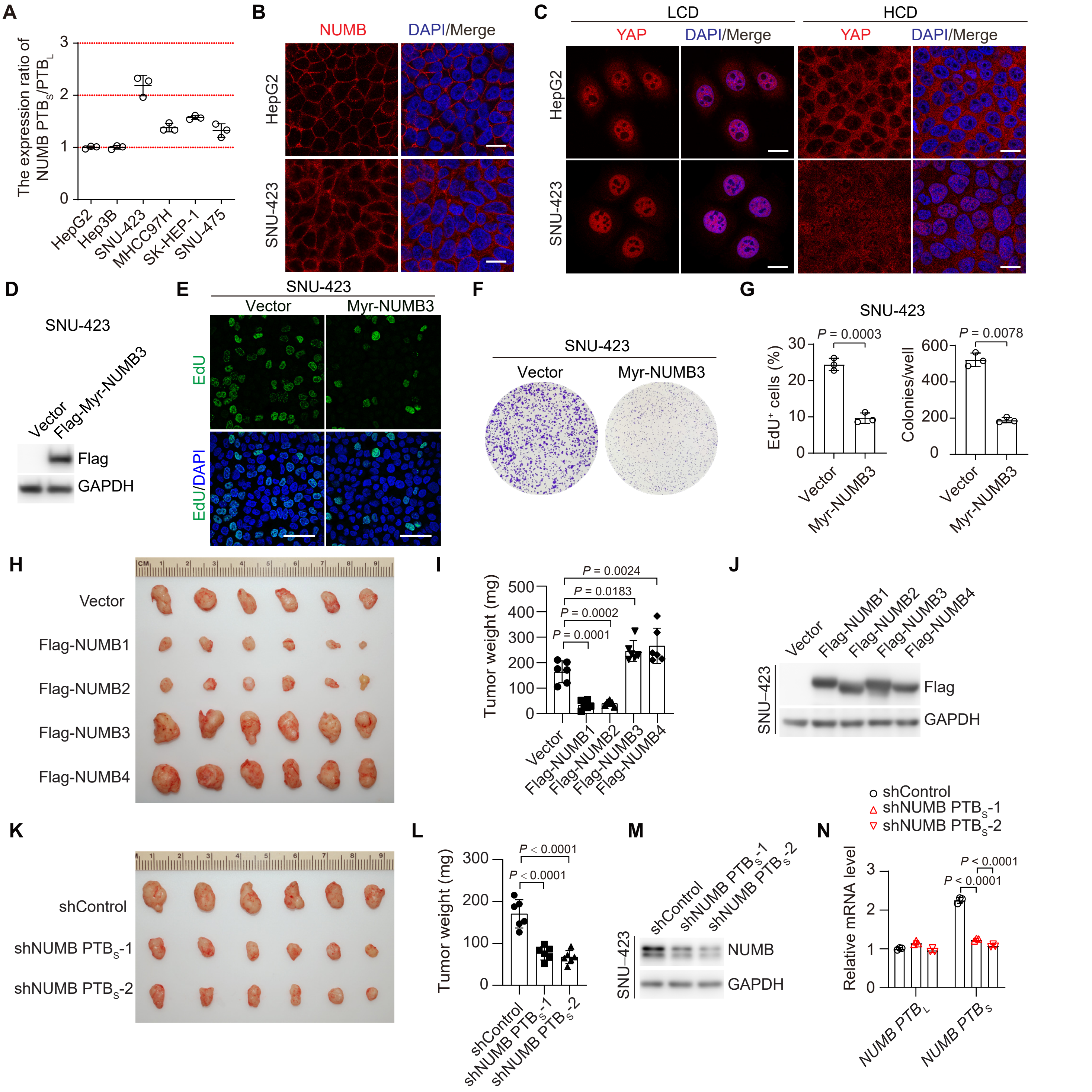

Figure S18 (Related to Figure 10). NUMB3/4 isoforms promote HCC progression. (A) The ratio of relative expression levels of NUMB-PTBs versus NUMB-PTBL in indicated liver cancer cell lines. Each bar represents the mean ± SD from experimental triplicate experiments. (B and C) Immunofluorescence staining of NUMB in HepG2 cells and SNU-423 cells cultured at LCD or HCD. Scale bars, 20 µm. (D-F) Immunoblot analysis of Flag-tagged Myr-NUMB3 and GAPDH (D), representative images of EdU assays (E) and colonies formation assays (F) of SNU-423 cells stably expressing vector or Myr-NUMB3. Scale bar, 50 µm. (G) The percentage of EdU positive cells or colonies number in SNU-423 cells stably expressing vector or Myr-NUMB3. Data are presented as mean ± SD and the *P* value of two-tailed unpaired Student's t test. (H-J) SNU-423 cells stably expressing vector or Flag-NUMB1-4 were transplanted into athymic mice (n = 6), 4 weeks after, image of tumor xenografts (H), tumor weight (I) and expression of indicated proteins (J) are shown. (K-N) SNU-423 cells stably expressing shControl or shRNA targeting NUMB3/4 were subcutaneously inoculated into athymic mice (n = 6), 4 weeks after, image of tumor xenografts (K), tumor weight (L), immunoblot (M) and qPCR (N) analysis of indicated proteins/genes are shown. Data are presented as mean ± SD and the *P* value was assessed using one-way ANOVA followed by Tukey's multiple comparisons test in I, L and N.
